# Targeted Repolarization of Tumor‐Associated Macrophages via Imidazoquinoline‐Linked Nanobodies

**DOI:** 10.1002/advs.202004574

**Published:** 2021-03-08

**Authors:** Evangelia Bolli, Maximilian Scherger, Sana M. Arnouk, Ana Rita Pombo Antunes, David Straßburger, Moritz Urschbach, Judith Stickdorn, Karen De Vlaminck, Kiavash Movahedi, Hans Joachim Räder, Sophie Hernot, Pol Besenius, Jo A. Van Ginderachter, Lutz Nuhn

**Affiliations:** ^1^ Lab of Cellular and Molecular Immunology Vrije Universiteit Brussel Pleinlaan 2 Brussels 1050 Belgium; ^2^ Myeloid Cell Immunology Lab VIB Center for Inflammation Research Brussels 1050 Belgium; ^3^ Max Planck Institute for Polymer Research Ackermannweg 10 Mainz 55128 Germany; ^4^ Department of Chemistry Johannes Gutenberg‐University Mainz Duesbergweg 10‐14 Mainz 55128 Germany; ^5^ Laboratory of In Vivo Cellular and Molecular Imaging Vrije Universiteit Brussel Laarbeeklaan 103 Brussels 1090 Belgium

**Keywords:** cancer immunotherapy, drug delivery, M2 macrophages, nanobodies, repolarization, TLR 7/8 agonist, tumor associated macrophages

## Abstract

Tumor‐associated macrophages (TAMs) promote the immune suppressive microenvironment inside tumors and are, therefore, considered as a promising target for the next generation of cancer immunotherapies. To repolarize their phenotype into a tumoricidal state, the Toll‐like receptor 7/8 agonist imidazoquinoline IMDQ is site‐specifically and quantitatively coupled to single chain antibody fragments, so‐called nanobodies, targeting the macrophage mannose receptor (MMR) on TAMs. Intravenous injection of these conjugates result in a tumor‐ and cell‐specific delivery of IMDQ into MMR^high^ TAMs, causing a significant decline in tumor growth. This is accompanied by a repolarization of TAMs towards a pro‐inflammatory phenotype and an increase in anti‐tumor T cell responses. Therefore, the therapeutic benefit of such nanobody‐drug conjugates may pave the road towards effective macrophage re‐educating cancer immunotherapies.

## Graphical abstract

Imidazoquinolines as toll‐like receptor 7/8 agonists can cell‐specifically be delivered via a macrophage mannose receptor targeting nanobodies to pro‐tumoral macrophages in the tumor microenvironment. A repolarization of their phenotype into a pro‐inflammatory state is observed affording significant decline in tumor growth. Such imidazoquinoline‐linked nanobody‐drug conjugates can be considered as promising macrophage re‐educating cancer immunotherapies.

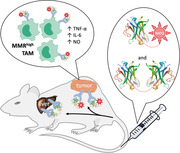

## 1 Introduction

Macrophages are often an integral part of the tumor immune compartment, playing key roles in tumor progression, metastasis, and resistance to anti‐cancer drugs, including immune checkpoint blockers.^[^ ^1^, ^2^, ^3^ ^]^ As a consequence, tumor‐associated macrophages (TAMs) are considered as promising targets for the next generation of cancer immunotherapies.^[^ ^3^, ^4^ ^]^ TAMs are versatile cells, which, depending on their microenvironment and localization within the tumor, can adopt different phenotypes.^[^ ^5^, ^6^ ^]^ In most cancers, tumor‐promoting TAMs dominate the tumor microenvironment (TME) resulting in a worse prognosis for the patient.^[^ ^1^ ^]^ Hence, there is a clear need to develop novel strategies to either deplete or – better – re‐educate these cells into their anti‐tumoral counterparts.

In this respect, TAMs with a high expression of the macrophage mannose receptor (MMR, CD206) were shown to exhibit a strong angiogenic and immune suppressive activity, identifying them as pro‐tumoral cells and, hence, as interesting targets for cancer therapy.^[^ ^7^ ^]^ MMR^high^ MHC‐II^low^ TAMs reside in hypoxic regions of the tumor or, following chemotherapy, along blood vessels and their characteristics are regulated by the hypoxic microenvironment and M‐CSF.^[^ ^8^, ^9^ ^]^ Monoclonal antibodies (mAbs) could be one way of specifically targeting these TAMs, but these moieties have relatively large molecular weights (∼150 kDa) which further increase upon coupling to other molecules. In comparison to smaller constructs, mAbs or mAb‐drug conjugates penetrate tumors less efficiently^[^ ^10^ ^]^ and may barely reach TAMs hiding in stroma thick areas. Besides, mAbs may aspecifically bind via their Fc part to Fc receptors expressed on various macrophages, hampering their specificity and potentially resulting in unwanted side effects.^[^ ^11^, ^12^, ^13^ ^]^

Conversely, nanobodies (Nbs), which are the smallest naturally occurring intact antigen binding fragments (∼15 kDa) derived from the variable domain of heavy chain only camelid Abs,^[^ ^14^, ^15^ ^]^ lack an Fc region and possess high stability, solubility, specificity, and tissue penetration.^[^ ^16^, ^17^, ^18^, ^19^ ^]^ In addition, Nbs can be engineered toward site‐selective chemical modification^[^ ^20^ ^]^ allowing the introduction of chemoselective linkers instead of nonspecific conjugation^[^ ^21^, ^22^ ^]^ that might potentially interfere with binding properties of the Nb.^[^ ^23^, ^24^, ^25^ ^]^ These characteristics encouraged their use for various therapeutic and diagnostic purposes.^[^ ^26^ ^]^

We previously developed an anti‐MMR Nb which showed efficacy in targeting MMR^high^ TAMs, in vitro and in vivo.^[^ ^27^, ^28^, ^29^, ^30^, ^31^, ^32^ ^]^ When coupled to radioactive tracers, this Nb has been successfully utilized for molecular imaging purposes^[^ ^29^, ^30^ ^]^ and for radioimmunotherapy of murine tumors where it outcompeted the efficacy of several currently used therapies.^[^ ^31^ ^]^ However, the use of anti‐MMR Nbs for the repolarization of TAMs towards an anti‐tumoral phenotype has not been explored. In this manuscript, we developed a site‐specific conjugation to directly conjugate anti‐MMR Nb to the highly potent imidazoquinoline variant 1‐(4‐(aminomethyl)benzyl)‐2‐butyl‐1*H*‐imidazo[4,5‐c]quinolin‐4‐amine (IMDQ).^[^ ^33^ ^]^ Imidazoquinolines (IMQs) such as imiquimod (trade name: Aldara) and resiquimod (R848) are ligands for the human Toll‐like receptor 7 (TLR7) and TLR8, as well as the mouse TLR7. TLR7 and TLR8 are endosomal receptors that exist in several immune cell types including macrophages. Upon ligand recognition, TLR7/8 stimulate NF‐*κ*B and other transcription factors in a MyD88‐dependent fashion, which in turn triggers the production of pro‐inflammatory cytokines and chemokines.^[^ ^34^ ^]^ Hence, TLR7/8 ligands are potent stimulators of the inflammatory macrophage phenotype.

Notably, recent research showed that TLR7/8 agonist‐loaded nanoparticles could be potent drivers of TAM re‐education into the pro‐inflammatory phenotype^[^ ^35^ ^]^ and of dendritic cell (DC) activation in the tumor and the tumor‐draining lymph nodes,^[^ ^36^, ^37^, ^38^ ^]^ resulting in significant anti‐tumor immunity and therapeutic efficacy. Although these studies have demonstrated the use of nanoparticles as a method to restrict immune activation to the specific organs, development of a molecularly targeted therapy that can precisely deliver TLR7/8 agonists exclusively to pro‐tumoral TAMs in vivo is of paramount interest to assure selective cellular delivery.

**Figure 1:**
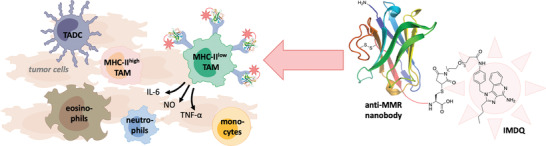
A well‐defined protein‐drug conjugate of anti‐MMR nanobody with the TLR 7/8 agonist IMDQ (1‐(4‐(aminomethyl)benzyl)‐2‐butyl‐1*H*‐imidazo[4,5‐c]quinolin‐4‐amine). Within the tumor immune compartment, the macrophage mannose receptor (MMR) is particularly expressed on the non‐inflammatory pro‐tumoral MHC‐II^low^ macrophage population among tumor associated macrophages (TAM). The anti‐MMR Nb‐IMDQ conjugate allows the triggering of TLR7/8 specifically of MMR^high^ macrophages, with the aim to repolarize these cells into a pro‐inflammatory anti‐tumoral state, resulting in a reduced tumor growth. (TADC: tumor associated dendritic cells.)

In this article, we show that the highly potent IMQ variant, IMDQ, can reprogram the pro‐tumoral TAMs into a pro‐inflammatory state and have developed an improved methodology to site‐specifically and quantitatively couple IMDQ to anti‐MMR Nbs (**Figure** **1**). Most importantly, the anti‐MMR Nb‐IMDQ conjugate led to efficient drug delivery to the MMR^high^ TAMs in vivo, causing a significant decline in tumor growth, paralleled by a repolarization of TAMs towards a pro‐inflammatory phenotype and an increase in anti‐tumor T cell responses.

## 2 Results

### 2.1 IMDQ Repolarizes Pro‐Tumoral TAMs In Vitro

Small molecule IMQs like imiquimod or resiquimod have been reported as potent TLR7/8 agonists.^[^ ^39^ ^]^ For this study, we chose 1‐(4‐(aminomethyl)benzyl)‐2‐butyl‐1H‐imidazo[4,5‐c]quinolin‐4‐amine (IMDQ) as a highly potent IMQ variant with a primary amine^[^ ^33^, ^40^, ^41^ ^]^ that enables chemical conjugation to macromolecules.^[^ ^36^, ^42^, ^43^, ^44^ ^]^ IMDQ could be synthesized following previously reported protocols^[^ ^45^ ^]^ with some minor adjustments, as documented in the Supporting Information.

**Figure 2:**
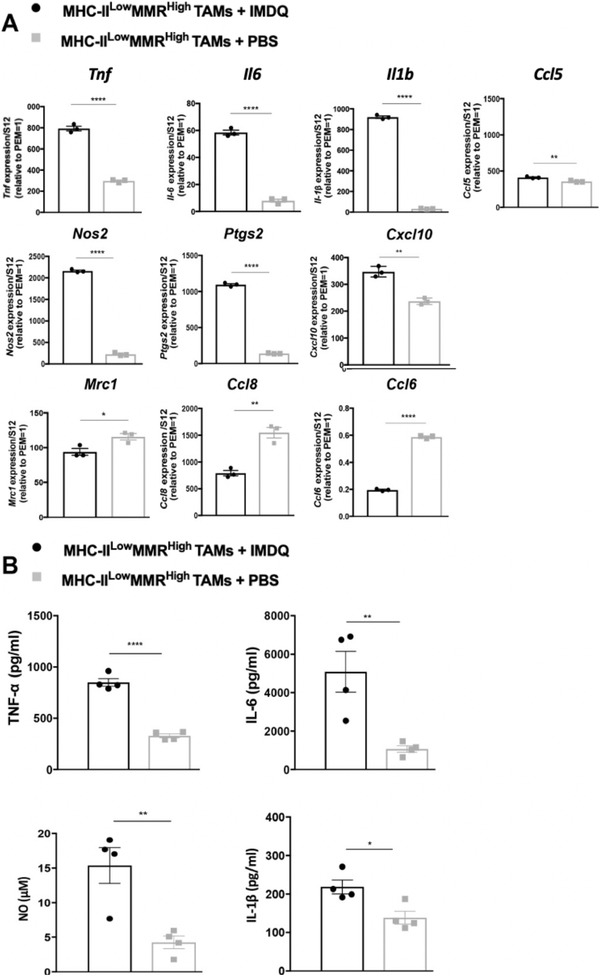
IMDQ induces a pro‐inflammatory phenotype in MHC‐II^low^MMR^high^ TAMs. MHC‐II^low^MMR^high^ TAMs from eight 13‐day‐old 3LL‐R tumors were sorted and incubated with either ∼10 µm IMDQ or PBS for 48 h at 37 °C. A) The gene expression profile was analyzed using quantitative real‐time polymerase chain reaction (qRT‐PCR). The expression of each gene was normalized based on the S12 housekeeping gene and was calculated relative to the expression level of the gene in freshly isolated peritoneal exudate macrophages (PEM = 1). The results are representative of 3–4 independent experiments and data is shown as mean ± standard error of the mean (SEM) of technical triplicates. B) Secretion levels of TNF‐*α* (pg ml^−1^), IL‐6 (pg ml^−1^), IL‐1*β* (pg ml^−1^), and NO (µm) were quantified by enzyme linked immunosorbent assay (ELISA) and NO assay, respectively. Data is shown as mean ± SEM of *n* = 4 biological replicates. Student's *t*‐test was performed to compare IMDQ‐treated MHC‐II^low^MMR^high^ TAMs to PBS‐treated MHC‐II^low^MMR^high^ TAMs. *p*‐Values are calculated using an unpaired Student's *t*‐test and significant differences are marked by *: *p* ≤ 0.05; **: *p* ≤ 0.01; ****: *p* ≤ 0.0001.

Prior to further chemical modification and site‐specific conjugation to Nbs, IMDQ was examined for its ability to reprogram the phenotype of pro‐tumoral, anti‐inflammatory TAMs in vitro. To this extent, CD11b^high^Ly6C^low^MHC‐II^low^ TAMs, representing the pro‐tumoral TAM population in various mouse tumor models,^[^ ^7^ ^]^ were isolated from subcutaneously grown 3LL‐R lung carcinoma tumors according to our previously reported gating strategy^[^ ^7^ ^]^ (Figure S1, Supporting Information) and cultured in the presence of ∼10 µM free IMDQ for 48 h. Of note, MHC‐II^low^ TAMs are also known to express high levels of MMR.^[^ ^7^ ^]^ IMDQ‐treated MHC‐II^low^MMR^high^ TAMs upregulated the expression of genes typically associated with pro‐inflammatory TAMs (*Tnf, Il6, Il1b, Ccl5, Nos2, Ptgs2*, and *Cxcl10*), while lowering the expression of genes associated with anti‐inflammatory TAMs (*Mrc1, Ccl8*, and *Ccl6*), compared to control MHC‐II^low^MMR^high^ TAMs (**Figure** **2**A). These data were corroborated at the protein level, with the secretion of TNF‐*α*, IL‐6, IL‐1*β*, and NO being boosted in IMDQ‐stimulated MHC‐II^low^MMR^high^ TAMs (Figure 2B). Together, these results prove the potential of IMDQ to reprogram the anti‐inflammatory MHC‐II^low^MMR^high^ TAM phenotype into a more pro‐inflammatory state.

### 2.2 Bioconjugation of IMDQ to Anti‐MMR Nb

We next aimed to employ IMDQ for targeted cancer therapy, necessitating the use of a carrier to deliver IMDQ specifically to the pro‐tumoral TAMs in the TME in order to avoid systemic inflammatory side effects. This is especially important since we favor an intravenous route of administration (instead of intra‐ or peri‐tumoral injection) in order to provide therapeutic strategies also for hard‐to‐reach cancers and their metastases. We previously reported the use of an anti‐MMR Nb for pro‐tumoral TAM targeting,^[^ ^31^ ^]^ suggesting this Nb as a fit carrier.

**Figure 3:**
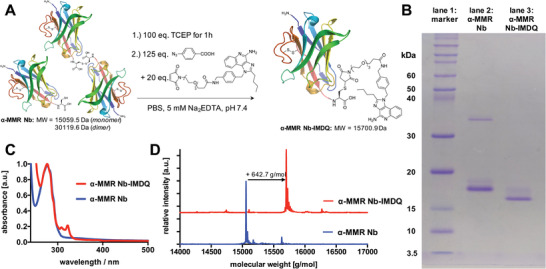
IMDQ‐conjugated anti(*α*)‐MMR Nb. A) Synthetic scheme for the modification of mono‐ and dimeric C‐terminal cysteine tagged *α*‐MMR Nbs with IMDQ‐PEG_4_‐maleimide. B) Coomassie stained non‐reducing SDS‐PAGE of *α*‐MMR Nb (lane 2, monomer *M*_W_ = 15 059.5 Da, dimer *M*_W_ = 30 119.6 Da) pre‐ and post‐modified as *α*‐MMR Nb‐IMDQ (lane 3, *M*_W_ = 15 701.9 Da). C) UV–vis spectrum of *α*‐MMR Nb (blue) and *α*‐MMR Nb‐IMDQ (red)—note that the characteristic imidazoquinoline absorbance peaks around 308 and 322 nm appear stoichiometrically. D) ESI‐Q‐TOF‐MS profile (deconvoluted) of *α*‐MMR Nb (blue) and *α*‐MMR Nb‐IMDQ (red). The shift in molecular weight from 15 059.5 Da for *α*‐MMR Nb (found: 15 058.1 Da) to 15 701.9 Da for *α*‐MMR Nb‐IMDQ (found: 15 700.9 Da) corresponds to the molecular weight of IMDQ‐maleimide (642.7 g·mol^−1^) and thus indicates successful mono‐functionalization of the Nb (theoretic molecular weights calculated by ExPASy web portal).

Thus, IMDQ was chemically modified with a short oligoethylene glycol linker (PEG_4_) terminated by a maleimide group for site‐specific conjugation to cysteine‐tagged anti‐MMR Nbs, as described in detail in the Supporting Information (schematized in **Figure** **3**A). Although IMDQ‐PEG_4_‐maleimide showed a reduced TLR7/8 stimulating potency as compared to unmodified IMDQ, receptor stimulation was still prominent, as verified in a RAW Blue macrophage NF‐*κ*B reporter assay (Figure S25, Supporting Information).

We hypothesized that, for therapeutic purposes, the reduction in TLR7/8‐triggering potency would be compensated for by the targeted delivery of IMDQ‐PEG_4_‐maleimide to the responder cells, that is, the pro‐tumoral MMR^high^ TAMs. Hence, the pendant maleimide group was conjugated to free and accessible sulfhydryls/thiols on the anti‐MMR Nbs via a Michael‐type addition reaction.^[^ ^46^ ^]^ To avoid a disruption of the stabilizing internal disulfide bridge in the Nb, we used genetically engineered Nbs with a C‐terminal cysteine tag (Figures S4–S6, Supporting Information) and optimized our protocols for its site‐selective modification with maleimides.

In brief, anti‐MMR Nb dimer was reduced to its monomers by incubation with 100 equivalents of tris(2‐carboxyethyl)phosphine (TCEP) (illustrated in Figure 3A). Excess of TCEP did not affect the internal disulfide bond but hindered efficient Michael‐type conjugation with maleimides (compare Figure S7, Supporting Information). This, however, could be resolved by quenching excess TCEP after disulfide reduction with 125 equivalents of 4‐azidobenzoic acid (ABA).^[^ ^47^ ^]^ Simultaneously, 20 equivalents of the maleimide component could be added to selectively address the C‐terminal cysteine of the anti‐MMR Nb. The final product could be purified via size exclusion chromatography (SEC) and the obtained fraction was analyzed by SDS‐PAGE, UV–vis spectroscopy and by ESI‐Q‐TOF mass spectrometry (MS) (Figure 3B–D). This protocol was applicable for the anti‐MMR Nb as well as the BCII10 Nb, which was used in further experiments as a non‐targeting negative control. In addition, the same protocol could be used to covalently install other types of functional maleimides, such as Alexa Fluor (AF) 647‐maleimide (Figures S7–S9, Supporting Information), near infrared dye (NIRdye) 800CW‐maleimide (Figures S10–S14, Supporting Information), and AF488‐maleimide (Figures S15–S19, Supporting Information).

As shown in Figure 3B, the starting material for the modification was a mixture of dimers (*M*_W_ = ∼30 119.06 Da) and monomers (*M*_W_ =∼ 15 059.53 Da) of anti‐MMR Nb (lane 2), while after modification with IMDQ‐PEG_4_‐maleimide a single band corresponding to the size of anti‐MMR Nb‐IMDQ (lane 3; *M*_W_ = ∼15 701.85 Da) was found by non‐reducing SDS‐PAGE. Similar results were found for Nbs after modification with AF488‐/AF647‐ or NIRdye 800CW‐maleimide (Figures S7, S10, S15, and S20, Supporting Information). Of note, IMDQ is a hydrophobic compound and consequently leads to a faster migration of the anti‐MMR Nb‐IMDQ conjugate compared to the unconjugated anti‐MMR Nb monomer during SDS‐PAGE. As observed by UV–vis spectroscopy (Figure 3C), only an equimolar amount of IMDQ was ligated to anti‐MMR Nb (see Figures S8, S11, S13, S16, S18, and S22, Supporting Information, for all other species). Thus, no unconjugated anti‐MMR Nb was present, nor a double or triple modification that would occur in case of cleavage of the Nb's internal disulfide bridge. Consequently, this optimized conjugation protocol seems to be highly selective and efficient. These results were further confirmed by ESI‐Q‐TOF MS, in which a single peak was observed at 15 701.9 Da corresponding to the anti‐MMR Nb‐IMDQ. This peak was shifted compared to the profile of unmodified anti‐MMR Nb at 15 058.1 Da, in accordance with the molecular weight of IMDQ‐PEG_4_‐maleimide of 642.7 Da (Figure 3D). Similar mass spectrometry results were obtained for the BCII10 Nb‐IMDQ, as well as for all other modifications with the fluorescent maleimides (see Supporting Information Figures S4–S6, S9, S12, S14, S17, S19, S21, and S23, Supporting Information). These data demonstrate that both the anti‐MMR Nb and its control BCII10 Nb could successfully and directly be conjugated to one single IMDQ molecule via site‐selective modification.

### 2.3 Site‐Selectively Modified Anti‐MMR Nb Specifically Targets the MHC‐II^low^MMR^high^TAMs, with Anti‐MMR Nb‐IMDQ Increasing their Inflammatory Phenotype

**Figure 4:**
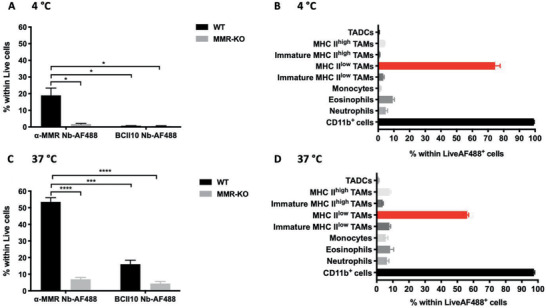
Conjugated anti (*α*)‐MMR Nb targets MHC‐II^low^MMR^high^ TAMs. Single cell suspensions from 14‐day‐old 3LL‐R tumors grown in WT and MMR‐KO C57Bl/6 mice were obtained and incubated with 10 µg mL^−1^ (∼0.64 µm) of *α*‐MMR Nb‐AF488 or BCII10 Nb‐AF488 for 2 h at A,B) 4 °C and C,D) 37 °C, followed by FACS analysis. The percentage of Nb‐AF488‐labelled live cells from tumors grown in WT and MMR‐KO C57Bl/6 mice (A,C). Distribution of *α*‐MMR Nb‐AF488^+^ cells within different immune cell populations from tumors grown in WT C57Bl/6 mice (B,D). The gating strategy followed in FACS for the different myeloid subsets is illustrated in Figure S1, Supporting Information. Data is shown as mean ± SEM of *n* = 3 biological replicates. The result of single cell suspensions of tumors grown in WT C57Bl/6 mice and incubated with *α*‐MMR Nb‐AF488 is compared against the results obtained for all other groups and *p*‐values are calculated using an unpaired Student's *t*‐test and significant differences are marked by *: *p* ≤ 0.05; ***: *p* ≤ 0.001; ****: *p* ≤ 0.0001. (TADCs: Tumor‐associated dendritic cells.)

In the first instance, we wished to ascertain that the modification protocol did not affect the functionality of the anti‐MMR Nb. Hence, we coupled the AF488 dye to the anti‐MMR Nb or to the control BCII10 Nb via site‐selective modification (Figures S15–S19, Supporting Information), following the same chemistry as used for the synthesis of anti‐MMR Nb‐IMDQ (Figure 3A). Next, tumor single cell suspensions were prepared from 3LL‐R tumors, grown in wildtype (WT) or MMR‐knock out (KO) C57BL/6 mice, and incubated with anti‐MMR Nb‐AF488 or with BCII10 Nb‐AF488 for 2 h at 4 °C (only surface staining, **Figure** **4**A,B) or at 37 °C (surface staining and cellular uptake, Figure 4C,D). At both temperatures, anti‐MMR Nb‐AF488 binding was restricted to CD11b^+^ myeloid cells and mostly confined to MHC‐II^low^ (MMR^high^) TAMs isolated from WT mice, while the staining of cells retrieved from MMR‐KO tumors was significantly lower. Moreover, anti‐MMR Nb‐AF488 staining strongly superceded the staining with BCII10‐AF488. Note that minor staining of other CD11b^+^ cells, which express relatively lower MMR levels as illustrated by their lower ΔMFI (Figure S27, Supporting Information), can be observed. Note also that Nb staining was overall higher at 37 °C than at 4 °C, including in MMR‐KO mice, indicating some level of aspecific Nb uptake at physiological temperature (Figure 4A,C; Figure S26, Supporting Information). Nevertheless, MHC‐II^low^ TAMs clearly remain the major target cell of the anti‐MMR Nb in an MMR‐dependent manner, indicating that the maleimide‐mediated cysteine tag‐selective modification of the anti‐MMR Nb does not limit its targeting potential.

**Figure 5:**
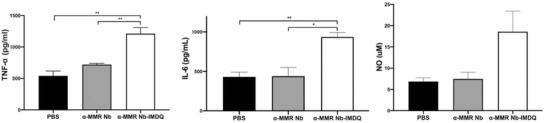
Anti (*α*)‐MMR Nb‐IMDQ induces the secretion of pro‐inflammatory mediators from MHC‐II^low^MMR^high^ TAMs. MHC‐II^low^MMR^high^ TAMs from eight 13‐day‐old 3LL‐R tumors were sorted and incubated with either PBS or ∼2.5 µm *α*‐MMR Nb‐IMDQ or *α*‐MMR Nb for 48 h at 37 °C. Afterwards, culture supernatants were collected and the secretion levels of TNF‐*α* (pg ml^−1^), IL‐6 (pg ml^−1^), and NO (µm) were quantified by ELISA and NO assay, respectively. Data is shown as mean ± SEM of *n* = 3 biological replicates. *p*‐Values are calculated using unpaired Student's *t*‐test and significant differences are marked by *: *p* ≤ 0.05; **: *p* ≤ 0.01.

We finally assessed whether anti‐MMR Nb‐IMDQ was able to convert MHC‐II^low^MMR^high^ TAMs into more inflammatory cells. Isolated MHC‐II^low^MMR^high^ TAMs from 3LL‐R tumors were cultured for 48 h at 37 °C in the presence of anti‐MMR Nb‐IMDQ, using equimolar amounts of unconjugated anti‐MMR Nb and PBS as controls. Importantly, only anti‐MMR Nb‐IMDQ was able to induce the release of inflammatory mediators, such as TNF‐*α*, IL‐6, and NO, above background levels (PBS condition) from these TAMs (**Figure** **5**). In conclusion, these results show that: i) the anti‐MMR Nb as such is unable to alter the macrophage activation state, in line with our earlier findings^[^ ^29^ ^]^; and ii) the Nb‐coupled IMDQ is still functional and encouraged further evaluation of the anti‐MMR Nb‐IMDQ conjugate in vivo.

### 2.4 Assessment of the In Vivo Biodistribution and Specificity of Anti‐MMR Nb‐IMDQ Using Pinhole SPECT/*μ*CT and Ex Vivo Dissection

**Figure 6:**
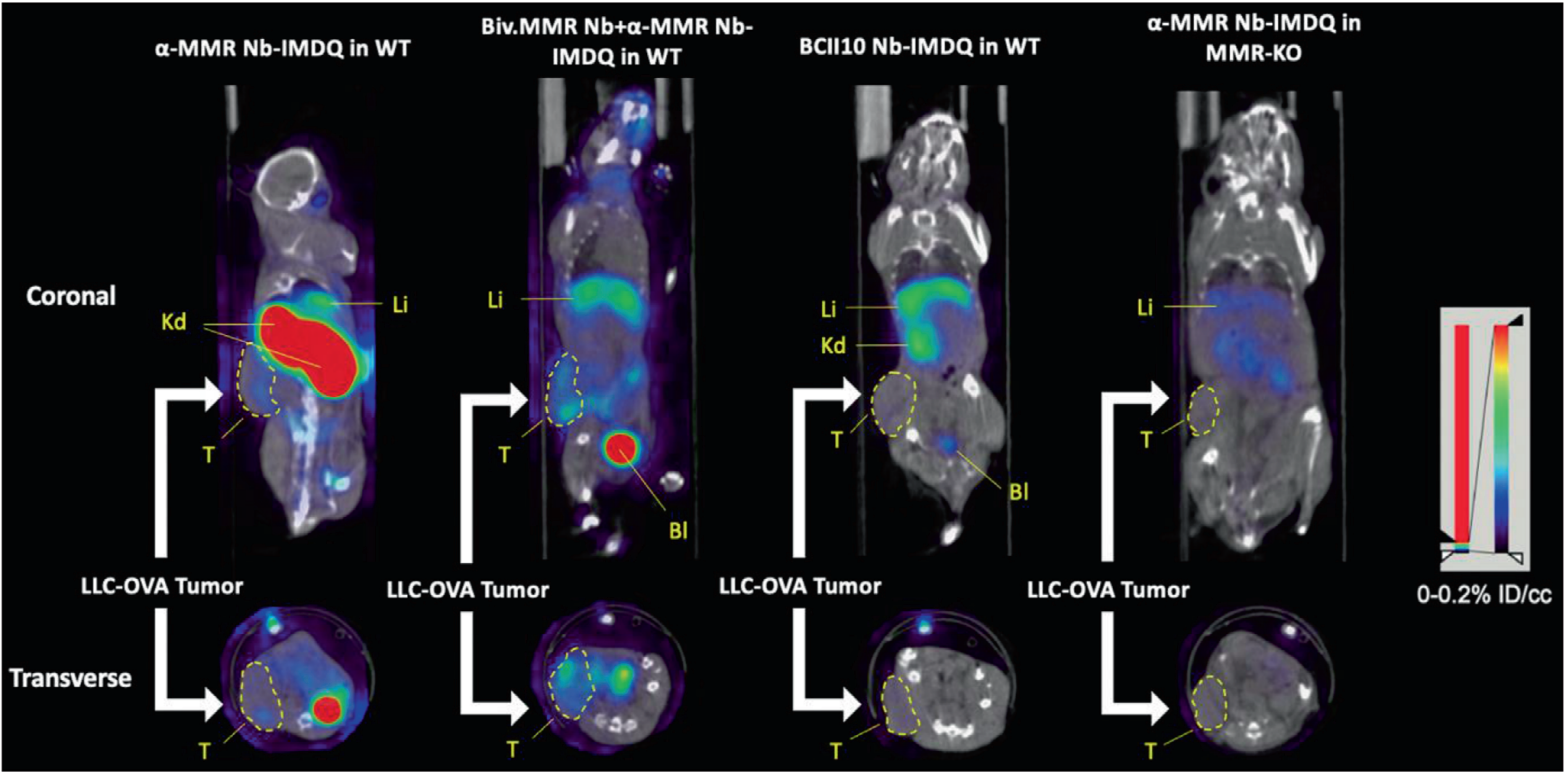
In vivo biodistribution of ^99m^Tc‐anti (*α*)‐MMR Nb‐IMDQ in LLC‐OVA‐bearing mice using fused pinhole SPECT/µCT. SPECT/µCT images of (from left to right): s.c. LLC‐OVA‐bearing WT mouse injected with ^99m^Tc‐*α*‐MMR Nb‐IMDQ, s.c. LLC‐OVA‐bearing WT mouse co‐injected with ^99m^Tc‐*α*‐MMR Nb‐IMDQ and fivefold molar excess of unlabeled bivalent *α*‐MMR Nb (Biv.MMR Nb), s.c. LLC‐OVA‐bearing WT mouse injected with ^99m^Tc‐BCII10 Nb‐IMDQ, and s.c. LLC‐OVA‐bearing MMR‐KO mouse injected with ^99m^Tc‐*α*‐MMR Nb‐IMDQ. Imaging was performed 3 h p.i. using VECTor and images were acquired using AMIDE Medical Image Data Examiner software. Coronal and transverse views are shown. The results are representative of 3 mice. Kd: Kidneys; Li: Liver; T: Tumor; Bl: Bladder. Tumor is indicated by a dashed line.

**Figure 7:**
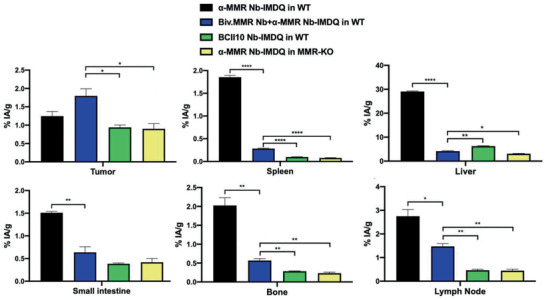
^99m^Tc tracer uptake in different organs and in the tumor. S.c. LLC‐OVA‐bearing WT and MMR‐KO mice were injected with different ^99m^Tc‐labeled Nb constructs as indicated in the graphs and uptake values (%IA/g) were measured 3 h p.i. via organ dissection and *γ*‐counting. Data is shown as mean ± SEM of 3 biological replicates. The result of the group co‐injected with ^99m^Tc‐anti (*α*)‐MMR Nb‐IMDQ and the bivalent *α*‐MMR Nb is compared against the result obtained for all other groups and *p*‐values are calculated using an unpaired Student's *t*‐test and significant differences are marked by *: *p* ≤ 0.05; **: *p* ≤ 0.01; ****: *p* ≤ 0.0001.

Next, we assessed whether anti‐MMR Nb‐IMDQ could accumulate in the tumor. We chose another Lewis Lung Carcinoma variant for these studies, LLC‐OVA, as this model is more immunogenic and consequently more amenable to immunotherapeutic intervention. For in vivo imaging, the *γ*‐emitter [^99m^Tc(H_2_O)_3_(CO)_3_]^+^ was complexed by the hexahistidine sequence of the anti‐MMR Nb‐IMDQ conjugate, following an established radiolabeling procedure, to enable single‐photon emission computed tomography/microCT (SPECT/*μ*CT) imaging.^[^ ^29^, ^48^, ^49^ ^] 99m^Tc‐anti‐MMR Nb‐IMDQ was injected intravenously in LLC‐OVA tumor‐bearing WT or MMR‐KO mice with or without a fivefold molar excess of cold, non‐labelled bivalent anti‐MMR Nb. Of note, the co‐injection of an excess of bivalent anti‐MMR Nb was reported before to diminish the extratumoral uptake of the monovalent anti‐MMR Nb, while increasing its uptake in the tumor.^[^ ^29^, ^31^ ^] 99m^Tc‐BCII10 Nb‐IMDQ was used as a negative non‐targeting control. ^99m^Tc‐anti‐MMR Nb‐IMDQ alone accumulated in an MMR‐dependent manner in the liver, spleen, small intestine, bone, and lymph nodes, while the uptake in the tumor was only slightly higher as compared to ^99m^Tc‐BCII10 Nb‐IMDQ (**Figures** **6** and **7**; Table S2, Supporting Information). However, upon co‐injection of bivalent anti‐MMR Nb, the uptake of ^99m^Tc‐anti‐MMR Nb‐IMDQ in all organs strongly decreased, while its accumulation in the tumor increased (Figures 6 and 7; Table S2, Supporting Information). Hence, the anti‐MMR Nb‐IMDQ conjugate can target the tumor microenvironment in an MMR‐specific manner, provided that an excess of bivalent anti‐MMR Nb is co‐administered. Importantly, CD45^−^ cancer cells are MMR‐negative (Figure S30, Supporting Information), ascertaining that the anti‐MMR Nb‐IMDQ targets CD45^+^ hematopoietic cells, of which the pro‐tumoral MHC‐II^low^ TAMs are the most prominent MMR‐expressors.

Notably, the same conclusions could be drawn when using the anti‐MMR Nb or the control BCII10 Nb coupled via site‐selective modification to the near infrared dye NIRdye 800CW (Figures S10–S14, Supporting Information), following the same chemistry as used for the synthesis of anti‐MMR Nb‐IMDQ (Figure 3A). Indeed, near infrared fluorescence based biodistribution studies in LLC‐OVA tumor‐bearing mice qualitatively confirmed the observations, after 21 h, we obtained by radioactive labeling of therapeutically active IMDQ‐Nbs after 3 h (Figures S28 and S29, Supporting Information).

### 2.5 Anti‐MMR Nb‐IMDQ Therapy Repolarizes MHC‐II^low^MMR^high^ TAMs In Vivo and Significantly Reduces LLC‐OVA Tumor Growth

**Figure 8:**
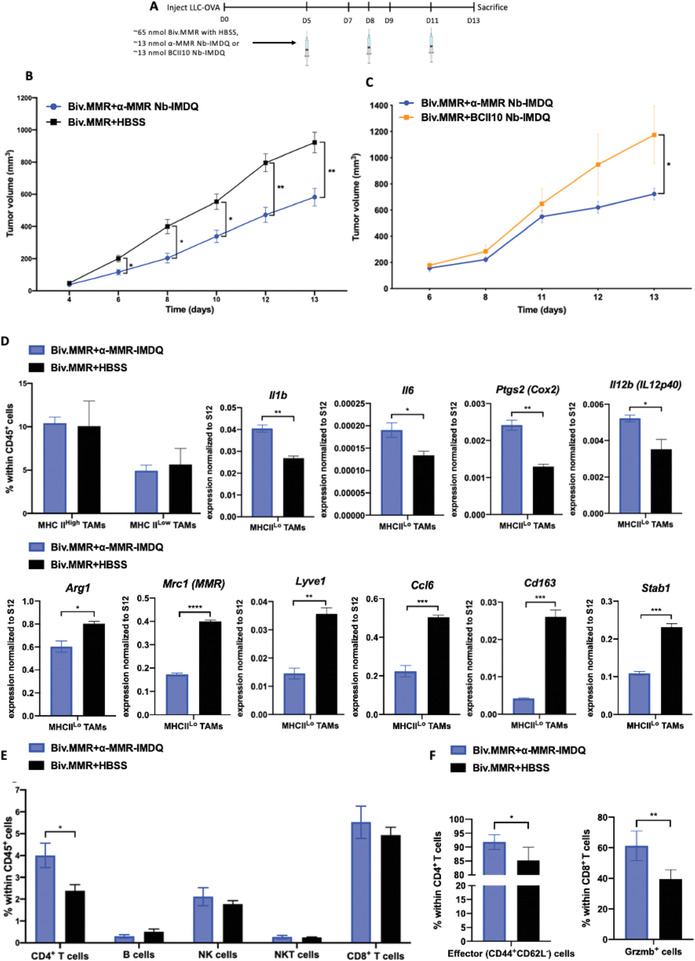
Anti (*α*)‐MMR Nb‐IMDQ therapy delays tumor progression and reprograms TAMs to a more M1‐like phenotype. A) LLC‐OVA‐bearing C57BL/6 mice were injected on day 5, 8, and 11 after cancer cell inoculation with the appropriate treatment and mice were sacrificed on day 13. B) LLC‐OVA‐bearing mice received *α*‐MMR Nb‐IMDQ or HBSS, co‐injected with fivefold molar excess of the bivalent *α*‐MMR Nb (Biv.MMR) and tumor volumes were measured on day 4, 6, 8, 10, 12, and 13 after cancer cell inoculation. Data is shown as mean ± SEM of 7 (Biv.MMR+*α*‐MMR‐IMDQ) to 8 (Biv.MMR+HBSS) biological replicates. *p*‐Values are calculated using a two‐way ANOVA and significant differences are marked by *: *p* ≤ 0.05; **: *p* ≤ 0.01. C) LLC‐OVA‐bearing mice received *α*‐MMR Nb‐IMDQ or BCII10 Nb‐IMDQ, co‐injected with fivefold molar excess of the Biv.MMR and tumor volumes were measured on day 6, 8, 11, 12, and 13 after cancer cell inoculation. Data is shown as mean ± SEM of 6 (Biv.MMR+*α*‐MMR‐IMDQ) to 5 (Biv.MMR+BCII10 Nb‐IMDQ) biological replicates. *p*‐Values are calculated using a two‐way ANOVA and significant differences are marked by *: *p* ≤ 0.05. D) The percentage of MHC‐II^high^ and MHC‐II^low^ TAMs within the hematopoietic (CD45^+^) cells of LLC‐OVA tumors is shown as mean ± SEM of *n* = 4 biological replicates. MHC‐II^low^ TAMs were sorted from pools of tumor cell suspensions of each individual experimental group and qRT‐PCR analysis was performed for technical triplicates to quantify the expression of several M1‐associated genes (*Il1b, Il6*, *Ptgs2*, and *Il12b*) and M2‐associated genes (*Arg1, Mrc1, Lyve1*, *Ccl6, Cd163*, and *Stab1*) normalized to ribosomal protein *S12* expression. Data is shown as mean ± SEM of *n* = 3 technical replicates. *p*‐Values are calculated using an unpaired Student's *t*‐test and significant differences are marked by *: *p* ≤ 0.05; **: *p* ≤ 0.01; ***: *p* ≤ 0.001; and ****: *p* ≤ 0.0001. E) The percentage of CD4^+^ T cells, B cells, NK cells, NKT cells, and CD8^+^ T cells within the hematopoietic (CD45^+^) cells is shown as mean ± SEM of *n* = 4 biological replicates. *p*‐Values are calculated using an unpaired Student's *t*‐test and significant differences are marked by *: *p* ≤ 0.05. F) The percentage of effector (CD44^+^CD62L^−^) cells within CD4^+^ T cells and Gzmb^+^ cells within CD8^+^ T cells is shown as mean ± SEM of *n* = 4 biological replicates. *p*‐Values are calculated using an unpaired Student's *t*‐test and significant differences are marked by *: *p* ≤ 0.05; **: *p* ≤ 0.01.

We finally assessed whether the tumor‐targeting potential of anti‐MMR Nb‐IMDQ and the potency of Nb‐coupled IMDQ were sufficient to affect tumor growth. As soon as palpable tumors became visible, LLC‐OVA tumor‐bearing mice were treated with 205 µg (∼13 nmol) of anti‐MMR Nb‐IMDQ (corresponding to 5.0 µg IMDQ) and a fivefold molar excess of bivalent anti‐MMR Nb, according to the schedule depicted in **Figure** **8**A. This treatment resulted in a significantly delayed LLC‐OVA tumor growth as compared to the control groups that either only received bivalent anti‐MMR Nb (Figure 8B) or received BCII10 Nb‐IMDQ (Figure 8C) instead of anti‐MMR Nb‐IMDQ. The anti‐MMR Nb‐IMDQ treatment resulted in a higher tumor infiltration of Ly6C^hi^ monocytes and a lower infiltration of neutrophils, with no effect on the presence of eosinophils or dendritic cells (Figure S31, Supporting Information). Moreover, this successful treatment did not affect the relative abundance of TAM subsets, but was paralleled by an alteration in the MHC‐II^low^ TAM phenotype, in which the expression of several prototypical M1‐related genes (*Il1b, Il6*, *Ptgs2*, and *Il12b*) was increased, while genes related to an M2‐like phenotype (*Arg1, Mrc1, Lyve1*, *Ccl6, Cd163*, and *Stab1*) were downregulated (Figure 8D). This was accompanied by an increase in the percentage of tumor‐infiltrating CD4^+^ T cells, but not B cells, NK cells, NKT cells, or CD8^+^ T cells (Figure 8E). Moreover, within the CD4^+^ T cell population, a higher contribution of CD44^+^CD62L^−^ effector cells, but not Foxp3^+^ Treg, was noticed upon anti‐MMR Nb‐IMDQ treatment (Figure 8F; Figure S32, Supporting Information). Likewise, within the CD8^+^ T cell population, the percentage of granzyme B^+^ effector CD8^+^ T cells (Figure 8F) was significantly higher in tumors of mice receiving anti‐MMR Nb‐IMDQ in comparison to the control group, suggesting an enhanced anti‐tumor immunity. These results clearly demonstrate the therapeutic effect of anti‐MMR Nb‐IMDQ on LLC‐OVA tumors, likely mediated by a repolarization of MHC‐II^low^ TAMs and an increased anti‐tumor T cell response.

Finally, we combined the anti‐MMR Nb‐IMDQ treatment with an anti‐PD1 immune checkpoint blockade. Both monotherapies significantly reduced LLC‐OVA tumor growth as compared to the control group (Bivalent anti‐MMR Nb + HBSS + isotype mAb) (Figure S33, Supporting Information). The combination therapy was on average not significantly different from both monotherapies. Nevertheless, upon combination treatment, 5/7 tumors reached a tumor volume < 500 mm^3^ after 13 days of tumor growth, while this is only 2/7 for anti‐MMRNb‐IMDQ monotherapy and 1/7 for anti‐PD1 monotherapy, suggesting a trend towards a more efficient therapy in the combination group.

## 3 Discussion

Immune‐based cancer therapies, mainly targeting T cell functions, have shown impressive clinical outcomes in a fraction of patients.^[^ ^50^ ^]^ Non‐responsive patients are inherently resistant or have developed resistance mechanisms that are, in part, attributed to TAMs.^[^ ^12^ ^]^ Therefore, therapies focused on TAMs are of high interest and could overcome limitations of current treatment options. In this context, functional re‐education of TAMs to a tumoricidal and/or immune‐permissive state is a promising approach^[^ ^51^, ^52^ ^]^ and recent research showed that TLR7/8 agonists are amongst the most efficient drugs in TAM re‐education.^[^ ^35^ ^]^

Here, we corroborated the capacity of IMDQ, an IMQ derivative and potent TLR7/8 agonist, to reprogram, in vitro, the strongly pro‐tumoral MHC‐II^low^MMR^high^ TAM subset into a more pro‐inflammatory phenotype, as demonstrated by an altered M1/M2‐like gene expression profile^[^ ^7^, ^9^ ^]^ and an increased secretion of inflammatory mediators that are known to be regulated by TLR7/8 signaling.^[^ ^34^, ^35^, ^53^, ^54^ ^]^ However, in an in vivo setting, IMQs display an unfavorable pharmacokinetic profile which does not allow them to reach optimal concentrations in the specific site of interest.^[^ ^42^ ^]^ In addition, they evoke systemic inflammation and dose‐limiting toxicity.^[^ ^36^ ^]^ To circumvent these limitations, new technologies have been used as delivery systems of TLR7/8 ligands, including lipidation approaches, encapsulating nanoparticles, adsorption to aluminum salts adjuvants, and conjugation to polymers.^[^ ^35^, ^36^, ^37^, ^42^, ^55^, ^56^, ^57^ ^]^ Nevertheless, these formulations cannot be considered as specific TAM‐targeted therapies. For instance, the uptake of IMDQ‐encapsulated nanoparticles was also mediated by DCs, monocytes, and B cells^[^ ^42^ ^]^ and their anti‐tumoral effect seem to be largely DC‐driven.^[^ ^36^ ^]^ We now demonstrate that the use of anti‐MMR Nbs as carrier allows a specific targeting of MHC‐II^low^MMR^high^ TAM, without affecting non‐myeloid cells or DCs.

Site‐specific conjugates of anti‐MMR Nb were achieved by optimizing C‐terminal cysteine modification via reduction with excess TCEP and subsequent quenching with ABA.^[^ ^47^ ^]^ Simultaneous addition of maleimide derivatized fluorescent dyes or IMDQ provided precise mono‐conjugates in quantitative yields. Importantly, this chemical modification did not interfere with the binding capacity of the anti‐MMR Nb to MHC‐II^low^MMR^high^ TAMs in vitro and in vivo. Moreover, the anti‐MMR Nb‐IMDQ conjugate was able to induce the release of several inflammatory mediators. Notably, the PEG_4_‐maleimide modification reduces IMDQ's capacity to stimulate TLR7/8, which is in line with the effect of other IMDQ chemical modifications reported in our previous studies.^[^ ^36^, ^44^ ^]^ However, this lowered IMDQ activity was compensated for by its direct targeting to the relevant cells, as demonstrated by the significant anti‐tumor effects of the anti‐MMR Nb‐IMDQ. As a matter of fact, the decreased TLR7/8 stimulating‐capacity of the anti‐MMR Nb‐IMDQ might even be advantageous to avoid off‐target effects and maximize the impact on MMR‐expressing cells. Indeed, a similar scenario has been shown previously for a mutated type I interferon with a decreased receptor affinity.^[^ ^58^ ^]^ The injection of the mutated cytokine conjugated to an Nb targeting Clec9A, expressed by XCR1^+^ cDC1s, in tumor‐bearing mice drastically decreased tumor growth, indicating that the activity of the mutated cytokine is selectively restored for cell populations expressing Clec9A, while minimizing systemic toxicity.

Using SPECT/*μ*CT, we demonstrated that the anti‐MMR Nb‐IMDQ conjugate could accumulate in the tumor in an MMR‐specific fashion and that IMDQ does not alter the in vivo biodistribution of the Nb as compared to previously obtained results.^[^ ^29^, ^31^ ^]^ Of note, this is the first demonstration of radioisotope‐labeling of an Nb conjugate. Although the use of excess bivalent anti‐MMR Nb as a strategy to block extra‐tumoral binding of monovalent anti‐MMR has been previously proposed,^[^ ^29^, ^31^ ^]^ we now show that the co‐injection of only a fivefold molar excess of the bivalent form was sufficient to achieve successful blocking and to obtain a similar tumor uptake of the monovalent form as achieved when co‐injecting a 20‐ or 100‐fold excess of the bivalent form.^[^ ^31^ ^]^ Importantly, we have previously shown that such tumor uptake levels of a ^177^Lu‐labeled anti‐MMR Nb was sufficient to achieve a slower tumor growth of murine TS/A breast carcinoma tumors^[^ ^31^ ^]^ (these tumor uptake values were also comparable to values previously published by Krüwel et al. for the injection of ^99m^Tc‐labeled anti‐EGFR Nbs in mice bearing EGFR‐expressing tumors^[^ ^18^ ^]^).

Having shown the capacity of the anti‐MMR Nb‐IMDQ to accumulate in the tumor, we next examined the therapeutic effect of this conjugate. We observed a significant decrease in LLC‐OVA tumor growth in an MMR‐dependent manner. Although there was no difference in the percentage of MHC‐II^low^ TAM within the immune compartment, these cells had adopted a more M1‐like anti‐tumoral phenotype. This is in line with our in vitro data showing that IMDQ can alter the phenotype of TAMs. Furthermore, this alteration was paralleled by an increase in the percentage of CD4^+^ T cells which encompass a larger fraction of effector cells, alongside an increase in granzyme B^+^ CD8^+^ T cells. Similar to our result, Smith et al. showed that topical application of the TLR7/8 ligand imiquimod for the treatment of invasive cutaneous squamous cell carcinoma caused an increase in granzyme B^+^ CD8^+^ T cells and polarized the lymphoid and monocyte/macrophage populations to a more Th1 and M1 cytokine pattern, respectively.^[^ ^59^ ^]^ These findings were further corroborated by Clark et al. who showed that topical imiquimod application caused an increase in effector T cells that produce more IFN‐*γ*, perforin, and granzyme.^[^ ^60^ ^]^ Interestingly, the same group also showed that changes in the cancerous lesion‐infiltrating T cells upon imiquimod treatment are indirect and must be mediated by other imiquimod‐responsive cell types.^[^ ^61^ ^]^ One of the candidate mediators could be TAMs, considering their proven susceptibility to TLR7/8 agonists. In fact, a shift in TAM phenotype towards an M1‐like state has been previously shown to be concomitant with an increase in T cell infiltration^[^ ^62^, ^63^, ^64^ ^]^ and activation.^[^ ^62^, ^64^, ^65^ ^]^ Even though interactions between TAMs and lymphoid cells in the TME have been previously documented,^[^ ^66^, ^67^ ^]^ we cannot conclude that the TAMs acquiring a more M1‐like phenotype in response to the anti‐MMR Nb‐IMDQ treatment are directly interacting with T cells. Another possibility could be that the change in TAM phenotype produces a more permissive TME, allowing the activation of T cells and the manifestation of their cytotoxic activity which might otherwise be impeded. In summary, our findings prove the therapeutic benefit of the anti‐MMR Nb‐IMDQ treatment and provide a rational basis for its use in the targeted re‐education of pro‐tumoral MMR^high^ TAMs in vivo.

## 4 Conclusion

Imidazoquinolines like IMDQ show a great potential to reprogram pro‐tumoral TAMs into a pro‐inflammatory state, however, due to their pharmacokinetic profile they cause unfavorable systemic inflammation. To overcome this issue, we developed a novel methodology to site‐specifically and quantitatively couple IMDQ (and other molecules, such as fluorescent dyes) to TAM targeting Nbs directed against MMR. By in vitro and in vivo studies, we confirmed that this modification did not interfere with the superior binding of these Nbs to their target. Anti‐MMR Nb‐IMDQ conjugates led to an efficient drug delivery to the MMR^high^ TAMs in vivo, inducing a repolarization of TAMs towards a pro‐inflammatory phenotype and an increase in anti‐tumor T‐cell responses, which cause a significant decline in tumor growth. In summary, our findings prove the therapeutic benefit of the anti‐MMR Nb‐IMDQ treatment and provide a rational basis for its use in the targeted re‐education of pro‐tumoral MMR^high^ TAMs in vivo.

## Conflict of Interest

The authors declare no conflict of interest.

## Supporting information

Supporting Information

## Data Availability

Data available in article supplementary material or on request from the authors.
