# Supplementary material for: Targeted Repolarization of Tumor‐Associated Macrophages via Imidazoquinoline‐Linked Nanobodies
Source: Adv Sci (Weinh). 2021 Mar 8;8(10):2004574. doi: 10.1002/advs.202004574 (PMC8132149; doi:10.1002/advs.202004574)
Supplement: Supplementary file 1 — Supporting Information [file ADVS-8-2004574-s001.pdf]

© 2021 Wiley-VCH GmbH

ADVANCED SCIENCE
Open Access

Supporting Information

for *Adv. Sci.,* DOI: 10.1002/advs.202004574

Targeted Repolarization of Tumor-associated Macrophages via Imidazoquinoline-linked Nanobodies

Evangelia Bolli,[+] Maximilian Scherger,[+] Sana M. Arnouk,[+] Ana Rita Pombo Antunes, David Straßburger, Moritz Urschbach, Judith Stickdorn, Karen De Vlaminck, Kiavash Movahedi, Hans Joachim Räder, Sophie Hernot, Pol Besenius, Jo A. Van Ginderachter, and Lutz Nuhn**

Supporting Information

Targeted Repolarization of Tumor-associated Macrophages via Imidazoquinoline-linked Nanobodies

Evangelia Bolli,[+] Maximilian Scherger,[+] Sana M. Arnouk,[+] Ana Rita Pombo Antunes, David Straßburger, Moritz Urschbach, Judith Stickdorn, Karen De Vlaminck, Kiavash Movahedi, Hans Joachim Räder, Sophie Hernot, Pol Besenius, Jo A. Van Ginderachter, and Lutz Nuhn**

Dr. E. Bolli,[+] S. M. Arnouk,[+] A. R. Pombo Antunues, K. De Vlaminck, Dr. K. Movahedi, Prof. J. A. Van Ginderachter
Lab of Cellular and Molecular Immunology, Vrije Universiteit Brussel, Pleinlaan 2, 1050 Brussels, Belgium
Myeloid Cell Immunology Lab, VIB Center for Inflammation Research, 1050 Brussels, Belgium
E-mail: jo.van.ginderachter@vub.be

M. Scherger,[+] J. Stickdorn, Dr. H. J. Räder, Dr. L. Nuhn
Max Planck Institute for Polymer Research, Ackermannweg 10, 55128 Mainz, Germany
E-mail: lutz.nuhn@mpip-mainz.mpg.de

Dr. D. Straßburger, M. Urschbach, Prof. P. Besenius
Department of Chemistry, Johannes Gutenberg-University Mainz, Duesbergweg 10–14, 55128 Mainz, Germany

Prof. S. Hernot
Laboratory of In Vivo Cellular and Molecular Imaging, Vrije Universiteit Brussel, Laarbeeklaan 103, 1090 Brussels (Belgium)

[+]: equal contribution.

*: corresponding authors: Prof. Dr. J. A. Van Ginderachter (jo.van.ginderachter@vub.be) and Dr. L. Nuhn (lutz.nuhn@mpip-mainz.mpg.de)

MATERIALS AND METHODS

Materials

Unless stated otherwise, all solvents and reagents were obtained from commercial sources in at least *pro analysi* (p.a.) quality and were used without further purification. The absolutation of solvents was performed according to standard laboratory procedures (e.g. W. L. Armarego, D. Perrin, *Purification of Laboratory Chemicals*, 1997). Ultrapure water was obtained from an *Elga PURELAB flex 4* system by *Veolia* (Paris, France). All protein modifications were performed in DPBS buffer supplemented with DMSO, both purchased from Sigma Aldrich. Fluorescent dyes Alexa Fluor™ 488 C_5-Maleimide and NIRdye 800CW-Maleimide were purchased from ThermoFisher Scientific and LI-COR Biosciences, respectively. TCEP (Tris(2-carboxyethyl) phosphine hydrochloride) and ABA (4-azidobenzoic acid) were purchased from TCI Chemicals and Na_2EDTA from Sigma Aldrich. Bio-Gel P-30 Gel for SEC was purchased from Bio-Rad Laboratories, Inc., Novex Sharp. Pre-stained Protein Standard (3.5–260 kDa) was purchased from ThermoFisher Scientific and Coomassie Brilliant Blue R-250 was purchased from NeoLab Migge. For concentrating protein solutions Amicon Ultra-4 3k centrifugal filter units with regenerated cellulose from Merck Millipore were used.

Reaction conditions

Air-and moisture-sensitive reactions were carried out under an argon atmosphere with common *Schlenk* techniques and dry solvents. For this purpose, all glassware were previously oven-dried or dried via heat gun *in vacuo* at least three times. In addition, all necessary solvents and liquid starting materials were added *via* septum and with disposable syringes, which were previously flushed with argon. Solids were added under continuous counterflow of argon.

Chromatography

All flash-chromatographic purifications were carried out using silica gel with an average particle size of 35–70 µm and pore size of 60 Å by *Acros Organics* (Geel, Belgium). A nitrogen pressure of 0.3–0.5 bar was applied. The eluents were freshly prepared of *pro analysi* grade solvents or distilled technical grade solvents. The analysis of the collected fractions was performed via TLC.

The TLC-analyses were carried out on silica-coated aluminum sheets 60 F^{254} by *Merck KGaA* (Darmstadt, Germany) with fluorescence-indicator. The analytes were detected by the following methods: UV-absorption at a wavelength of 254 nm; Vanillin-stain (solution prepared of 100 mL methanol with 1.0 g vanillin, 12 mL acetic acid and 4mL conc. sulfuric acid); Ninhydrin-stain (solution prepared of 1.5 g ninhydrin in 500 mL methanol and 15 mL acetic acid); $KMnO_4$-stain (solution prepared of 6 g $KMnO_4$, 40 g K_2CO_3 and 13 mg NaOH in 600 mL H_2O. In case of staining agents, the plate is immersed shortly and the color reaction subsequently proceeds upon heating.

NMR spectroscopy

All ^{1}H and ^{13}C NMR-spectra were recorded on a Bruker Avance II 400 spectrometer equipped with a 5 mm broadband observe probehead (z-gradient) using standard Bruker release pulse sequences (^{1}H NMR: 400 MHz, ^{13}C NMR: 100.7 MHz) or a Bruker Avance III 600 spectrometer equipped with a 5 mm cryogenic triple-band inverse probehead (z-gradient) using standard Bruker release pulse sequences (^{1}H NMR: 600 MHz, ^{13}C NMR: 150.9 MHz) both by *Bruker* (Rheinstätten, Germany). The samples were dissolved in deuterated solvents obtained from *Deutero* (Kastellaun, Germany). The chemical shifts (δ/ppm) are referenced to the residual proton signal of the deuterated solvent and reported in parts per million (ppm) relative to tetramethylsilane (δ = 0.00 ppm). Coupling constants (J) are calculated in Hertz (Hz) and the spin multiplicity of the signals is reported using following abbreviations: bs = broad singlet, s = singlet, d = doublet, t = triplet, q = quartet and m = multiplet, as well as appropriate combinations of these. The following abbreviations for substructures are used in the course of proton assignments: Q = quinoline, XDA = xylylenediamine, IQ = imidazoquinoline, MI = maleimide, Bu = butyl. The spectra were processed and analysed using MestReNova 14.1.0 by *MESTRELAB RESEARCH* (Santiago de Compostela, Spain).

SDS-PAGE

The SDS-PAGE was performed using a 16% polyacrylamide gel with a thickness of 0.5 mm and stained with Coomassie Brilliant Blue R-250. As marker, 7.5 µL of Novex Sharp Pre-stained Protein Standard (3.5–260 kDa) were used. 10 µL protein solution containing 2 µg of protein (native and modified Nbs, respectively) was incubated for 30 minutes with 3.33 µL of 4-fold concentrated Lämmli-buffer (0.2 M Tris-Cl pH 6.8,

8% SDS, 0.4% Bromophenol blue, 40% glycerol) and then loaded to the gel. Electrophoresis was performed at 160 V for 1 hour before visualization by UV-vis irradiation (for monitoring fluorescent dyes) as well as by Coomassie staining.

UV-vis spectroscopy

The UV-vis spectra were recorded at 20°C with a Jasco V-630 spectrophotometer and a JASCO ETC-717 peltier thermostatted cell holder tempered via a water thermostat A. Krüss Optronic V50.

Mass spectrometry

High resolution electrospray ionization mass spectra (ESI-HRMS) were recorded on an Agilent 6545 Q-TOF mass spectrometer (*Agilent Technologies*, Santa Clara, USA) with LockSpray interface. Alternatively, ionization using field desorption was performed on a MAT 95 by *Finnigan* (San Jose, USA).

Mass spectrometry (MS) experiments for protein analyses were performed on a SYNAPT G2-Si instrument (Waters Corp., Manchester, UK) equipped with an ESI source. The instrument was calibrated by clusters of sodium formate in the mass range 50–2,000 *m*/*z*. Nb measurements were carried out at a capillary voltage of 3 kV, a sampling cone voltage of 80 V, a source offset of 80 V and a source temperature of 100 °C. Data processing was done with MassLynx software V4.2 (Waters GmbH, Eschborn, Germany, 2005). The deconvolution data were obtained from multiple charged signals at charge species from 7^+ to 15^+ using the MaxEnt software. All chemicals for MS measurements and sample preparation were used in liquid chromatography (LC)/MS quality. For a typical run, 30 µg of each Nb sample in PBS was desalted using Pierce C-18 spin columns (ThermoFisher Scientific) following the manufacturer's protocol including additional five washing steps with 5% acetonitrile and 0.5% formic acid in water. Each Nb could be recovered in acetonitrile/water (7:3 vol.) with 0.1% formic acid and infused at a flow rate of 5 µL/min by a syringe pump (Legato 180 from Kd Scientific, Holliston, MA, USA) into the mass spectrometer.

Mice, cell line and tumor models

Female C57BL/6 mice were purchased from Janvier. MMR-deficient (MMR-KO) C57BL/6 mice were provided by Etienne Pays (Université Libre de Bruxelles). Mice

were injected subcutaneously (s.c.) in the flank with $3x10^6$ Lewis Lung Carcinoma cells expressing the chicken ovalbumin (LLC-OVA) or 3LL-R cells. The 3LL-R clone of the C57BL/6 LLC cell line was derived as described previously[3]. Procedures followed the guidelines of the Belgian Council for Laboratory Animal Science (approval number: 15-220-07).

Tumor and PEM preparation

Tumors grown in WT or MMR-KO mice were minced and treated with 10 U/mL collagenase I, 400 U/mL collagenase IV, and 30 U/mL DNase I (Worthington) for 30 minutes. For *in vitro* tumor cell suspension incubation with fluorescently-labeled Nbs and for TAM sorting from 3LL-R tumors, a step of density gradient (Axis-Shield) was used to remove debris and dead cells. The cells were collected, resuspended in FACS buffer (HBSS containing 0.5% FCS and 2 mM EDTA) and counted. PEMs were obtained by rinsing the peritoneum of naive mice with PBS. For preparation of cDNA, PEMs were FACS sorted to high purity. To purify PEMs, peritoneal exudate cells were sorted from 6-10 naive mice and gated as $CD11b^+Ly6C^{low}F4/80^+$ cells.

Cell sorting (MACS, FACS)

3LL-R and LLC-OVA tumors were grown in WT C57Bl/6 mice, tumor single cell suspensions were prepared as described earlier. $CD11b^+$ cells were selected via MACS, using anti-CD11b microbeads following the manufacturer's protocol (Miltenyi Biotec). The $CD11b^+$ fraction was then sorted to purify the $MHC\text{-}II^{low}$ TAMs using a BD FACSAriaTM II (BD Biosciences). $MHC\text{-}II^{low}$ TAMs were gated as described in **supplementary Figures S1 and S2**. The fluorescently-labeled antibodies used for cell sorting are described later.

***In vitro* incubation of sorted TAMs with IMDQ, anti-MMR Nb or anti-MMR Nb-IMDQ**

TAMs were sorted from 3LL-R tumors grown s.c. in WT C57BL/6 mice as described earlier. Afterwards, TAMs were incubated with the specified concentration of IMDQ, anti-MMR Nb-IMDQ, anti-MMR Nb or with PBS as a control, for 48 hours at 37°C. The culture supernatant and the cells were collected separately to measure the concentration of several secreted molecule and gene expression levels, respectively.

RNA extraction, cDNA preparation and qRT-PCR

These experiments were performed as previously described[4]. RNA was extracted using TRIzol (Invitrogen), RNeasy Mini Kit or RNeasy Micro Kit (QIAGEN) when cell number was higher than $1x10^6$, between $0.5x10^6$ and $1x10^6$ or lower than $0.5x10^6$, respectively. Afterwards, RNA was reverse-transcribed with oligo(dT) and SuperScript II RT (Invitrogen). qRT-PCR was performed in an iCycler, with iQ SYBR Green Supermix (Bio-Rad). Primer sequences are listed in **Supplementary Table 1**. PCR cycles consisted of 1' 94°C, 45'' 55°C, 1' 72°C. Gene expression was normalized using ribosomal protein S12 (*Mrps12*) as a housekeeping gene.

Measurement of NO, TNF-α, IL-6 and IL-1β concentrations

NO was quantified by a Griess reaction as described previously[5]. TNF-α, IL-6 and IL-1β concentrations were measured by ELISA, according to the supplier's protocols (R&D systems).

Generation of anti-MMR Nbs, BCII10 Nb and bivalent MMR Nb

The generation, selection and characterization of the anti-mouse MMR-specific Nb clone 1, bivalent anti-MMR Nb[1] and BCII10 Nb[2] were described previously. The Nbs were recloned into the vector pHEN25 to encode a C-terminal His6 tag and Cys tag. Periplasmic expression and purification of the Nbs was carried out as described previously[1,2]. Due to C-terminal oxidized Cys, dimerized Nb fractions were also isolated and used for this study.

Alexa Fluor™ 647-Maleimide labeling of BCII10 Nb

Different parameters were first optimized to achieve optimal nanobody modification by a model fluorescent dye. Best results were obtained under the following conditions: To a BCII10 Nb solution (5 µg, 0.332 nmol, 1.35 mg/mL) 3.3 µL of 10 mM stock solution of TCEP in DPBS (9.52 µg, 33.2 nmol) were added and shaken for 1 hour at room temperature for Nb C-terminal Cys reduction. Excess of TCEP was then quenched by adding 4.2 µL of a 10 mM ABA stock solution in DPBS (6.8 µg, 41.50 nmol) and shaken for 30 minutes at room temperature. 1.5 µL of a 5 mg/mL stock solution of Alexa Fluor™ 647 C2 Maleimide in DMSO (7.5 µg, 6.0 nmol) were added and the reaction

mixture was allowed to incubate overnight at 4 °C. During all reaction steps, the level of Na_2EDTA was held at 10 mM using a 100 mM stock solution in DPBS (14.0 µL in total). All aqueous stock solutions were freshly prepared and adjusted to a pH value of 7.4 with 1 M NaOH. The reaction mixtures could directly be analyzed by SDS-PAGE.

NIRdye 800CW-Maleimide labeling of α-MMR Nb

To an α-MMR Nb solution (500 µg, 33.2 nmol, 1.35 mg/mL) 332 µL of 10 mM stock solution of TCEP in DPBS (952 µg, 3.320 mmol) were added and shaken for 1 hour at room temperature for Nb C-terminal Cys reduction. Excess of TCEP was then quenched by adding 415 µL of a 10 mM ABA stock solution in DPBS (677 µg, 4.150 mmol) and shaken for 30 minutes at room temperature. 142.4 µL of a 5 mg/mL stock solution of NIRdye 800CW-Maleimide in DMSO (712 µg, 0.598 mmol) were added and the reaction mixture was allowed to incubate overnight at 4 °C. During all reaction steps, the level of Na_2EDTA was held at 10 mM using a 100 mM stock solution in DPBS (140 µL in total). All aqueous stock solutions were freshly prepared and adjusted to a pH value of 7.4 with 1 M NaOH. The reaction mixture was added onto a Bio-Gel P-30 size exclusion column to separate free dye and other small molecules from the Nb sample. The modified protein was eluted and isolated with azide-free DPBS and quantified via UV-vis spectroscopy.

NIRdye 800CW-Maleimide labeling of BCII10 Nb

The BCII10 Nb was labeled with NIRdye 800CW maleimide in analogy to the procedure of the α-MMR Nb.

Alexa Fluor™ 488 C_5-Maleimide labeling of α-MMR and BCII10 Nbs

The labeling with AF488 was done in analogy to the IR-dye labeling as described above.

Site-selective modification of anti-MMR-Nb with IMDQ-PEG_4-Maleimide

anti-MMR Nb was modified with IMDQ-PEG_4-Maleimide in analogy to the procedure described above for Nb labeling with dyes. Instead of a 100 mM Na_2EDTA stock solution, only a 10 mM stock solution was used to prevent precipitation during the reaction caused by higher amounts of DMSO. After quenching with ABA, 20

equivalents of IMDQ-PEG$_4$-Maleimide (in relation to reactive thiol groups) were dissolved in DMSO and added to the reaction mixture with additional amounts of DMSO to increase the DMSO content to 10%. After 2 hours of shaking at room temperature, the precipitated unreacted IMDQ-derivate was separated by centrifugation, dissolved in DMSO and added again to the reaction mixture reaching a final amount of 15% DMSO. The reaction mixture was allowed to incubate overnight at 4 °C. Unreacted IMDQ-derivate was then separated again by centrifugation and the remaining solution was diluted to a final amount of 5% DMSO. Using Amicon Ultra-4 3k centrifugal filter units, the protein solutions were concentrated for subsequent purification via SEC (Bio-Gel P-30). The obtained fractions were analyzed by UV-vis spectroscopy to determine protein (absorbance at 280 nm) and IMDQ (absorbance at 322 nm) concentration.

Site-selective modification of BCII10 Nb with IMDQ-PEG4-Maleimide

The BCII10 Nb was modified with IMDQ-PEG4-Maleimide in analogy to the procedure of the anti-MMR Nb described above.

IMDQ and IMDQ-maleimide TLR7/8 receptor activity

RAW-Blue macrophages from Invivogen, Toulouse (France) were used for TLR stimulation. An embryonic alkaline phosphatase was chromosomally integrated into this cell line and can be secreted upon TLR7/8 stimulation via NF-kB and AP-1 signaling. The secreted enzyme can be quantified spectrophotometrically as indicator for immune stimulation. Raw Blue macrophages were cultured in Dulbecco's modified Eagle's medium (DMEM) supplemented with 10% fetal bovine serum, 2 mm l-glutamine, 1 mm sodium pyruvate, 1% penicillin/ streptomycin, 0.01% normocin and 0.02% zeocin as selection medium. Cells were kept at 37°C with 5% CO2 saturation. Cells were seeded into 96-well plates at a density of 90000 cells/well in 180 µL culture medium. Each well was treated with 20 µL of IMDQ sample at the given final concentrations. After incubation for 18 h, 50 µL of the supernatant from each well was collected and tested for secreted embryonic alkaline phosphatase (SEAP) using the QUANTI-Blue assay (InvivoGen). 150 µL QUANTI-Blue was added to each sample and incubated at 37 °C. SEAP levels were determined by measuring optical density at 615 nm using a microplate reader. Activity was determined by an increase in optical density relative to the negative control treated with PBS. All experiments were

conducted at n=4. Moreover, cell viability could be verified by MTT assay simultaneously. 50 µL of 3-(4,5-dimethylthiazol-2-yl)-2,5-diphenyltetrazolium bromide (MTT) (0.5 mg/mL in PBS) was then added to the RAW-Blue cells. After an incubation period of 2–3 h, the formed formazan crystals were dissolved by addition of 100 µL 10% m/v SDS/0.01 m HCl and incubated overnight at 37 °C. Quantification was done by measuring the absorbance at 570 nm using a microplate reader.

***In vitro* incubation of tumor cell suspensions with anti-MMR Nb-AF488 and BCII10 Nb-AF488**

Tumor single cell suspensions from 14-day old 3LL-R tumors grown s.c. in WT and MMR-KO C57BL/6 mice were prepared as described earlier. Then, the cells were incubated with the specified concentration of anti-MMR Nb-AF488 and BCII10 Nb-AF488 for 2 hours at 4°C or 37°C. Afterwards, the cells were collected, FACS stained (as described later) and the AF488 staining was analyzed within the different myeloid subset according to the gating strategy shown in **Supplementary figure S1**.

NIR fluorescence imaging

To study the biodistribution of the modified Nbs, anti-MMR Nb and BCII10 Nb were site-specifically labeled with NIRdye800CW as described. 2 nmol of Nb-NIRdye (concentration based on fluorophore concentration which also corresponds to the Nb concentration, Nb/dye ratio=1) were injected with or without a 5-fold molar excess of bivalent unlabeled anti-MMR Nb intravenously in LLC-OVA-bearing WT or MMR-KO C57Bl/6 mice. Reflectance images of the dorsal and ventral side of the mice were acquired over 21 hours with the Fluobeam800 (Fluoptics) (exposure time 200 and 300 ms). Fluorescence images were overlaid with white light images for anatomical localization. 21 hours p.i, tissue/organs were isolated and imaged *ex vivo* (exposure time 50-300ms). Using ImageJ, regions of interest (ROI) were drawn and mean fluorescence signal was measured within these ROIs. Attention should be drawn to the fact that quantification of NIRdye signal is restricted and can only be used for indicative comparison of different conditions within the same tissue/organ and not between different organs, as normalization of signal to tissue/organ weight was not possible to realize.

^{99m}Tc-anti-MMR Nb-IMDQ labeling, pinhole SPECT-μCT imaging, biodistribution analysis

To study the *in vivo* biodistribution of anti-MMR Nb-IMDQ and BCII10 Nb-IMDQ, both conjugates were labeled with ^{99m}Tc at their hexahistidine tail and quality assured, as described before[1,6,7]. LLC-OVA-bearing WT or MMR KO C57Bl/6 mice were intravenously (i.v.) injected with 59±9 MBq of ^{99m}Tc-labeled Nb conjugates in 100 μl, with or without a 5-fold molar excess of bivalent unlabeled anti-MMR Nb. 180 minutes p.i, animals were anesthetized with 2% isoflurane and SPECT/μCT-imaging was performed using the VECTor (MILabs). Image viewing was performed using AMIDE Medical Image Data Examiner software. After the 15-minute scan, animals were sacrificed, and organs were collected and weighed. Radioactivity was measured using an automated γ-counter (Cobra II Inspector 5003; Canberra-Packard). Tissue and organ uptake were calculated as percentage of injected activity per gram tissue (%IA/g), corrected for decay.

FACS staining

Fc receptors were blocked using anti-mouse CD16/32 mAb generated in house (clone 2.4G2) for 15 minutes at 4°C. After washing with HBSS, samples were stained with fixable viability dye eFluor506 (eBioscience, Live/dead stain) (1/1000) for 30 minutes at 4 °C in the dark. Subsequently, samples were washed and stained with the following commercial antibodies used for cell surface staining: anti-CD11b(M1/70)/PE-Cy7 (BioLegend), anti-Ly6G (1A8)/PerCP-Cy5.5 (Tonbo Biosciences), anti-Ly6C(ER-MP20)/APC (Serotec), anti-SiglecF (E50-2440)/PE (BD-Biosciences), anti-MHC-II (M5/114.15.2)/Brilliant Violet 421 (BioLegend), anti-CD45 (30-F11)/APC-Cy7 (BioLegend), anti-F4/80 (MCA497Q488)/AF488 (Bio-rad), anti-CD19 (6D5)/ Brilliant Violet 421 (BioLegend), anti-TCRβ (H57-597)/APC (Biolegend), anti-NK1.1 (PK136)/PE (eBioscience), anti-CD4 (GK1.5)/ PerCP/Cy5.5 (Biolegend), anti-CD8a (53-6.7)/ Brilliant Violet 421 (Biolegend) anti-Granzyme B (GB11)/FITC (Biolegend), anti-CD62L(MEL-14)/PE (eBioscience), anti-CD44 (IM7)/FITC (Biolegend).

FACS data were acquired using a BD FACSCanto II (BD Biosciences) and analyzed using FlowJo (Tree Star, Inc.).

Statistical analysis

Evaluation of outliers was carried out by two-sided Grubbs' test (alpha value = 0.05). Data is always shown as mean ± standard error of the mean (SEM). To compare two independent groups with normally distributed data, two-tailed, unpaired Student's t-test was used (alpha value = 0.05). Comparisons including two independent variables (i.e., comparing tumor growth curves whereby tumor volumes and day of measurement are considered) were done by two-way ANOVA (alpha value = 0.05). In all cases, significance was defined as *: $p \leq 0.05$; **: $p \leq 0.01$; ***: $p \leq 0.001$; ****: $p \leq 0.0001$. Statistical analysis was carried out using GraphPad Prism v7.00 (GraphPad Software, La Jolla, California, United States of America).

Supplementary data

Gating strategy

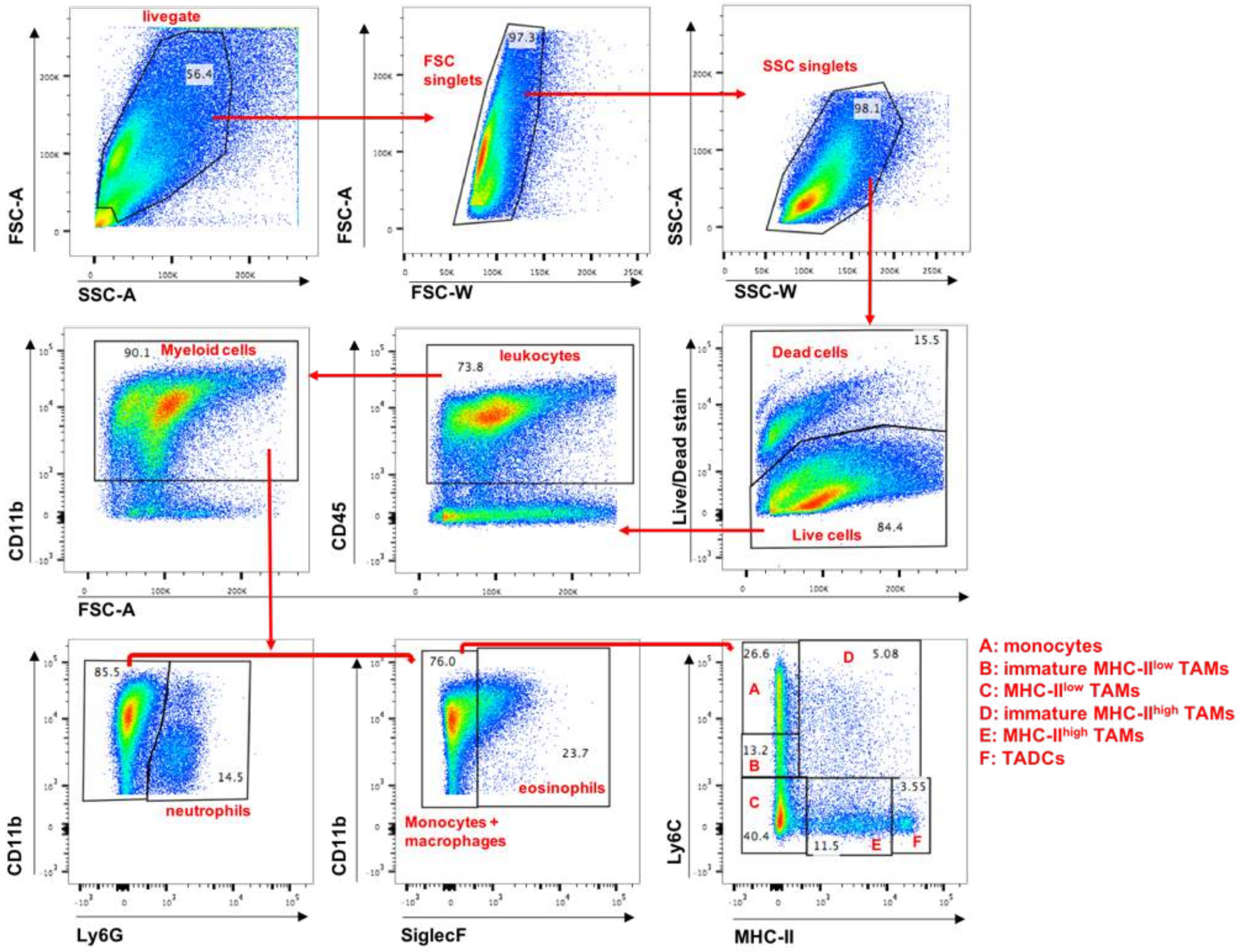

Figure S1: Gating strategy for the different myeloid subsets and for sorting MHC-II^{low} TAMs from 3LL-R tumors in FACS. Primarily, the cell suspension was plotted on forward and side scatter in order to gate the live cells (livegate) and single cells according to both forward (FSC singlets) and side (SSC singlets) scatters. Live cells were again selected according to fixable viability dye which stains dead cells. Then, different immune myeloid subsets were gated as: CD45$^+$CD11b$^+$Ly6G$^+$ for neutrophils, CD45$^+$CD11b$^+$Ly6G$^-$ SiglecF$^+$ for eosinophils, CD45$^+$CD11b$^+$Ly6G$^-$ SiglecF$^-$Ly6C^{high} for monocytes (A), CD45$^+$CD11b$^+$Ly6G$^-$ SiglecF$^-$Ly6C^{intermediate}MHC-II$^{low/high}$ for immature MHC-II$^{low/high}$ TAMs (B/D), CD45$^+$CD11b$^+$Ly6G$^-$SiglecF$^-$ Ly6C^{low}MHC-II^{low} for MHC-II^{low} TAMs (C), CD45$^+$CD11b$^+$Ly6G$^-$SiglecF$^-$Ly6C^{low}MHC-II^{high} for MHC-II^{high} TAMs (E), CD45$^+$CD11b$^+$Ly6G$^-$SiglecF$^-$Ly6C^{low}MHC-II$^{very\ high}$ for TADCs (F).

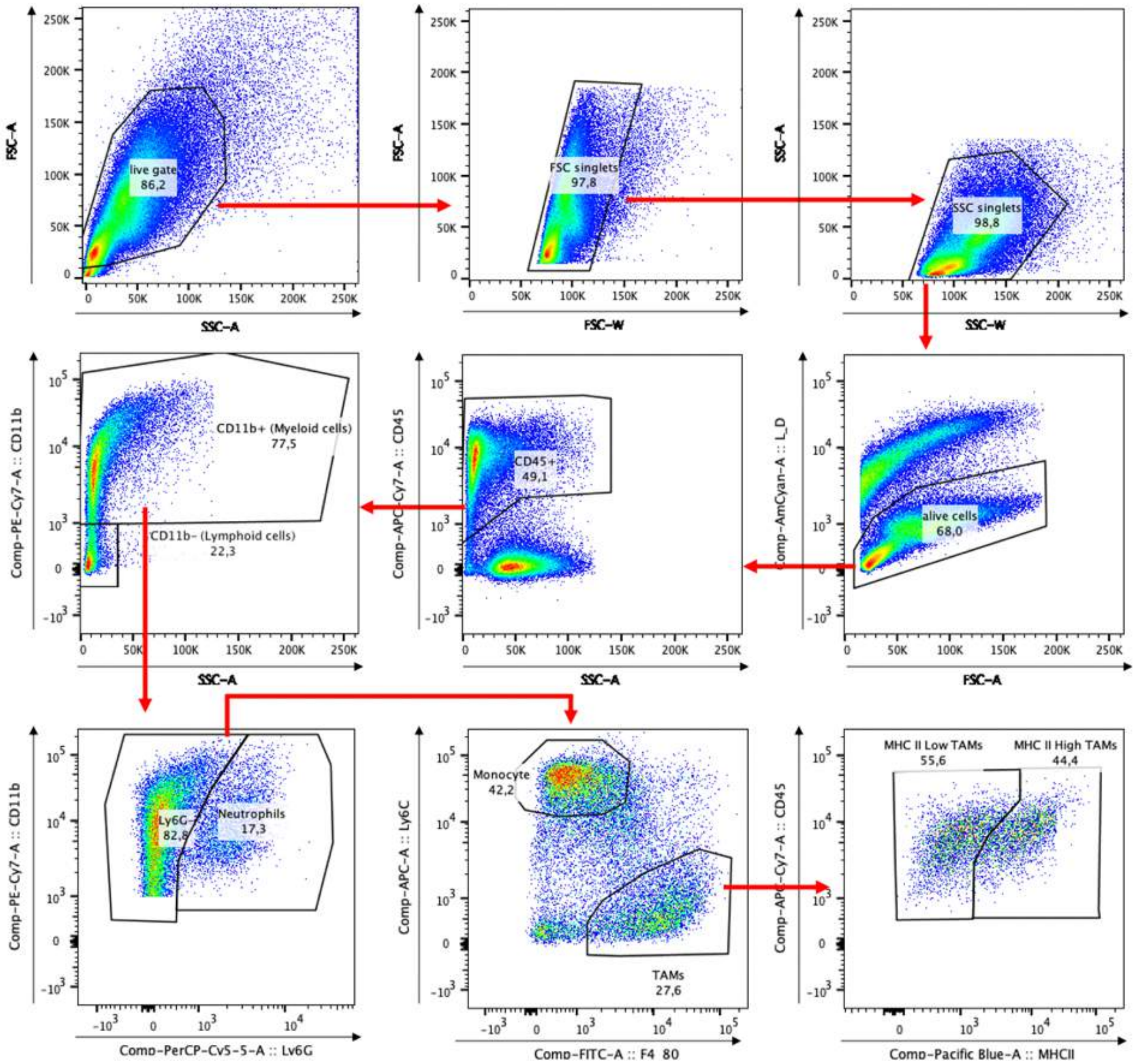

Figure S2: Gating strategy for sorting MHC-II^{low} TAMs from LLC-OVA tumors in FACS. Primarily, the cell suspension was plotted on forward and side scatter in order to gate the live cells (livegate) and single cells according to both forward (FSC singlets) and side (SSC singlets) scatters. Live cells were selected according to fixable viability dye which stains dead cells. Then, different immune myeloid subsets were gated as: CD45$^+$CD11b$^+$Ly6G$^+$ for neutrophils, CD45$^+$CD11b$^+$Ly6G$^-$Ly6C^{High}F4/80^{Low} for monocytes and CD45$^+$CD11b$^+$Ly6G$^-$Ly6C^{low}F4/80^{High} for TAMs which were further subdivided into MHC II^{Low} and MHC II^{High} TAMs.

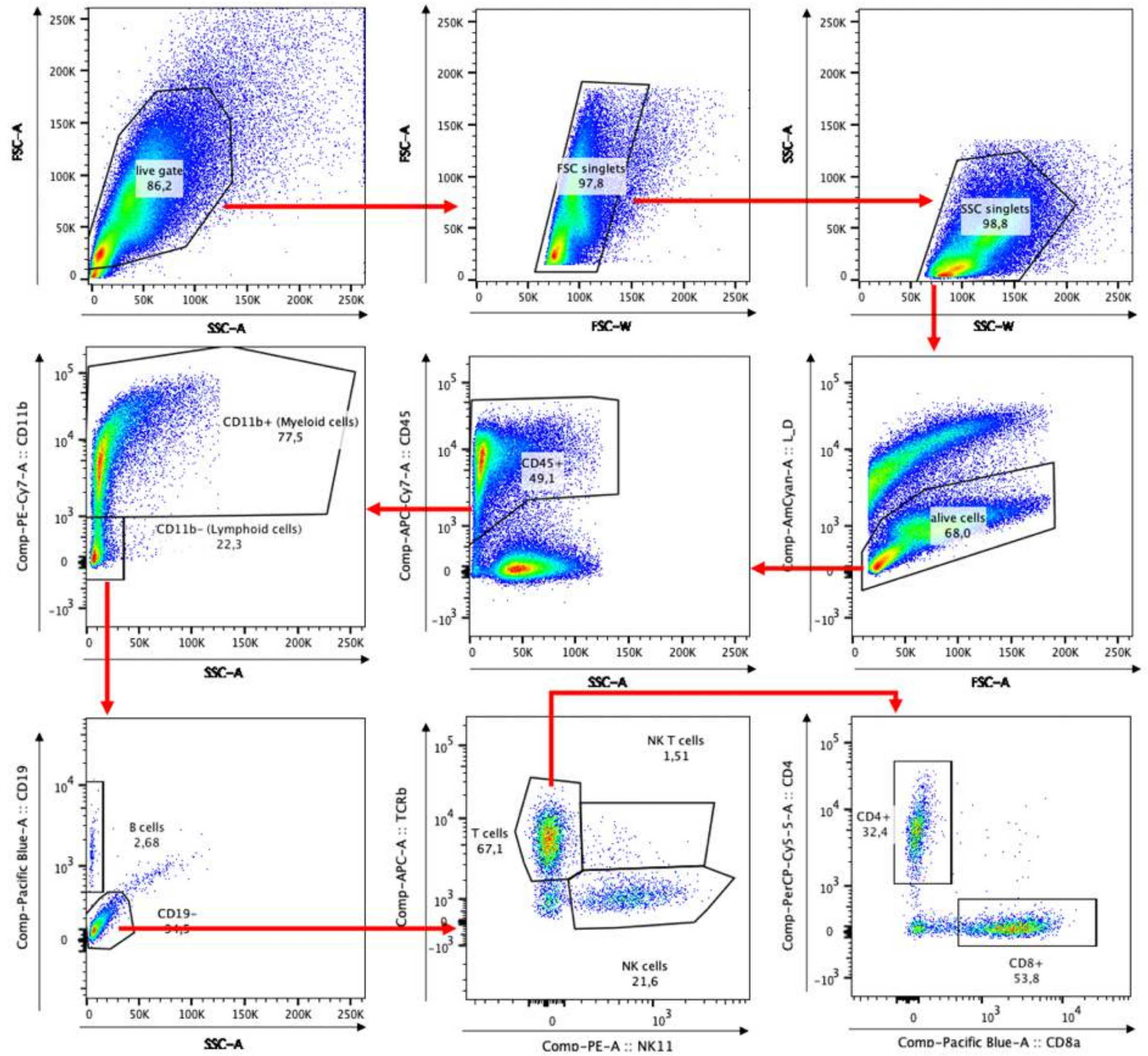

Figure S3: Gating strategy for the different lymphoid subsets infiltrating LLC-OVA tumors in FACS. Primarily, the cell suspension was plotted on forward and side scatter in order to gate the live cells (livegate) and single cells according to both forward (FSC singlets) and side (SSC singlets) scatters. Live cells were selected according to fixable viability dye which stains dead cells. Then, different immune lymphoid subsets were gated as: $CD45^+CD11b^-CD19^+$ for B cells, $CD45^+CD11b^-CD19^-NK1.1^+TCR\beta^-$ *for NK cells,* $CD45^+CD11b^-CD19^-NK1.1^+TCR\beta^+$ for NK T cells and $CD45^+CD11b^-CD19^-NK1.1^-TCR\beta^+$ for T cells which were further subdivided into $CD4^+$ and $CD8^+$ T cells.

Non-modified nanobody characterization

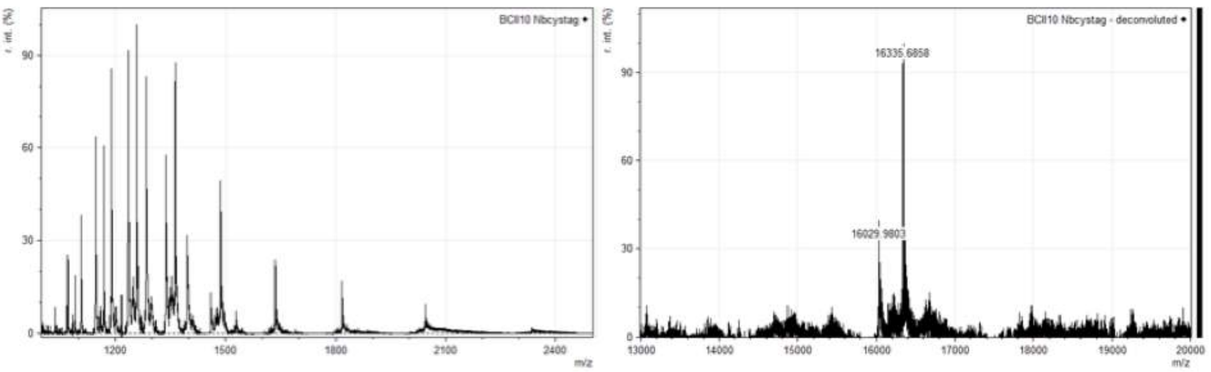

Figure S4: ESI-Q-TOF-MS profile of isolated monomeric BCII10 Nb with C-terminal cysteine tag (left: recorded spectrum – right: deconvoluted). Note that BCII10 Nb (~16032.84 Da, found: 16029.98 Da) isolated as monomeric species has formed a disulfide with glutathione (307.33 g·mol^{-1}) during the nanobody production and isolation (cf. 16335.69 Da). Molecular weights were calculated by ExPASy web portal.

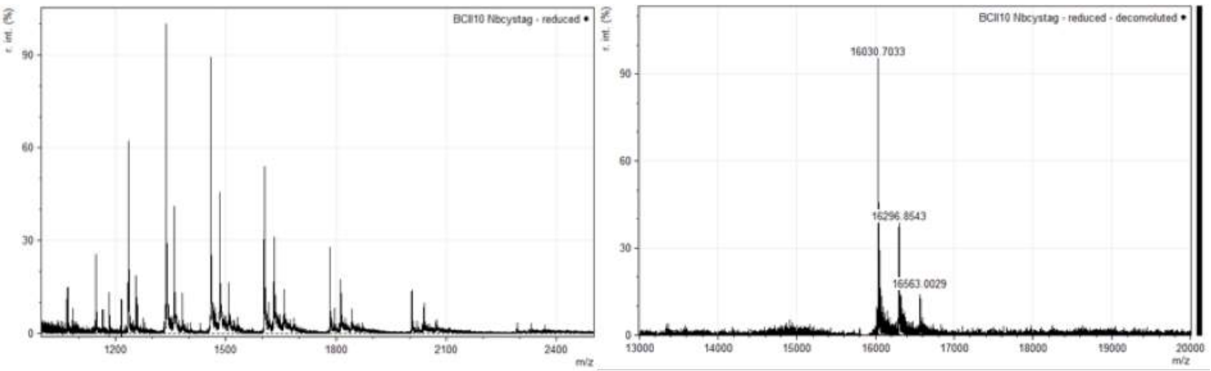

Figure S5: ESI-Q-TOF-MS profile of BCII10 Nb C-terminal cysteine tag after reduction with TCEP (left: recorded spectrum – right: deconvoluted). The monomer BCII10 Nb (~16032.84 Da, found: 16030.70 Da) forms only some minor adducts with the phosphine oxide (266.185 g·mol^{-1}) during the measurement (cf. 16296.85 Da and 16563.00 Da). Molecular weights were calculated by ExPASy web portal.

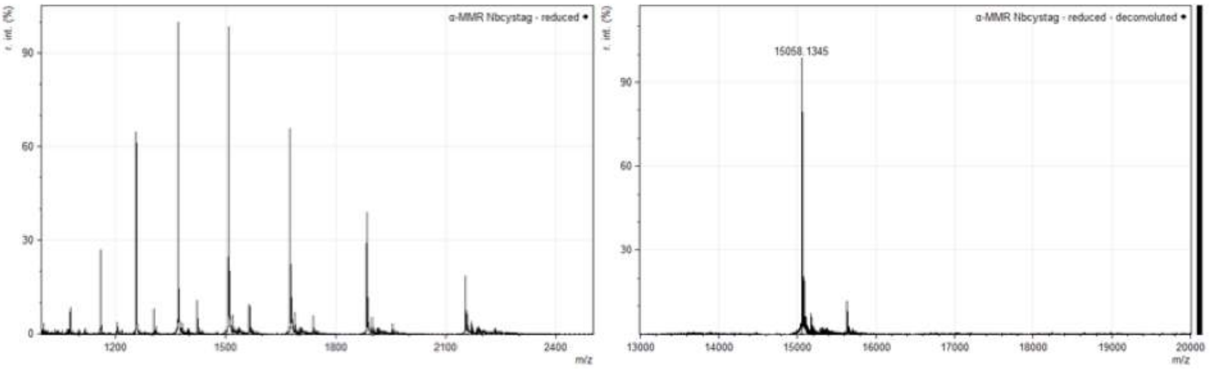

Figure S6: ESI-Q-TOF-MS profile of α-MMR C-terminal cysteine tag (~15059.53 Da, found: 15058.13 Da) after reduction with TCEP (left: recorded spectrum – right: deconvoluted). Molecular weights were calculated by ExPASy web portal.

Modified nanobody characterization

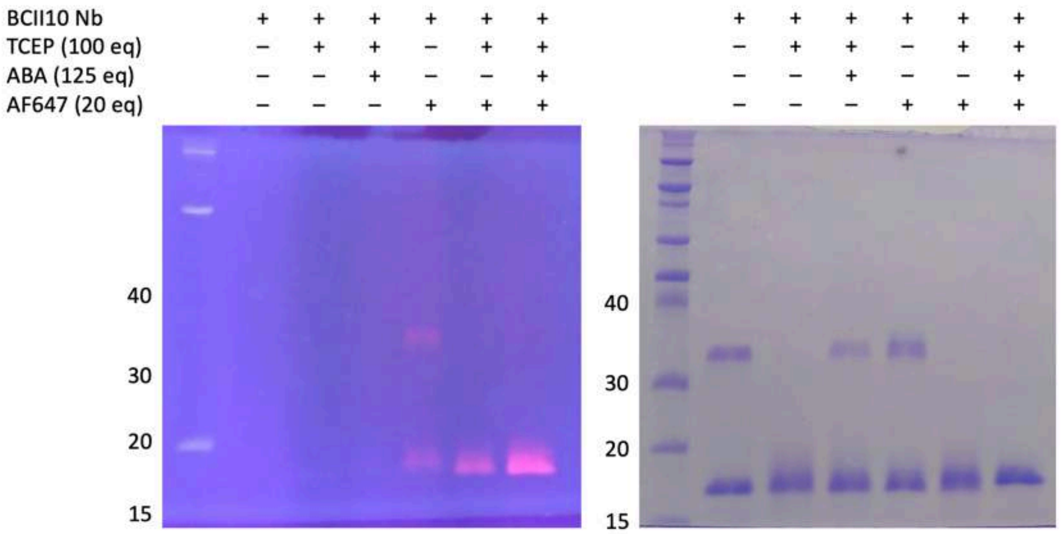

Figure S7: SDS-PAGE image of various reaction conditions for AF647 labelling of BCII10 Nb with UV-vis irradiation (left panel) and coomassie staining (right panel). BCII10 Nb dimer (~32065.7 Da) is reduced by TCEP to BCII10 Nb monomer (~16032.8 Da). Unreacted TCEP is quenched with ABA, resulting in a more efficient labelling with AF647 (cf. intensities of penultimate and last lanes, left panel). Molecular weights were calculated by ExPASy web portal.

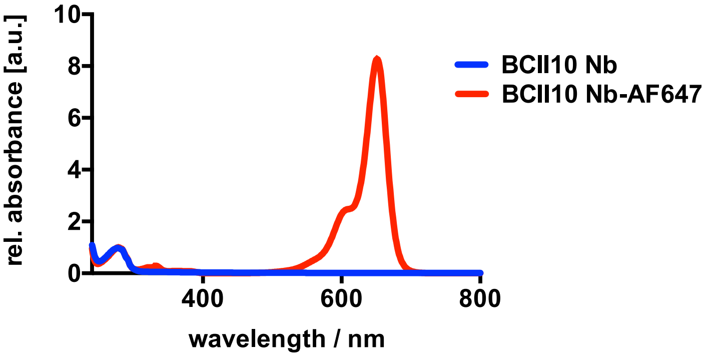

Figure S8: UV-vis spectrum of AF647-maleimide labelled BCII10 Nb. Degree of labelling (DL) determined by absorbance maxima at 650 nm and 280 nm (extinction coefficients provided by the customer):

$$DL = \frac{A_{650nm}}{A_{280nm}} \cdot \frac{\varepsilon_{Nb,280nm} + \varepsilon_{dye,280nm}}{\varepsilon_{dye,650nm}} = \frac{A_{650nm}}{A_{280nm}} \cdot \frac{\varepsilon_{dye,280nm} + 0.03 \cdot \varepsilon_{dye,650nm}}{\varepsilon_{dye,650nm}} = \frac{8.238}{0.983} \cdot \frac{24535 + 717}{239000} \approx 1$$

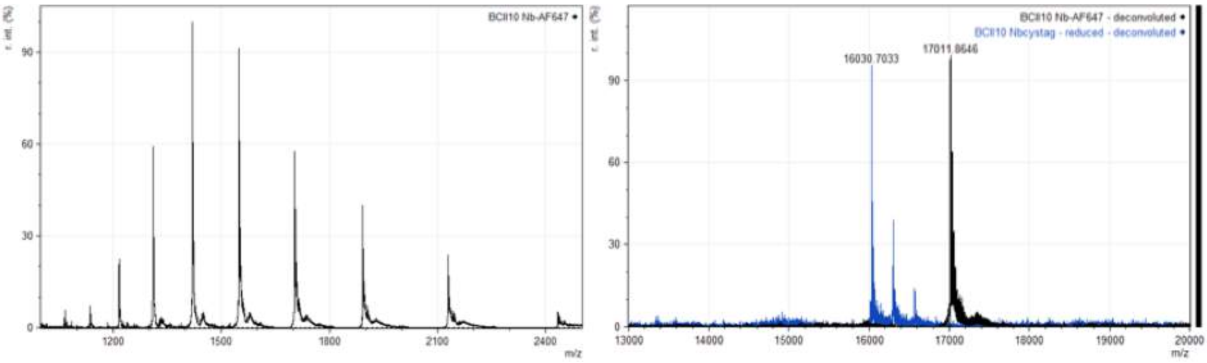

Figure S9: ESI-Q-TOF-MS profile of AF647 maleimide labelled BCII10 Nb (left: recorded spectrum – right: deconvoluted). The shift in molecular weight from 16030.7 Da (BCII10 Nb, blue) to 17011.9 Da (BCII10 Nb-AF647, black) indicates a successful mono-functionalization of the nanobody.

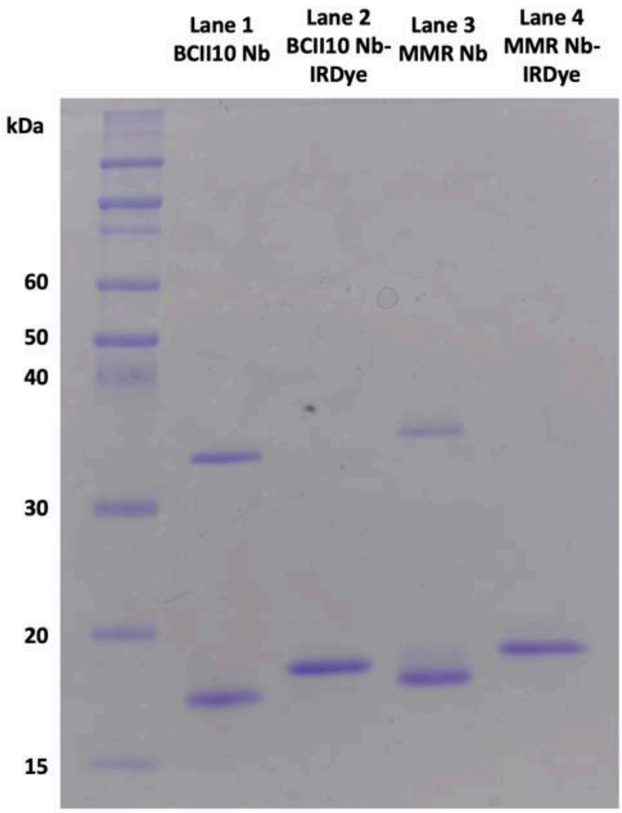

Figure S10: SDS-PAGE image stained with Coomassie Blue dye for native α-MMR Nb (monomer= ~15059.5 Da, dimer= ~30120.0 Da; lane 3), α-MMR Nb-NIRdye 800CW (= ~16184.8 Da; lane 4), native BCII10 Nb (monomer= ~16032.8 Da, dimer= ~32065.7 Da; lane 1) and BCII10 Nb-NIRdye 800CW (= ~17158.1 Da; lane 2). Molecular weights were calculated by ExPASy web portal.

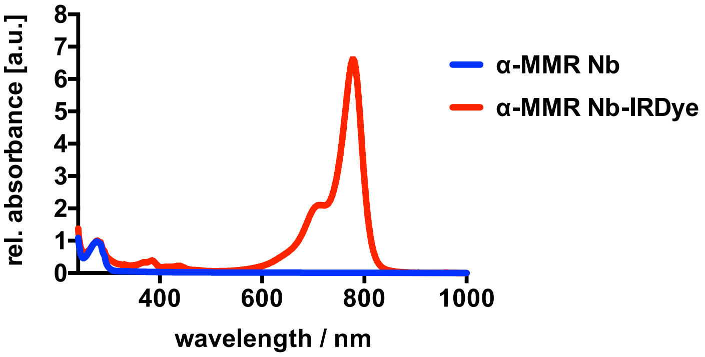

Figure S11: UV-vis spectrum of NIRdye 800CW-maleimide labelled α-MMR Nb. Degree of labelling (DL) determined by absorbance maxima at 650 nm and 280 nm (extinction coefficients provided by the customer):

$$DL = \frac{A_{774\,nm}}{A_{280\,nm}} \cdot \frac{\varepsilon_{Nb,280\,nm} + \varepsilon_{dye,280\,nm}}{\varepsilon_{dye,774\,nm}} = \frac{A_{774\,nm}}{A_{280\,nm}} \cdot \frac{\varepsilon_{Nb,280\,nm} + 0.03 \cdot \varepsilon_{dye,774\,nm}}{\varepsilon_{dye,774\,nm}} = \frac{6.645}{0.986} \cdot \frac{23045 + 7200}{240000} \approx 1$$

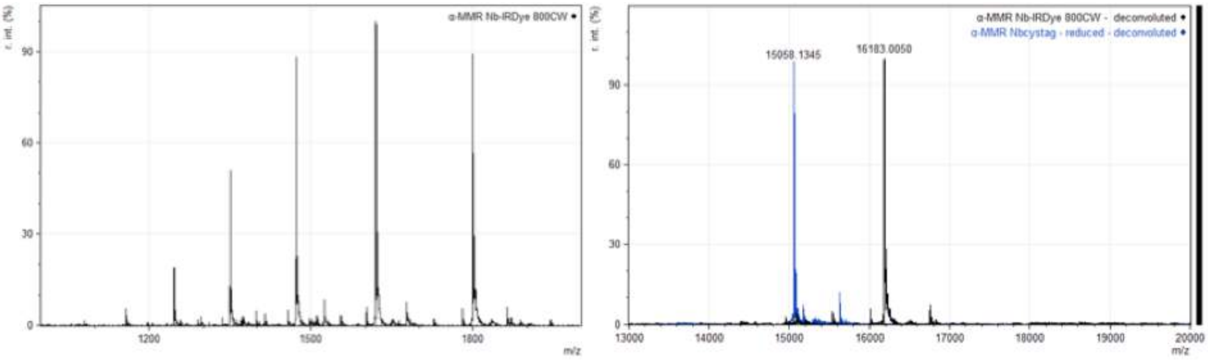

Figure S12: ESI-Q-TOF-MS profile of NIRdye 800CW maleimide labelled α-MMR Nb (left: recorded spectrum – right: deconvoluted). The shift in molecular weight from 15058.1 Da (α-MMR Nb; ~15059.5 Da; blue) to 16183.0 Da (α-MMR Nb-NIRdye 800CW; ~16184.8 Da; black) indicates a successful mono-functionalization of the nanobody with NIRdye 800CW maleimide (1191.3 g·mol^{-1}). Molecular weights were calculated by ExPASy web portal.

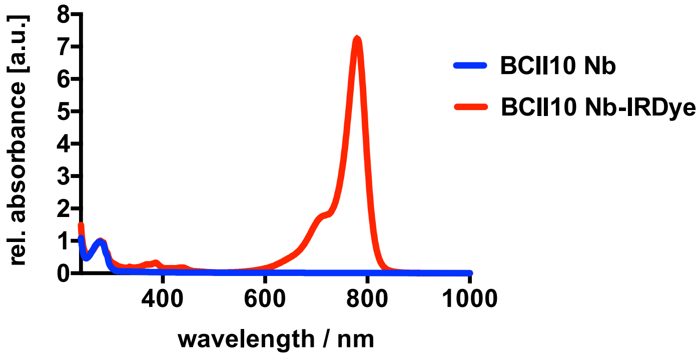

Figure S13: UV-vis spectrum of NIRdye CW600-maleimide labelled BCII10 Nb. Degree of labelling (DL) determined by absorbance maxima at 650 nm and 280 nm (extinction coefficients provided by the customer):

$$DL = \frac{A_{774nm}}{A_{280nm}} \cdot \frac{\varepsilon_{Nb,280nm} + \varepsilon_{dye,280nm}}{\varepsilon_{dye,774nm}} = \frac{A_{774nm}}{A_{280nm}} \cdot \frac{\varepsilon_{Nb,280nm} + 0.03 \cdot \varepsilon_{dye,774nm}}{\varepsilon_{dye,774nm}} = \frac{6.806}{0.987} \cdot \frac{24535 + 7200}{240000} \approx 1$$

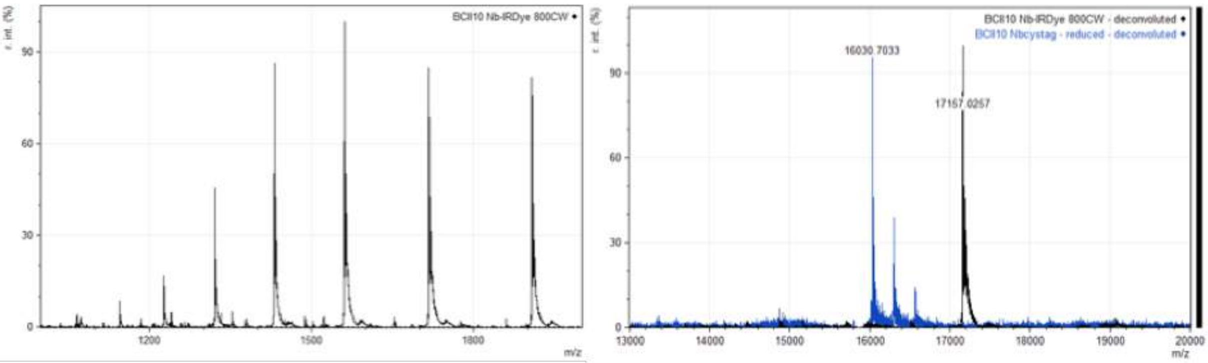

Figure S14: ESI-Q-TOF-MS profile of NIRdye 800CW maleimide labelled BCII10 Nb (left: recorded spectrum – right: deconvoluted). The shift in molecular weight from 16030.7 Da (BCII10 Nb; ~16032.8 Da; blue) to 17157.0 Da (BCII10 Nb-NIRdye 800CW; ~17158.1 Da; black) indicates a successful mono-functionalization of the nanobody with NIRdye 800CW Maleimide (1191.3 g·mol^{-1}). Molecular weights were calculated by ExPASy web portal.

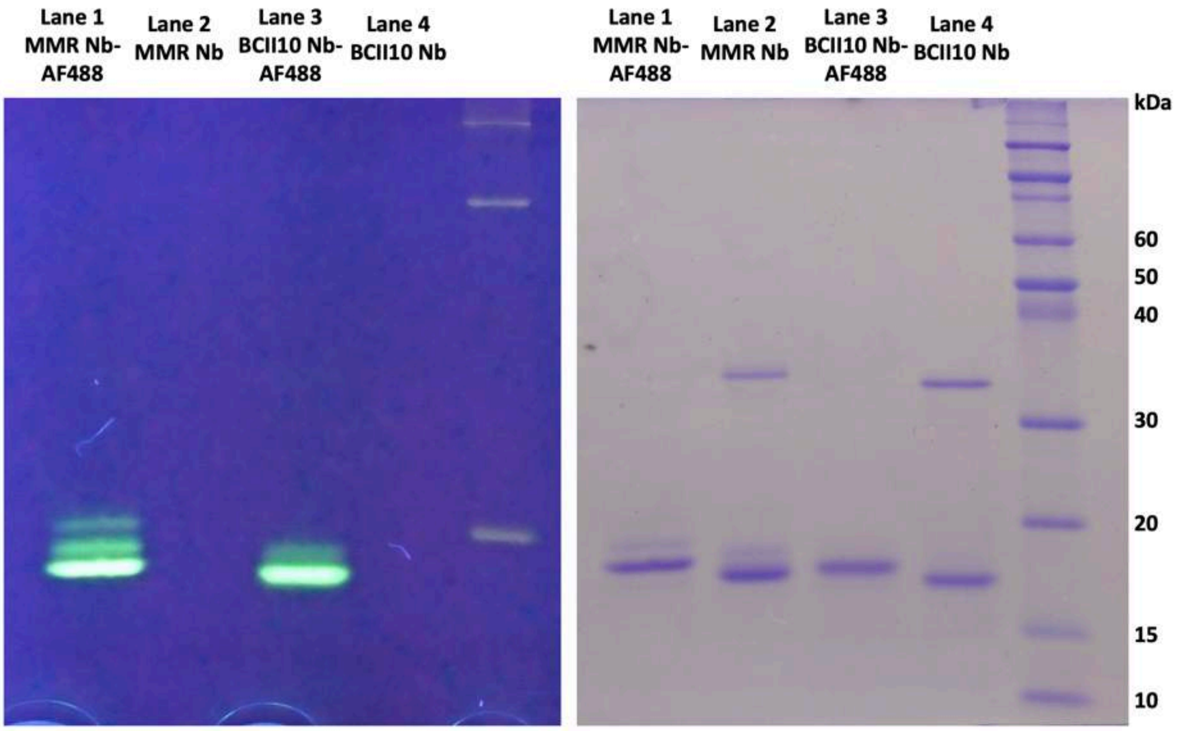

Figure S15: SDS-PAGE image recorded under fluorescent illumination (left) or stained with Coomassie Blue dye (right panel) for native α-MMR Nb (monomer= ~15059.5 Da, dimer= ~30119.6 Da; lane 2), α-MMR Nb-AF488 (= ~15775.6 Da; lane 1), native BCII10 Nb (monomer= ~16032.8 Da, dimer= ~32065.7 Da; lane 4) and BCII10 Nb-AF488 (= ~16749.0 Da; lane 3). Molecular weights were calculated by ExPASy web portal.

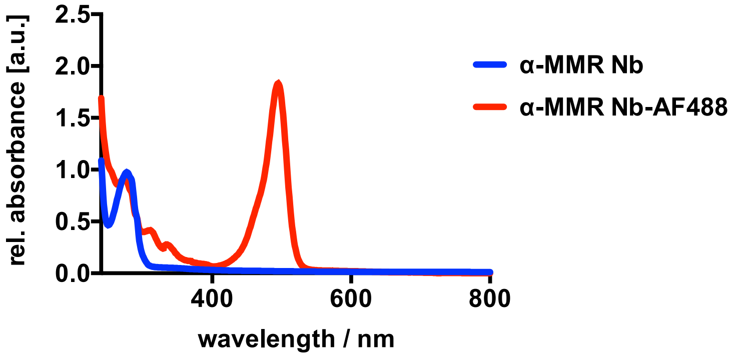

Figure S16: UV-vis spectrum of AF488 maleimide labelled α-MMR Nb. Degree of labelling (DL) determined by absorbance maxima at 496 nm and 280 nm (extinction coefficients provided by the customer):

$$DL = \frac{A_{496\,nm}}{A_{280\,nm}} \cdot \frac{\varepsilon_{Nb,280\,nm} + \varepsilon_{dye,280\,nm}}{\varepsilon_{dye,496\,nm}} = \frac{A_{496\,nm}}{A_{280\,nm}} \cdot \frac{\varepsilon_{Nb,280\,nm} + 0.11 \cdot \varepsilon_{dye,496\,nm}}{\varepsilon_{dye,496\,nm}} = \frac{1.823}{0.834} \cdot \frac{23045 + 7810}{71000} \approx 1$$

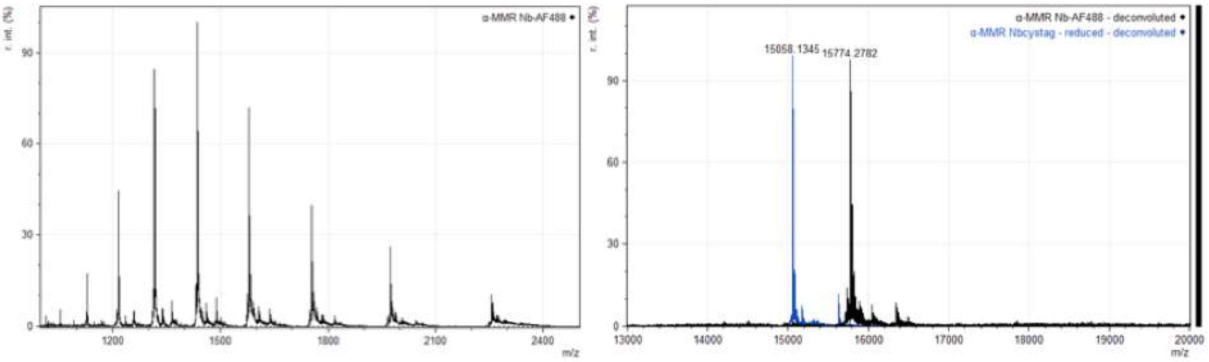

Figure S17: ESI-Q-TOF-MS profile of AF488 maleimide labelled α-MMR Nb (left: recorded spectrum – right: deconvoluted). The shift in molecular weight from 15058.1 Da (α-MMR Nb; ~15059.5 Da; blue) to 15774.3 Da (α-MMR Nb-AF488; ~15775.6 Da; black) indicates a successful mono-functionalization of the nanobody with AF488 maleimide (720.7 g·mol^{-1}). Molecular weights were calculated by ExPASy web portal.

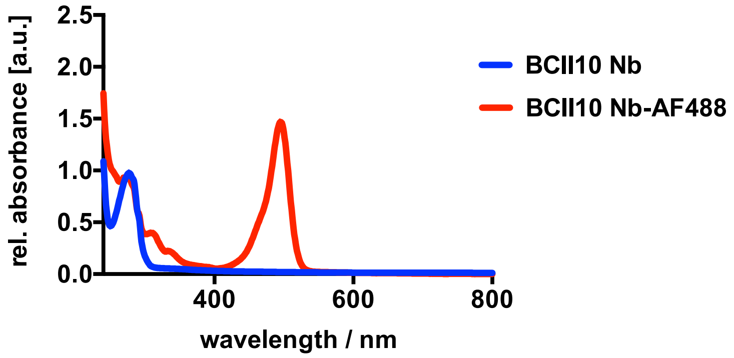

Figure S18: UV-vis spectrum of AF488 maleimide labelled BCII10 Nb. Degree of labelling (DL) determined by absorbance maxima at 496 nm and 280 nm (extinction coefficients provided by the customer):

$$DL = \frac{A_{496nm}}{A_{280nm}} \cdot \frac{\varepsilon_{Nb,280nm} + \varepsilon_{dye,280nm}}{\varepsilon_{dye,496nm}} = \frac{A_{496nm}}{A_{280nm}} \cdot \frac{\varepsilon_{Nb,280nm} + 0.11 \cdot \varepsilon_{dye,496nm}}{\varepsilon_{dye,496nm}} = \frac{1.468}{0.891} \cdot \frac{24535 + 7810}{71000} \approx 1$$

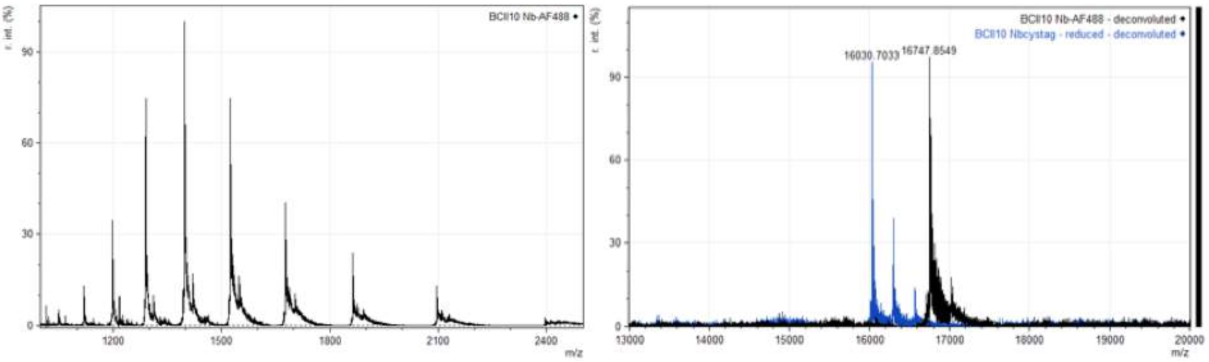

Figure S19: ESI-Q-TOF-MS profile of AF488 maleimide labelled BCII10 Nb (left: recorded spectrum – right: deconvoluted). The shift in molecular weight from 16030.7 Da (BCII10 Nbcystag; ~16032.8 Da; blue) to 16747.9 Da (BCII10 Nb-AF488; ~16749.0 Da; black) indicates a successful mono-functionalization of the nanobody with AF488 maleimide (720.7 g·mol^{-1}). Molecular weights were calculated by ExPASy web portal.

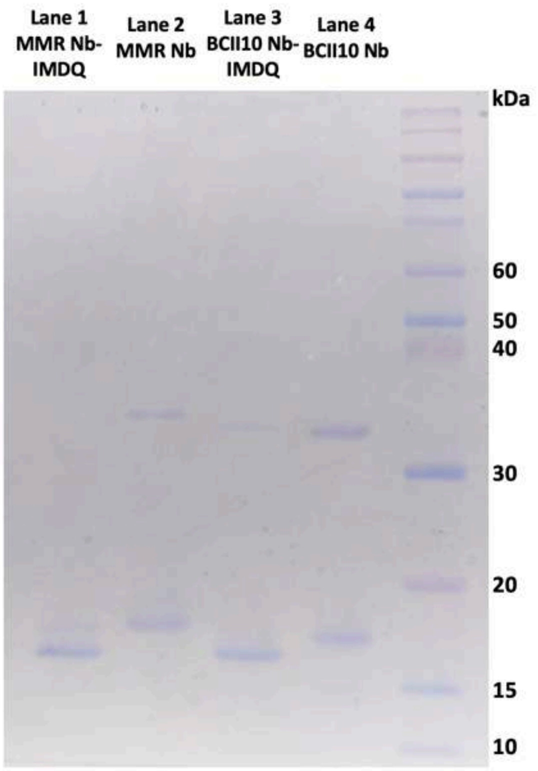

Figure S20: SDS-PAGE recorded after Coomassie Blue staining for α-MMR Nb (lane 2, monomer MW= ~15059.5 Da, dimer MW= ~30119.6 Da) and BCII10 Nb (lane 4, monomer MW= ~16032.8 Da, dimer MW= ~32065.7 Da) pre-modification as well as modified α-MMR Nb-IMDQ (lane 1, MW= ~15701.85 Da) and BCII10 Nb-IMDQ (lane 3, MW= ~16676.2 Da). Molecular weights were calculated by ExPASy web portal.

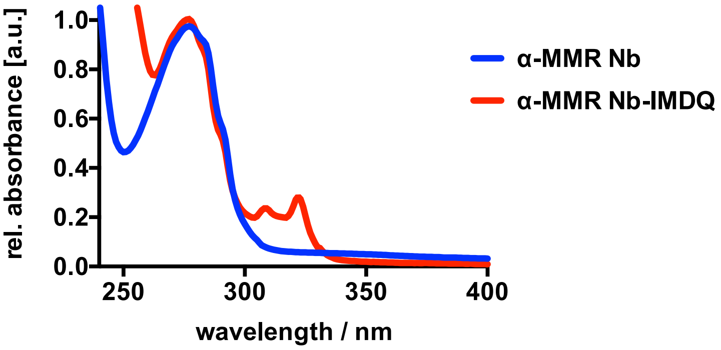

Figure S21: UV-vis spectrum of IMDQ maleimide modified α-MMR Nb. Degree of labelling (DL) determined by absorbance maxima at 322 nm and 280 nm (extinction coefficients derived from an IMDQ reported in L. Nuhn et al. *PNAS* **2016**, *113*, 8089-8103):

$$DL = \frac{A_{322\,nm}}{A_{280\,nm}} \cdot \frac{\varepsilon_{Nb,280\,nm} + \varepsilon_{IMDQ,280\,nm}}{\varepsilon_{IMDQ,322\,nm}} = \frac{0.225}{0.949} \cdot \frac{23045 + 4886}{5366} \approx 1$$

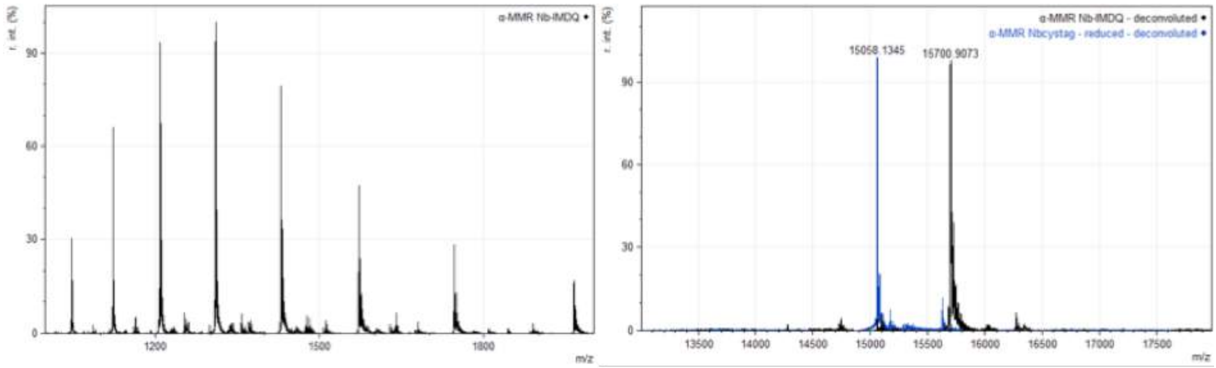

Figure S22: ESI-Q-TOF-MS profile of α-MMR Nb-IMDQ (left: recorded spectrum – right: deconvoluted). The shift in molecular weight from 15058.13 Da (α-MMR Nbcystag; ~15059.53 Da) to 15700.91 Da (α-MMR Nb-IMDQ; ~15701.85 Da) corresponds to the molecular weight of IMDQ-PEG$_4$-maleimide (642.7 g·mol^{-1}) and thus indicates a successful mono-functionalization of the nanobody. Molecular weights were calculated by ExPASy web portal.

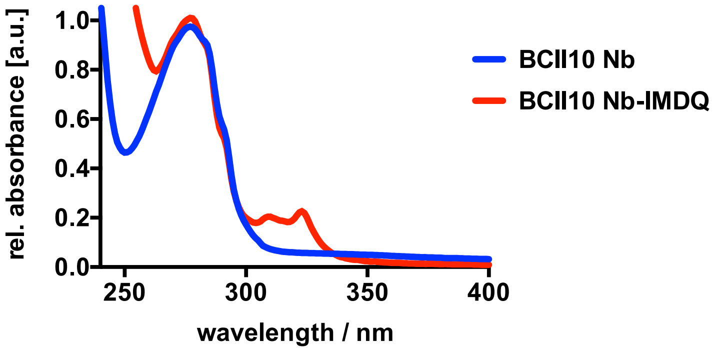

Figure 23: UV-vis spectrum of IMDQ maleimide modified BCII10 Nb. Degree of labelling (DL) determined by absorbance maxima at 322 nm and 280 nm (extinction coefficients derived from an IMDQ reported in L. Nuhn et al. *PNAS* **2016**, *113*, 8089-8103):

$$DL = \frac{A_{322nm}}{A_{280nm}} \cdot \frac{\varepsilon_{Nb,280nm} + \varepsilon_{IMDQ,280nm}}{\varepsilon_{IMDQ,322nm}} = \frac{0.164}{0.972} \cdot \frac{24535 + 4886}{5366} \approx 1$$

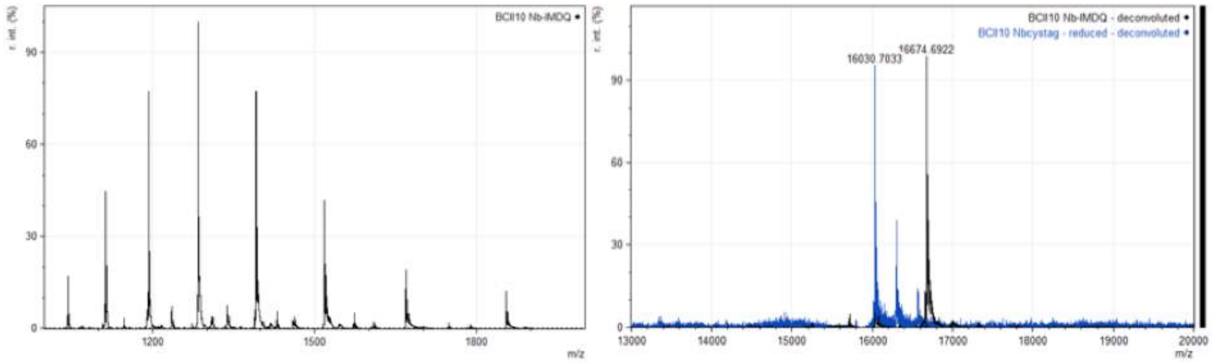

Figure S24: ESI-Q-TOF-MS profile of BCII10 Nb-IMDQ (left: recorded spectrum – right: deconvoluted). The shift in molecular weight from 16030.7 Da (BCII10 Nbcystag, blue) to 16674.7 Da (BCII10 Nb-IMDQ, black) corresponds to the molecular weight of IMDQ-PEG$_4$-maleimide (642.7 g·mol^{-1}) and thus indicates a successful mono-functionalization of the nanobody. Molecular weights were calculated by ExPASy web portal.

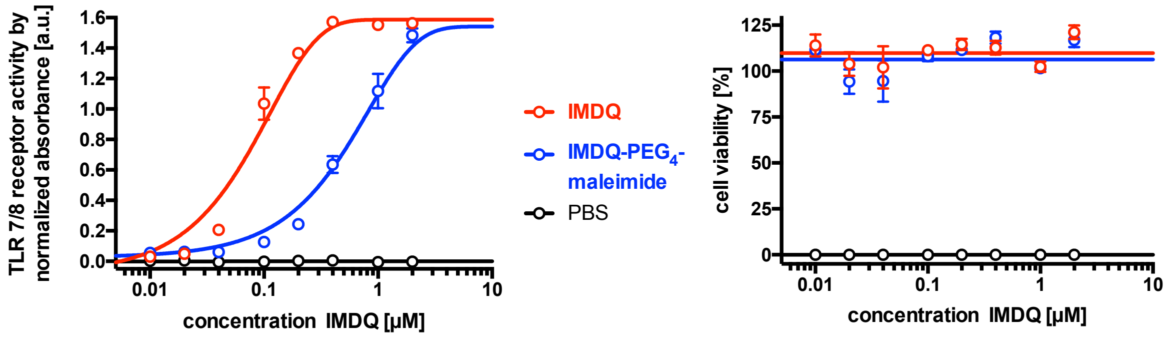

Figure S25: Results of RAW Blue assay (left) and cell viability (right) for IMDQ and IMDQ-PEG4-maleimide to determine the TLR7/8 receptor activity of the imidazoquinoline derivatives.

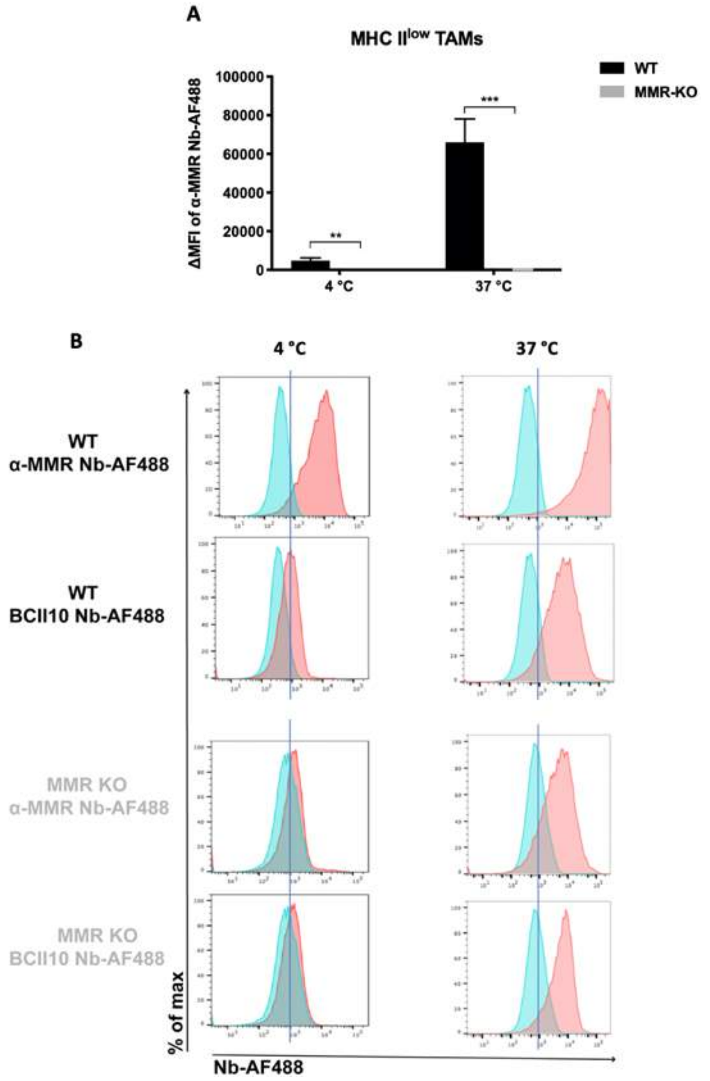

Figure S26: ***Ex vivo*** **uptake of modified anti (α)-MMR Nb by MHC-II^{low} TAMs.** Single cell suspensions from 14-day-old 3LL-R tumors grown in WT and MMR-KO C57Bl/6 mice were obtained and incubated with 10 µg/mL (~0.64 µM) of α-MMR Nb-AF488 or BCII10 Nb-AF488 for 2 hours at 4 °C and 37 °C, followed by FACS analysis. **A)** ΔMFI of α-MMR Nb-AF488 staining calculated as MFI of α-MMR Nb-AF488 – MFI of BCII10 Nb-AF488 and is shown for MHC II^{Low}MMR^{high} TAMs from tumors grown in WT and MMR-KO C57Bl/6 mice. Data is shown as mean ± SEM of 3 mice. Statistical analysis was achieved by an unpaired Student's t-test and is marked by **: p≤0,01; ***: p≤0,001. **B)** Representative histograms of Nb-AF488 staining (Red) of MHC-II^{low}MMR^{high} TAMs. Blue histograms represent background signal obtained when cells are incubated with PBS.

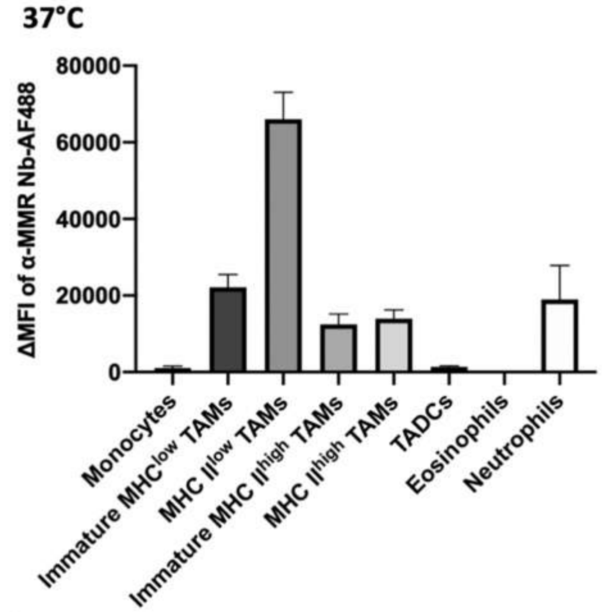

Figure S27: Conjugated anti (α)-MMR Nb targets MHC-II^{low}MMR^{high} TAMs. Single cell suspensions from 14 day-old 3LL-R tumors grown in WT C57Bl/6 mice were obtained and incubated with 10 μg/mL (~0.64 μM) of α-MMR Nb-AF488 or BCII10 Nb-AF488 for 2 hours at 37 °C, followed by FACS analysis. ΔMFI of α-MMR Nb-AF488 staining was calculated as MFI of α-MMR Nb-AF488 – MFI of BCII10 Nb-AF488 and is shown for several myeloid cell populations. The FACS gating strategy for the different myeloid cell populations is illustrated in Figure S1. TADCs: Tumor-associated dendritic cells.

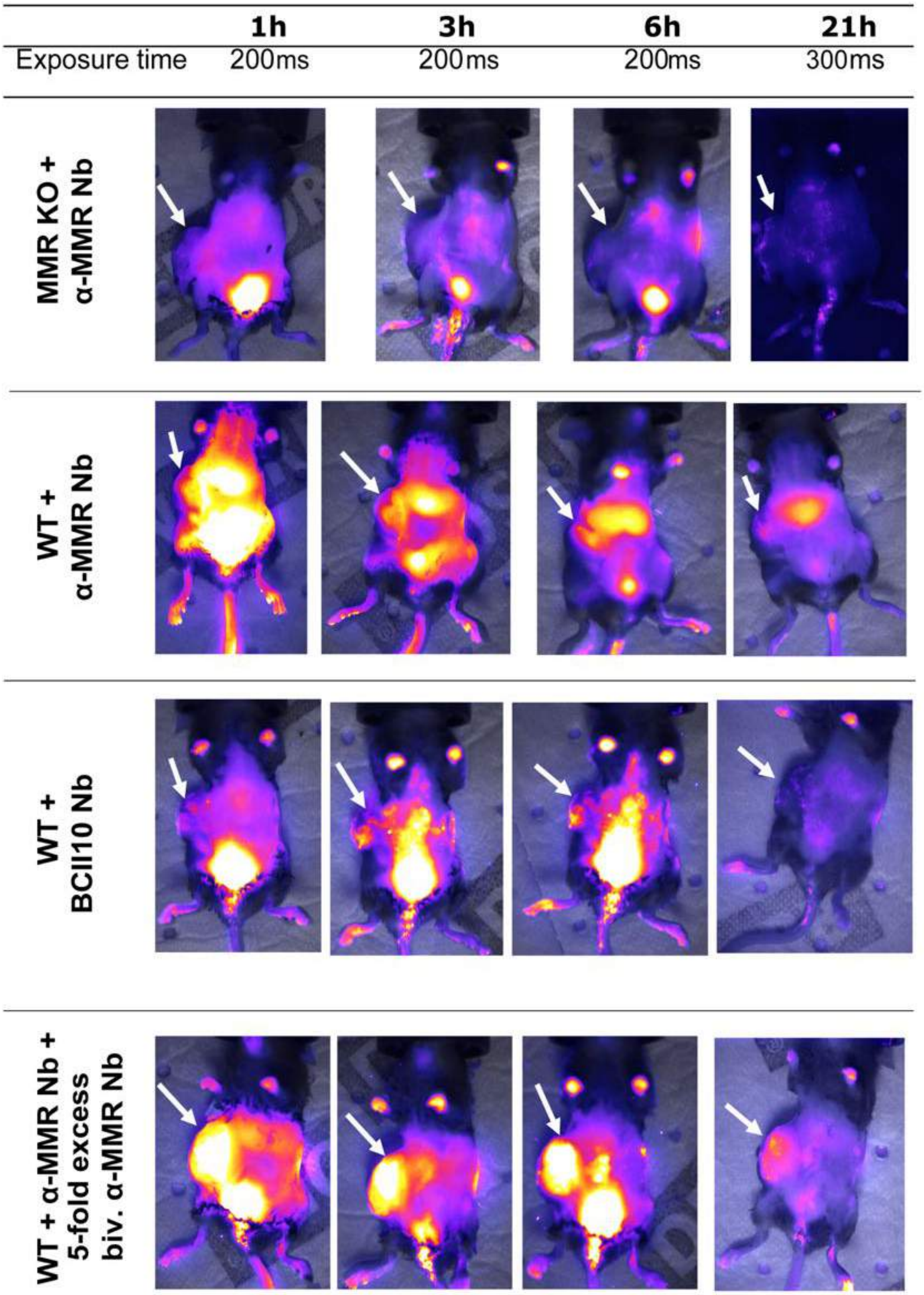

Figure S28: Biodistribution of anti (α)-MMR Nb-NIRDye800CW in LLC-OVA-bearing mice (I). 13-day-old LLC-OVA-bearing C57Bl/6 mice were injected intravenously with either 30μg α-MMR Nb-NIRdye 800CW (in WT and MMR KO mice), or 30μg BCII10 Nb-NIRdye 800CW as a control (in WT mice) or co-injected with 30μg α-MMR Nb-NIRdye 800CW and 300μg (5-fold molar excess) bivalent α-MMR Nb (in WT mice) and biodistribution was examined by NIR fluorescence imaging. Whole body imaging at 1 hour, 3 hours, 6 hours and 21 hours p.i.

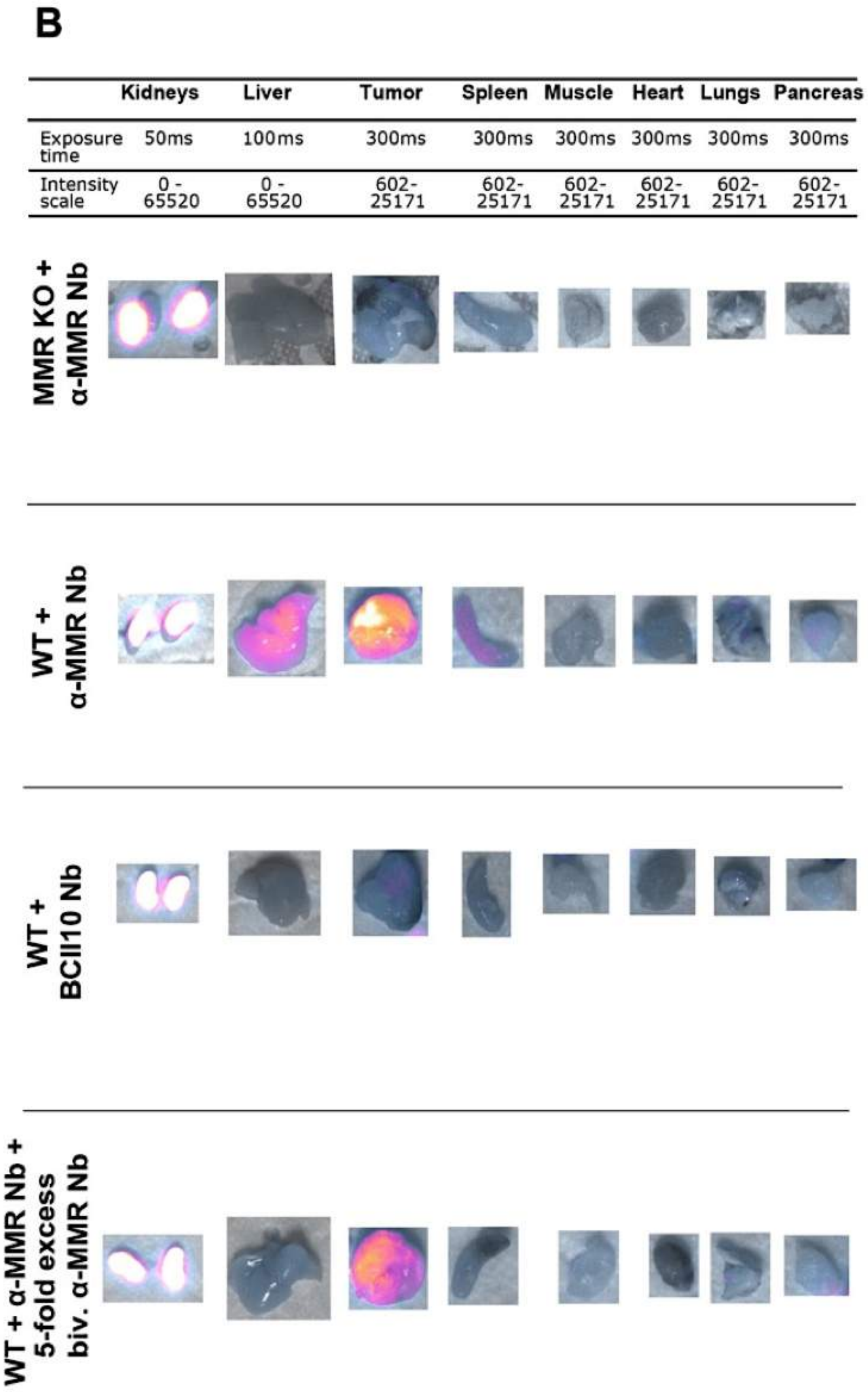

Figure S29: Biodistribution of anti (α)-MMR Nb-NIRdye 800CW in LLC-OVA-bearing mice (II). **I**maging of tumor, kidneys, liver, spleen, muscle, heart, lungs and pancreas isolated at 21 hours p.i. with the Fluobeam800 (Fluoptics) (exposure time 50-300 ms).

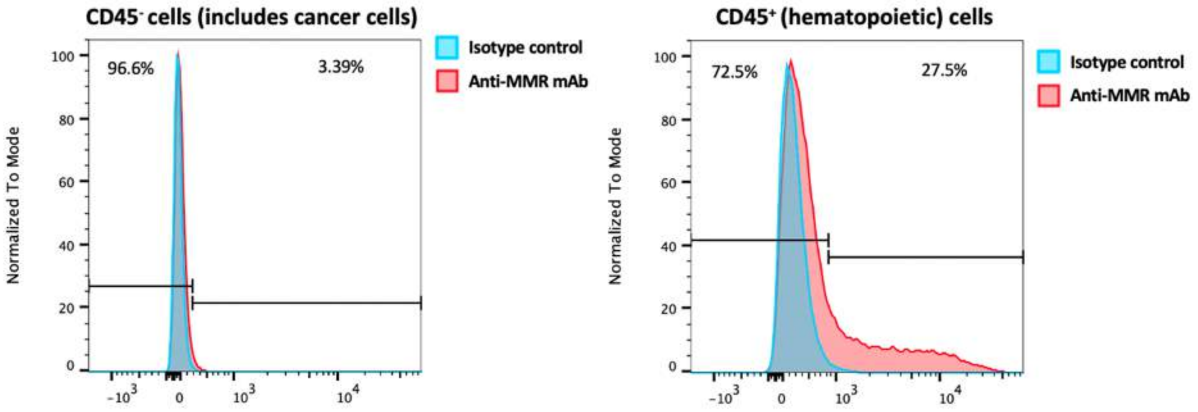

Figure S30: Flow cytometric analysis of the MMR expression within CD45^{-} cells (which include the cancer cells) and the CD45^{+} (hematopoietic) cells. C57BL/6 mice were inoculated with LLC-OVA cancer cells and tumors were allowed to grow for 13 days. Afterwards, mice were sacrificed and tumors were collected and processed to single cell suspensions, which were then stained with fluorescently-labeled anti-MMR mAb or the isotype control. After exclusion of debris and doublets and selection of the living cells, MMR expression histograms of CD45^{-} and CD45^{+} cells are shown.

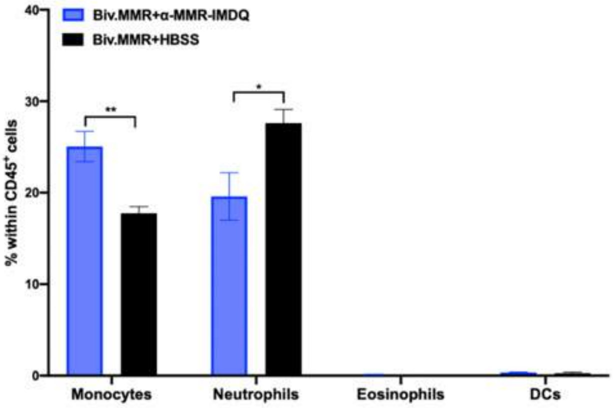

Figure S31: Myeloid cell infiltrate in treated LLC-OVA tumors: LLC-OVA-bearing mice received α-MMR Nb-IMDQ or HBSS, co-injected with a 5-fold molar excess of the bivalent α-MMR Nb (Biv.MMR), according to the schedule shown in Figure 8A. At the end point, mice were sacrificed and tumors processed to single cell suspensions which were then analyzed via FACS. The percentage of Ly6C^{hi} monocytes, neutrophils, eosinophils and DCs within the hematopoietic (CD45^{+}) cells is shown as mean ± SEM of n=4 biological replicates. *P*-values are calculated using an unpaired Student's t-test and significant differences are marked by *: p≤0.05; **: p≤0.01.

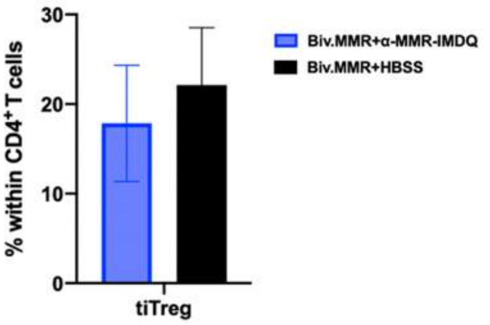

Figure S32: Treg abundance within tumor-infiltrating CD4^{+} T cells in treated LLC-OVA tumors. LLC-OVA-bearing mice received α-MMR Nb-IMDQ or HBSS, co-injected with 5-fold molar excess of the bivalent MMR Nb (Biv.MMR), according to the schedule shown in Figure 8A. At the end point, mice were sacrificed and tumors processed to single cell suspensions which were then analyzed via FACS. The percentage of Treg within tumor-infiltrating CD4^{+} T cells is shown as mean ± SEM of n=4 biological replicates. P-values are calculated using an unpaired Student's t-test and no significant difference was detected.

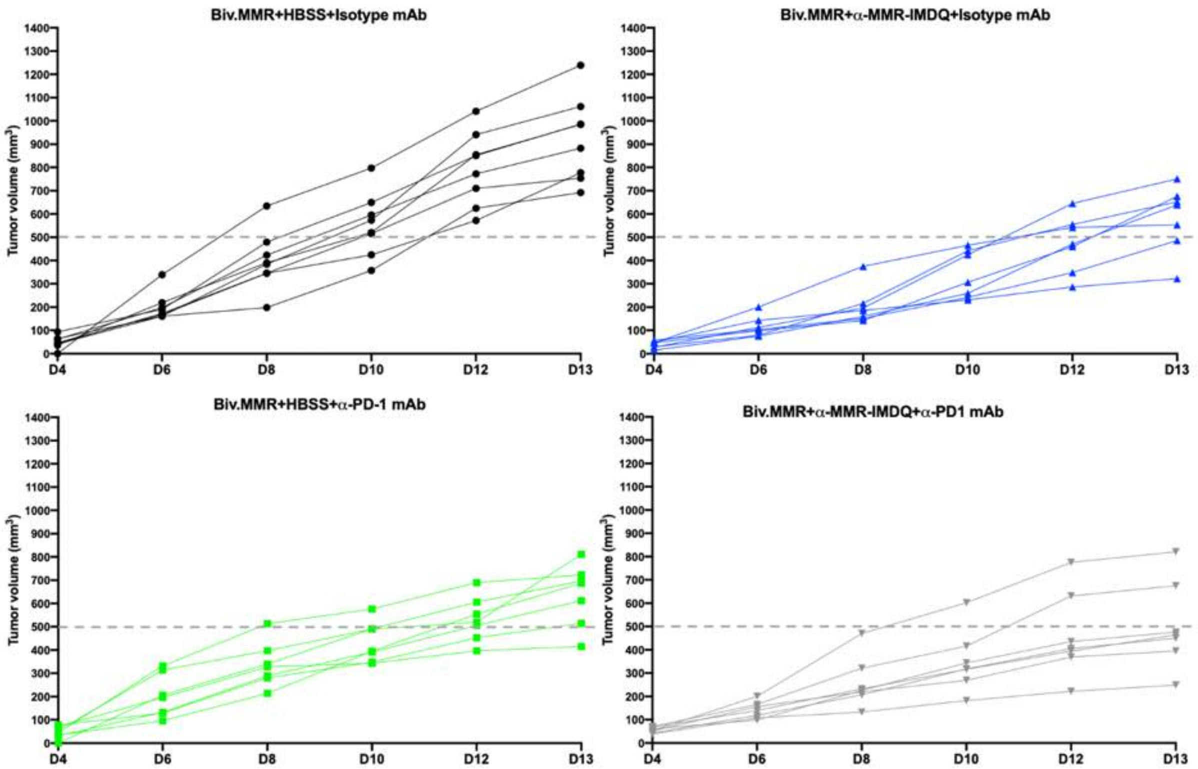

Figure S33: Combination therapy of anti-MMRNb-IMDQ and anti-PD1 immune checkpoint blockade. LLC-OVA-bearing C57BL/6 mice received 0.13 nmol α-MMR Nb-IMDQ (or control HBSS), co-injected with 5-fold molar excess of the bivalent α-MMR Nb (Biv.MMR) on day 5, 8 and 11 after cancer cell inoculation and/or 200 µg α-PD-1 mAb (or control isotype mAb) on day 5, 7, 9 and 11 after cancer cell inoculation. Statistical significance between treatment groups was evaluated using two-way ANOVA. Significant differences were detected between the control group and all treated groups. However, there were no significant differences between the treated groups.

Table S1: Primer sequences for qRT-PCR on sorted MHC-II^{low} TAMs

Gene	Forward Primer	Reverse Primer
Tnf-α	CCTTCACAGAGCAATGACTC	GTCTACTCCCAGGTTCTCTTC
Il-6	GTCTTCTGGAGTACCATAG	GTCAGATACCTGACAACAGG
IL-1β	GTGTGGATCCAAAGCAATAC	GTCTGCTCATTCATGACAAG
Il-12p40	GAAAGACCCTGACCATCACT	CCTTCTCTGCAGACAGAGAC
Nos2	GCTTCTGGTCGATGTCATGAG	TCCACCAGGAGATGTTGAAC
Ptgs2	CAGGCTGAACTTCGAAACAG	CAGCTACGAAAACCCAATCA
Cdh1	ACTTGGGGACAGCAACATCA	GGGTTTAAATCGGCCAGCAT
Cxcl10	TCTGAGTCCTCGCTCAAGTG	CCTTGGGAAGATGGTGGTTA
Ccl5	ACAGGTCAAACTACAACTCCA	TCAGCTCTTAGCAGACATTGG
Ccl8	TCTACGCAGTGCTTCTTTGC	CCACTTCTGTGTGGGGTCTA
Ccl6	ATGTCCAGCTTTGTGGGTTC	AGGTCAGGTTCCGCAGATAA
Mrc1	GCAAATGGAGCCGTCTGTGC	CTCGTGGATCTCCGTGACAC
S12	GGAAGGCATAGCTGCTGGAGGTGT	CCTCGATGACATCCTTGGCCTGAG

Table S2: Uptake values of ^{99m}-labeled α-MMR Nb-IMDQ alone or co-injected with 5-fold excess of cold bivalent α-MMR Nb in LLC-OVA tumor-bearing WT or MMR-KO C57BL/6 mice based on dissection at 3 hours p.i.

**Organs/tissues**	**α-MMR Nb-IMDQ in WT (%IA/g)**	**α-MMR Nb-IMDQ in MMR KO (%IA/g)**	**BCII10 Nb-IMDQ in WT (%IA/g)**	**α-MMR Nb-IMDQ + biv.excess in WT (%IA/g)**
**LLC-OVA tumor-bearing mice**				
Heart	0,203 ± 0,012	0,057 ± 0,006	0,067 ± 0,012	0,07 ± 0,01
Lungs	0,187 ± 0,032	0,133 ± 0,012	0,153 ± 0,012	0,137 ± 0,006
Liver	29,073 ± 0,408	3,073 ± 0,248[a]	6,24 ± 0,295[a]	4,163 ± 0,350[a]
Spleen	1,853 ± 0,071	0,077 ± 0,012[a]	0,1 ± 0,01[a]	0,283 ± 0,015[a]
Pancreas	0,287 ± 0,047	0,027 ± 0,006	0,023 ± 0,006	0,063 ± 0,015
Kidney L	14,547 ± 0,743	38,79 ± 0,948	35,34 ± 2,153	23,723 ± 1,788
Kidney R	16,6 ± 0,501	50,527 ± 19,035	37,783 ± 2,781	25,7 ± 0,487
Small intestine	1,51 ± 0,056	0,42 ± 0,142[b]	0,387 ± 0,029[a]	0,637 ± 0,214[c]
Muscle	0,35 ± 0,03	0,11 ± 0,036	0,11 ± 0,026	0,233 ± 0,042
Bone	2,027 ± 0,351	0,233 ± 0,046[b]	0,283 ± 0,015[c]	0,567 ± 0,090[c]
Lymph node	2,75 ± 0,496	0,447 ± 0,107[c]	0,463 ± 0,07[c]	1,477 ± 0,197[d]
Blood	0,387 ± 0,015	0,863 ± 0,100	0,713 ± 0,046	0,26 ± 0,062
Tumor	1,247 ± 0,218	0,9 ± 0,254[ns]	0,937 ± 0,112[ns]	1,797 ± 0,336[ns]
Tail	0,987 ± 0,389	0,397 ± 0,097[ns]	1,13 ± 0,825[ns]	0,74 ± 0,176[ns]

NOTE: Tracer uptake is expressed as injected activity per gram (%IA/g). Data are mean ± SD (n=3). Significance was tested between α-MMR Nb-IMDQ versus BCII10 Nb-IMDQ and α-MMR Nb-IMDQ injected in WT versus MMR KO and α-MMR Nb-IMDQ versus α-MMR Nb-IMDQ + 5-fold molar excess of bivalent α-MMR Nb in WT for the indicated organ/tissue.

[a]$P < 0.0001$
[b]$P < 0.001$
[c]$P < 0.01$
[d]$P < 0.05$

IMDQ-maleimide Synthesis

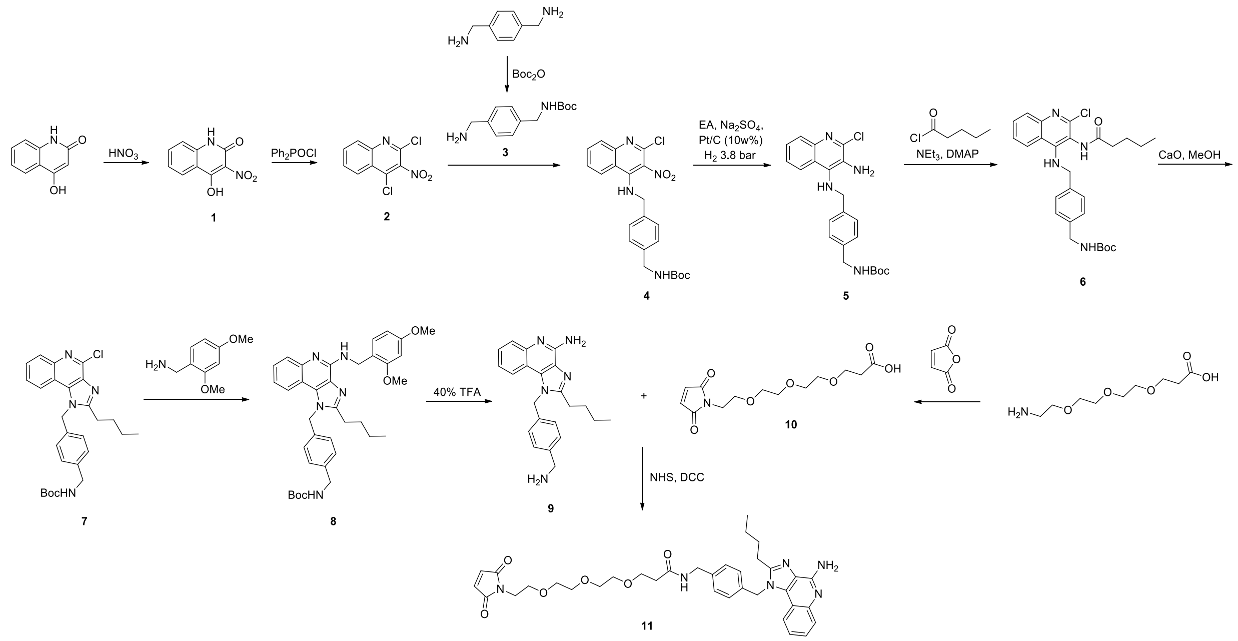

Figure S34: Synthetic scheme for the synthesis of the TL7/8 agonist IMDQ-PEG$_4$-maleimide.

Detailed description of the synthesis of IMDQ-PEG$_4$-maleimide are reported below:

4-Hydroxy-3-nitroquinoline-2-one (1)

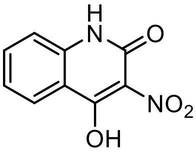

2,4-Dihydroquinoline (15.0 g, 93.0 mmol, 1.0 eq.) was suspended in AcOH (15 mL) and vigorously stirred in an ice bath. 100% Nitric acid (25.0 mL, 595 mmol, 6.4 eq.) was added in 30 min. The reaction mixture was heated to 65 °C. After 30 min no further conversion was observed by TLC and the mixture was introduced into ice water (100 mL). The precipitate was filtered and washed with ice water (2×50 mL) and subsequently dried *in vacuo*.

Yield 16.64 g (80.7 mmol, 87%) yellow, amorphous solid.

MF $C_9H_6N_2O_4$ **MW** 206.1570 [206.0328]

Mp.: >219 °C (combustion).

***R*$_F$:** 0.18 (SiO_2, DCM/MeOH, 4:1).

MS (FD, pos.): m/z: 206.16 $[M]^+$ (calc. 206.03).

^{1}H NMR (400 MHz, DMSO-*d*$_6$, 296 K, COSY, HSQC) δ /ppm = 12.01 (s, 2H, NH), 11.06 (bs, 1H, OH), 8.03 (dd, 1H, $J_{H5,H6}$ = 8.2 Hz, $J_{H5,H7}$ = 1.5 Hz, H-5^Q), 7.64 (ddd, 1H,

$J_{H7,H8}$ = 8.5 Hz, $J_{H7,H6}$ = 7.1 Hz, $J_{H7,H5}$ = 1.5 Hz, H-7^{Q}), 7.33(dd, 1H, $J_{H8,H7}$ = 8.4 Hz, $J_{H8,H6}$ = 1.0 Hz, H-8^{Q}), 7.27 (ddd, 1H, $J_{H6,H5}$ = 8.3 Hz, $J_{H6,H7}$ = 7.1 Hz, $J_{H6,H8}$ = 1.1 Hz, H-6^{Q}).

^{13}C{^{1}H} NMR (101 MHz, DMSO-*d*$_6$, 296 K, HSQC, HMBC) δ /ppm = 156.2 (C-4^{Q}), 155.8 (C-2^{Q}), 138.2 (C-8a^{Q}), 133.2 (C-7^{Q}), 127.3 (C-3^{Q}), 124.5 (C-5^{Q}), 122.4 (C-6^{Q}), 115.9 (C-8^{Q}), 114.0 (C-4a^{Q}).

2,4-Dichloro-3-nitroquinoline (2)

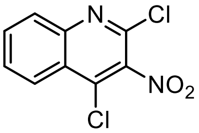

Quinoline **1** (5.00 g, 24.3 mmol, 1.0 eq.) was dissolved under vigorous stirring in diphenylphosphinic chloride (25 mL, 131 mmol, 5.4 eq.) at ambient temperature and then heated to 135 °C and stirred for further 3 h. After 30 min the color had changed to brownish red. The reaction mixture was poured onto crushed ice and water (300 mL) and stirred for 30 min at 0 °C. The precipitate formed was filtered and washed consecutively with water (3×50 mL). The wet crude product was dried *in vacuo* and crystallized from abs. ethanol to yield the title compound.

Yield 3.73 g (0.15 mmol, 63%) pale brown solid.

MF $C_9H_4Cl_2N_2O_2$ **MW** 243.0430 [241.9650]

Mp.: 99-102 °C (EtOH).

***R*$_F$:** 0.46 (SiO_2, CH/EA, 4:1).

MS (FD, pos.): m/z: 242.1 $[M]^+$ (calc. 242.0).

^{1}H NMR (400 MHz, DMSO-*d*$_6$, 296 K, COSY, HSQC) δ /ppm = 8.27 (d, 1H, $J_{H5,H6}$ = 8.3 Hz, H-5^{Q}), 8.10 (m, 1H, H-8^{Q}), 8.09 (m, 1H, H-7^{Q}), 7.94 (ddd, $J_{H6,H5}$ = 8.3 Hz, $J_{H6,H7}$ = 6.4 Hz, $J_{H6,H8}$ = 1.8 Hz, 1H, H-6^{Q}).

^{13}C{^{1}H} NMR (101 MHz, DMSO-*d*$_6$, 296 K, HSQC) δ /ppm = 146.0 (C-8a^{Q}), 141.0 (C-3^{Q}), 138.2 (C-2^{Q}),135.4 (C-4^{Q}), 134.3 (C-7^{Q}), 130.5 (C-6^{Q}), 128.8 (C-8^{Q}), 125.3 (C-5^{Q}), 123.9 (C-4a^{Q}).

***N*-Butyloxycarbonyl-*p*-xylyldiamine (3)**

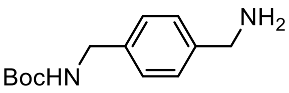

***p*-Xylylenediamine** (9.30 g, 68.3 mmol, 3.0 eq.) and triethylamine (13.8 g, 136.6 mmol, 6.0 eq.) were dissolved in dry DCM (90 mL) and cooled with an ice bath. Boc_2O (5.03 g, 22.8 mmol, 1.0 eq.) was dissolved in dry DCM (20 mL) and added over 5 h. The reaction mixture was brought to room temperature and stirred overnight (20 h). DCM was removed by distillation under reduced pressure. The remainder was taken up into DCM (150 mL). Insoluble particles were removed by vacuum filtration. The filtrate was washed with sat. aqueous $NaHCO_3$ (100 mL), with H_2O (2×100 mL) and with brine (1×100 mL). The organic layer was dried over Na_2SO_4 and the solvents removed *in vacuo* to yield the colorless amorphous product.

Yield 4.33 g (18.3 mmol, 80%) amorphous solid.

MF $C_{13}H_{20}ClN_2O_4$ **MW** 236.3150 [236.1526]

***R*$_F$:** 0.66 (SiO_2, CH/EA, 1:1).

MS (FD, pos.): m/z: 237.29 $[M+H]^+$ (calc. 237.16).

^{1}H NMR (400 MHz, DMSO-*d*$_6$, 296 K, COSY, HSQC) δ /ppm = 7.36 (t, 1H, $J_{NH,CH2}$ = 6.0 Hz, N*H*), 7.26 (d, 2H, $J_{H3,H2}$ = 7.8 Hz, $H3^{ar}$), 7.17 (d, 2H, $J_{H2,H3}$ = 7.9 Hz, $H2^{ar}$), 4.09 (d, 2H, $J_{CH2,NH}$ = 6.2 Hz, C*H*$_2$NH), 3.67 (s, 2H, C*H*$_2$NH$_2$), 1.39 (s, 9H, 3×C*H*$_3$(Boc)).

^{13}C{^{1}H} NMR (101 MHz, DMSO-*d*$_6$, 296 K, HSQC, HMBC) δ /ppm = 155.8 (C=O), 142.7 (C-4), 138.0 (C-1), 126.9 (C-2), 126.7 (C-3), 77.7 (C_q^{Boc}), 45.4 (*C*H$_2$-NH$_2$), 43.2 (*C*H$_2$-NH), 28.3 (*C*H_3^{Boc}).

***N*-Boc-(4-(aminomethyl)benzyl)-2-chloro-3-nitroquinoline-4-amine (4)**

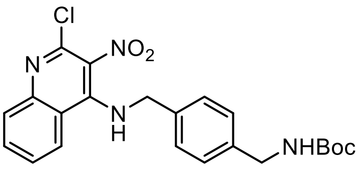

The amine (**3**) (4.05 g, 17.1 mmol, 1.1 eq.) was dissolved in dioxane (160 mL) and 2,4-dichloro-3-nitroquinoline (**2**, 4.05 g, 15.6 mmol, 1.0 eq.) was added in portions under vigorous stirring. The reaction mixture was heated to 65 °C and sodium carbonate (3.31 g, 31.2 mmol, 2.0 eq.) was added. The reaction mixture was stirred

for 1 h. The temperature was adjusted to 70 °C and more sodium carbonate (3.31 g, 31.2 mmol, 2.0 eq.) was added. After 2 h the reaction mixture was cooled to room temperature. The suspension was filtered the remainder was washed with ethyl acetate (50 mL) and the filtrate concentrated *in vacuo*. The crude product was subjected to flash chromatography on silica (toluene/MTBE, 3:1).

Yield 4.78 g (10.8 mmol, 63%) amorphous solid.

MF $C_{22}H_{23}ClN_4O_4$ **MW** 442.9000 [442.1408]

***R*F:** 0.61 (SiO_2, DCM/EA, 1:1).

Mp.: 60–65 °C (MeOH).

^{1}H NMR (400 MHz, DMSO-*d*$_6$, 296 K, COSY, HSQC) δ /ppm = 8.54 (d, 1H, $J_{H5,H6}$ = 8.5 Hz, H-5^Q), 8.48 (t, 1H, $J_{NH,CH2}$ = 6.4 Hz, N*H*^{Q}), 7.84 (m, 2H, H-7, H-8^Q), 7.66 (ddd, 1H, $J_{H6,H5}$ = 8.4 Hz, $J_{H6,H7}$ = 5.2 Hz, $J_{H6,H8}$ = 3.2 Hz, H-6^Q), 7.38 (t, 1H, $J_{NH,CH2}$ = 6.4 Hz, N*H*^{Boc}), 7.20 (m, 4H, C*H*^{XDA}), 4.41 (d, 2H, $J_{CH2,NH}$ = 6.3 Hz, C*H*$_2$NH-Q), 4.09 (d, 2H, $J_{CH2,NH}$ = 6.2 Hz, C*H*$_2$NHBoc), 1.38 (s, 9H, 3×C*H*$_3$(Boc)).

^{13}C{^{1}H} NMR (101 MHz, DMSO-*d*$_6$, 296 K, HSQC, HMBC) δ /ppm = 155.8 (C=O), 145.3 (C-8a^Q), 144.3 (C-4^Q), 141.1 (C-2^Q), 139.5 (C-4^{XDA}), 136.1 (C-1^{XDA}), 132.2 (C-7^Q), 128.6 (C-8^Q), 127.0 (C-6^Q), 126.9 (C-3^{XDA}, C-5^{XDA}), 126.7 (C-3^Q), 126.5 (C-2^{XDA}, C-6^{XDA}), 123.1 (C-5^Q), 119.7 (C-4a), 77.8 (C$_q$^{Boc}), 46.6 (*C*H$_2$NH^Q), 43.0 (*C*H$_2$NHBoc), 28.2 (*C*H$_3$^{Boc}).

***tert*-Butyl (4-(((3-amino-2-chloroquinolin-4-yl)amino)methyl)benzyl)carbamate (5)**

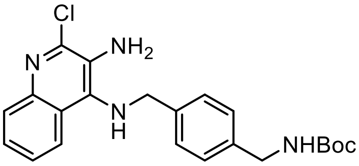

Nitroquinoline **4** (2.82 g, 6.39 mmol) was dissolved in EtOAc (40 mL) and Na_2SO_4 (60 mg) was added. The mixture was put under argon atmosphere before the addition of Pt/C 10wt% (60 mg). The argon was replaced by H_2 and the hydrogenation proceeded at 3.8 bar over 24 h. The catalyst was removed by filtration through a bed of Celite®. The filtrate was concentrated *in vacuo* and the residing oil triturated with Et_2O (30 mL).

Yield 2.189 g (5.30 mmol, 83%) amorphous solid.

MF $C_{22}H_{25}ClN_4O_2$ **MW** 412.9180 [412.1666]

R_F: 0.57 (SiO_2, CH/EA, 1:1).

MS (ESI, pos.): m/z: 413.1728 $[M+H]^+$ (calc. 413.1744).

^{1}H NMR (400 MHz, DMSO-d_6, 296 K, COSY, HSQC, HMBC) δ /ppm = 7.99 (d, 1H, $J_{H5,H6}$ = 7.9 Hz, H-5^Q), 7.66 (d, 1H, $J_{H8,H7}$ = 7.5 Hz, H-8^Q), 7.48–7.26 (m, 4H, H-6, H-7, 2×CH^{xyl}), 7.34 (sb, 1H, NH), 7.20–7.08 (m, 2H, 2×CH^{XDA}), 5.83 (t, 1H, $J_{NH,CH2}$ = 7.3 Hz, NH^Q), 5.08 (s, 2H, NH_2^Q), 4.42 (d, 2H, $J_{CH2,NH}$ = 7.2 Hz, CH_2NH^Q), 4.07 (d, 2H, $J_{CH2,NH}$ = 6.4 Hz, CH_2NHBoc), 1.38 (s, 9H, CH_3(Boc)).

^{13}C{^{1}H} NMR (101 MHz, DMSO-d_6, 296 K, HSQC, HMBC) δ /ppm = 155.8 (C=O), 140.9 (C-2), 140.9 (C-8a), 138.8, 138.7 (2×C_q^{XDA}), 136.3 (C-4), 128.4 (C-3), 128.0 (C-8), 127.4 (C-3^{XDA}, C-5^{XDA}), 127.0 (C-2^{XDA}, C-6^{XDA}), 125.7 (C-7), 125.2 (C-6), 122.9 (C-4a), 122.0 (C-5a), 77.7 (C_q^{Boc}), 49.5 (CH$_2$-NH^Q), 43.1 (CH$_2$-NH^{Boc}), 28.3 (CH$_3^{Boc}$).

***tert*-Butyl (4-(((2-chloro-3-pentanamidoquinolin-4-yl)amino)methyl)benzyl)carbamate (6)**

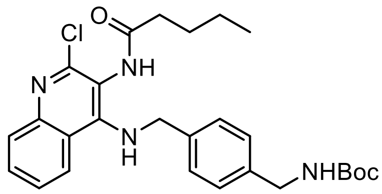

The amine **5** (4.95 g, 12.0 mmol, 1.0 eq.) was dissolved in anhydrous THF (120 mL) together with a catalytic amount DMAP and triethylamine (184 mL, 13.2 mmol, 1.2 eq.). Subsequently valeryl chloride (1.44 g, 12.1 mmol, 1.1 eq.) was added in anhydrous THF (1.5 mL). The reaction mixture was stirred for 3 h at room temperature and then diluted with EtOAc (200 mL). The organic layer was washed with H_2O (3×100 mL) and brine (1×100 mL). The solvents were removed *in vacuo* and the residue subjected to flash chromatography on silica (DCM/MeOH, 100:1→25:1→5:1).

Yield 2.98 g (6.10 mmol, 50%) amorphous solid.

MF $C_{27}H_{33}ClN_4O_3$ **MW** 497.2241 [496.2241]

***R*F:** 0.27 (SiO_2, CH/EA, 1:1).

MS (ESI, pos.): m/z: 497.2313 $[M+H]^+$ (calc. 497.2314).

^{1}H NMR (300 MHz, $CDCl_3$) δ /ppm = 7.89 (d, 1H, J = 8.6 Hz, H-9^{IQ}), 7.83 (d, 1H, J = 8.4 Hz, H-6^{IQ}), 7.59 (t, 1H, J = 7.6 Hz, H-7^{IQ}), 7.36 (t, 1H, J = 7.7 Hz, H-8^{IQ}), 7.24 (s, 4H, H-2^{XDA}, H-3^{XDA}, H-5^{XDA}, H-6^{XDA}), 7.10 (s, 1H, NH^{Boc}), 5.20 (s, 1H, NH^{amid}), 4.92 (s, 1H, NH^{amin}), 4.66 (d, 2H, J = 5.7 Hz, CH_2N^{IQ}), 4.30 (d, 2H, J = 6.0 Hz, CH_2-NHBoc),

2.38 (t, 2H, J = 7.6 Hz, Bu$^{\alpha}$), 1.67 (m, 2H, Bu$^{\beta}$), 1.46 (s, 9H, 3×CH_3(Boc)), 1.37 (m, 2H, Bu$^{\gamma}$), 0.91 (t, 3H, J = 7.3 Hz, Bu$^{\delta}$).

***tert*-Butyl (4-((2-butyl-4-chloro-1*H*-imidazo[4,5-c]quinolin-1-yl)methyl)benzyl)carbamat (7)**

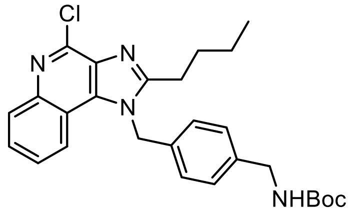

Amide **6** (558 mg, 1.12 mmol, 1.0 eq.) was dissolved in anhydrous MeOH (3 mL) together with activated CaO (55 mg, 0.980 mmol, 1.15 eq.). The resulting suspension was placed in a sealed tube under argon atmosphere. The reaction mixture was stirred for 3 h at 105 °C and subsequently filtered over a bed of cellulose. The filtrate was concentrated *in vacuo* until precipitation of the product was observed. After cooling to room temperature, the crystals were yielded through filtration.

Yield 285 mg (0.59 mmol, 53%) faint yellow crystals.

MF $C_{27}H_{31}ClN_4O_2$ **MW** 479.0210 [478.2136]

***R*F:** 0.42 (SiO_2, CH/EA, 1:1).

MS (ESI, pos.): m/z: 479.2207 $[M+H]^+$ (calc. 479.2209).

^{1}H NMR (400 MHz, DMSO-*d*6, 296 K, COSY, HSQC, HMBC) δ /ppm = 8.09 (d, 1H, $J_{H9,H8}$ = 8.4 Hz, H-9^{IQ}), 8.03 (dd, 1H, $J_{H6,H7}$ = 8.4 Hz, $J_{H6,H8}$ = 1.3 Hz, H-6^{IQ}), 7.64 (t, 2H, $J_{H7,H6}$ = 8.3 Hz, $J_{H7,H8}$ = 7.0 Hz, $J_{H7,H9}$ = 1.3 Hz, H-7^{IQ}), 7.51 (ddd, 1H, $J_{H8,H9}$ = 8.4 Hz, $J_{H8,H7}$ = 7.0 Hz, $J_{H8,H6}$ = 1.3 Hz, H-8^{IQ}), 7.33 (t, 1H, $J_{NH,CH2}$ = 6.2 Hz, NH^{Boc}), 7.17 (d, 2H, $J_{H3,H2}$ = $J_{H5,H6}$ = 8.0 Hz, H-3^{XDA}, H-5^{XDA}), 6.99 (d, 2H, $J_{H2,H3}$ = $J_{H6,H5}$ = 8.2 Hz, H-2^{XDA}, H-6^{XDA}), 5.95 (s, 2H, CH_2N^{IQ}), 4.06 (d, 2H, $J_{CH2,NH}$ = 6.2 Hz, CH_2-NHBoc), 2.97 (d, 2H, $J_{\alpha,\beta}$ = 7.7 Hz, Bu$^{\alpha}$), 1.75 (m, 2H, Bu$^{\beta}$), 1.45–1.29 (m, 11H, Bu$^{\gamma}$, 3×CH_3(Boc)), 0.88 (t, 3H, $J_{\delta,\gamma}$ = 7.4 Hz, Bu$^{\delta}$).

^{13}C{^{1}H} NMR (101 MHz, DMSO-*d*6, 296 K) δ /ppm = 156.7 (C-2^{IQ}), 155.8 (C=O), 143.1 (C-5a^{IQ}), 142.4 (C-4^{IQ}), 139.6 (C-4^{XDA}), 135.3 (C-9b^{IQ}), 134.2 (C-1^{XDA}), 132.9 (C-3a^{IQ}), 129.1 (C-9^{IQ}), 127.7 (C-7^{IQ}), 127.5 (C-3^{XDA}, C-5^{XDA}), 126.7 (C-8^{IQ}), 125.5 (C-2^{XDA}, C-6^{XDA}), 120.9 (C-3a^{IQ}), 117.0 (C-9a^{IQ}), 77.8 (C$_q^{Boc}$), 48.2 (XDA-CH_2-IQ), 43.0 (NHBoc-CH_2-XDA), 29.1, 28.2 (CH_3^{Boc}), 26.3 (Bu$^{\alpha}$), 21.8 (Bu$^{\gamma}$), 13.7 (Bu$^{\delta}$).

***tert*-Butyl (4-((2-butyl-4-((2,4-dimethoxybenzyl)amino)-1H-imidazo[4,5-c]quinolin-1-yl)methyl)benzyl)carbamate (8)**

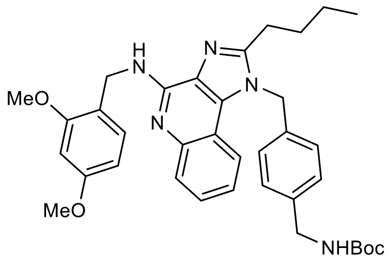

Imidazoquinoline **7** (300 mg, 0.626 mmol, 1.0 eq.) was dissolved in 2,4-dimethoxybenzyl amine (1.06 g, 6.26 mmol, 10 eq.) at 105 °C and stirred for 2 h. The excess reactant was removed *in vacuo* and the crude oil subjected to flash chromatography on silica (CH/EA, 1:1) to yield the product **8** after removal of the eluents *in vacuo*.

Yield 296 mg (0.485 mmol, 77%) amorphous solid.

MF $C_{36}H_{43}N_5O_4$ **MW** 609.7710 [609.3315]

***R*F:** 0.28 (SiO_2, CH/EA, 1:1).

MS (ESI, pos.): m/z: 610.3394 $[M+H]^+$ (calc. 610.3393).

^{1}H NMR (400 MHz, DMSO-*d*6, 296 K, COSY, HSQC, HMBC) δ /ppm =7.78 (dd, $J_{H6,H7}$ = 8.2 Hz, 1H, H-6^{IQ}), 7.57 (dd, $J_{H6,H7}$ = 8.3 Hz 1H, H-6^{IQ}), 7.33–7.31 (m, 2H, H-7^{IQ}, N*H*Boc), 7.21 (d, $J_{H6,H5}$ = 8.3 Hz, 1H, H-6^{DMBA}), 7.17 (d, $J_{H3,H2}$ = $J_{H6,H5}$ = 7.0 Hz, 2H, H-3^{XDA}, H-5^{XDA}), 6.99 (dd, $J_{H2,H3}$ = $J_{H5,H6}$ = 7.1 Hz, 2H, H-2^{XDA}, H-6^{XDA}), 6.92 (t, $J_{NH,CH2}$ = 6.1 Hz, 1H, N*H*^{DMBA}), 6.58 (d, $J_{H3,H6}$ = 2.5 Hz, 1H, H-3^{DMBA}), 6.43 (dd, $J_{H5,H6}$ = 8.4 Hz, $J_{H5,H3}$ = 2.3 Hz, 1H, H-5^{DMBA}), 5.82 (s, 1H, C*H2*-N^{IQ}), 4.70 (d, $J_{NH,CH2}$ = 6.1 Hz, 1H, C*H2*^{DMBA}), 4.08 (m, 1H, C*H2*-NHBoc), 3.86 (s, 3H, C*H*3O), 3.72 (s, 3H, C*H*3O), 2.88 (t, $J_{\alpha,\beta}$ = 7.7 Hz, 2H, Bu$^{\alpha}$), 1.70 (m, 2H, Bu$^{\beta}$), 1.37 (m, 2H, Bu$^{\gamma}$), 1.35 (s, 9H, 3×CH_3(Boc)), 0.86 (t, $J_{\delta,\gamma}$ = 7.4 Hz, 3H, Bu$^{\delta}$).

^{13}C{^{1}H} NMR (101 MHz, DMSO-*d*6, 296 K) δ /ppm = 159.5 (C-4^{DMBA}), 157.8 (C-2^{DMBA}), 155.8 (C=O), 153.6 (C-2^{IQ}), 150.2 (C-4^{IQ}), 144.7 (C-5a^{IQ}), 139.4 (C-4^{XDA}), 134.9 (C-1^{XDA}), 132.3 (C-9b^{IQ}), 128.7 (C-6^{DMBA}), 127.5 (C-3^{XDA}, C-5^{XDA}), 126.8 (C-6^{IQ}), 126.6 (C-7^{IQ}), 126.3 (C-3a^{IQ}), 125.4 (C-2^{XDA}, C-6^{XDA}), 121.0 (C-8^{IQ}), 120.1 (C-1^{DMBA}), 120.0 (C-9^{IQ}), 114.6 (C-9a^{IQ}), 104.2 (C-5^{DMBA}), 98.2 (C-3^{DMBA}), 77.8 (C$_q^{Boc}$), 55.4 (O*C*H3), 55.2 (O*C*H3), 47.9 (*C*H2-N^{IQ}), 43.0 (NHBoc-*C*H2-XDA), 38.3 (*C*H2^{DMBA}), 29.5 (Bu$^{\beta}$), 28.2 (*C*H3^{Boc}), 26.2 (Bu$^{\alpha}$), 21.9 (Bu$^{\gamma}$), 13.7 (Bu$^{\delta}$).

1-(4-(Aminomethyl)benzyl)-2-butyl-1*H*-imidazo[4,5-c]quinoline-4-amine (IMDQ, 9)

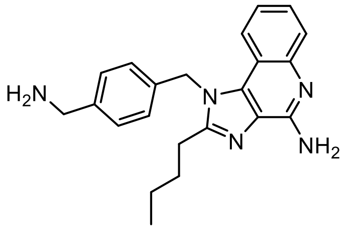

Protected amine **8** (100 mg, 0.163 mmol) was dissolved in TFA in DCM (40v%, 10 mL) and stirred for 30 h at room temperature. The solvent was removed *in vacuo* and the crude oil was taken up into HCl_{aq} (c = 1 mol/L, 200 mL). The resulting suspension was filtered and the pH adjusted to 11 with $NaOH_{aq}$ (50wt%). The aqueous layer was extracted with DCM (6×100 mL). The combined organic layers were dried over Na_2SO_4 and the solvents removed *in vacuo*.

Yield 54 mg (0.150 mmol, 92%) amorphous beige solid.

MF $C_{22}H_{25}N_5$ **MW** 359.4770 [359.2210]

MS (ESI, pos.): m/z: 360.2185 $[M+H]^+$ (calc. 360.2183).

^{1}H NMR (600 MHz, DMSO-*d*$_6$, 294 K, COSY, HSQC, HMBC) δ /ppm = 7.78 (dd, $J_{H6,H7}$ = 8.3 Hz, $J_{H6,H8}$ = 1.4 Hz, 1H, H-6^{IQ}), 7.57 (dd, $J_{H6,H7}$ = 8.4 Hz, $J_{H6,H8}$ = 1.2 Hz, 1H, H-6^{IQ}), 7.32 (m, 1H, H-7^{IQ}), 7.27 (d, $J_{H3,H2}$ = $J_{H5,H6}$ = 7.9 Hz, 2H, H-3^{Bn}, -5^{Bn}), 7.02 (ddd, $J_{H8,H9}$ = 8.3, $J_{H7,H8}$ = 6.9, $J_{H8,H6}$ = 1.3 Hz, 1H, H-8^{IQ}), 6.95 (d, $J_{H3,H2}$ = $J_{H6,H5}$ = 8.0 Hz, 2H, H-2^{Bn}, -6^{Bn}), 6.59 (s, 1H, N*H*$_2$), 5.82 (s, 2H, C*H*$_2$-N^{IQ}), 3.65 (s, 2H, C*H*$_2$-NH$_2$), 2.89 (t, $J_{\alpha,\beta}$ = 7.8 Hz, 2H, 2H, Bu$^{\alpha}$), 1.70 (p, $J_{\beta,\alpha}$ = $J_{\beta,\gamma}$ = 7.6 Hz, 2H, Bu$^{\beta}$), 1.37 (h, $J_{\gamma,\beta}$ = $J_{\gamma,\delta}$ = 7.6 Hz, 2H, Bu$^{\gamma}$), 0.86 (t, $J_{CH3,CH2}$ = 7.4 Hz, 3H, Bu$^{\delta}$).

^{13}C{^{1}H} NMR (151 MHz, DMSO-*d*$_6$, 296 K, HSQC, HMBC) δ /ppm = 153.6 (C-2^{IQ}), 151.8
(C-4^{IQ}), 144.8 (C-5a^{IQ}), 143.1 (C-4^{XDA}), 134.5 (C-1^{XDA}), 132.9 (C-9b^{IQ}), 127.7 (C-2^{XDA}, C-6^{XDA}), 126.5 (C-3a), 126.3 (C-7^{IQ}), 126.1 (C-6^{IQ}), 125.3 (C-2^{XDA}, C-6^{XDA}), 120.9 (C-8^{IQ}), 120.2 (C-9^{IQ}), 114.6 (C-9a^{IQ}), 47.9 (IQ-*C*H$_2$-XDA), 45.1 (*C*H$_2$-NH$_2$), 39.7 (Bu$^{\beta}$), 26.3 (Bu$^{\alpha}$), 21.9 (Bu$^{\gamma}$), 13.8 (Bu$^{\delta}$).

***N*-(ω-Amino-4,7,10-trioxadodecanoic acid)-maleimide (10)**

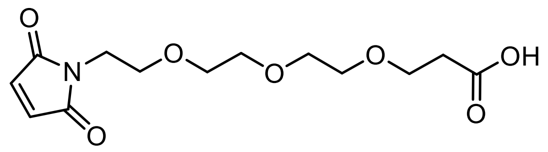

Maleic anhydride (438 mg, 4.47 mmol, 1.1 eq.) was suspended in benzene (1.3 mL). After the addition of 3-(2-(2-(2-aminoethoxy)ethoxy)ethoxy)propanoic acid (900 mg, 4.07 mmol, 1.0 eq.) in benzene (1.3 mL) the reaction mixture was stirred 1 h at 30 °C and diluted with 1,4-dioxane (1 mL). $ZnBr_2$ (1.0 g, 4.47 mmol, 1.1 eq.) and HMDS (984 mg, 6.1 mmol, 1.5 eq.) were added and the resulting mixture refluxed for 2 h. After cooling to room temperature HCl_{aq} (c = 0.5 mol/L, 20 mL) and EtOAc (30 mL) were added. Layers were separated and the aqueous washed with EtOAc (3×30 mL). The organics were dried over Na_2SO_4 and the solvents removed *in vacuo*. The crude was subjected to flash chromatography on silica (DCM/MeOH, 20:1).

Yield 430 mg (1.43 mmol, 35%) yellowish viscous oil.

MF $C_{13}H_{19}NO_7$ **MW** 301.2950 [301.1162]

MS (ESI, pos.): m/z: 324.1048 $[M+Na]^+$ (calc. 324.1054).

^{1}H NMR (400 MHz, DMSO-*d*$_6$, 296 K) δ /ppm = 12.10 (s, 1H, COO*H*), 7.02 (s, 2H, CH^{MI}),

3.63–3.54 (m, 4H, C*H*$_2$-N {3.56}, C*H*$_2$-CH$_2$CO), 3.51 (m, 2H, C*H*$_2$-O), 3.46 (m, 8H, 4×CH$_2$-O), 2.43 (t, 2H, $J_{CH2,CH2}$ = 6.3 Hz, C*H*$_2$-CO).

^{13}C{^{1}H} NMR (101 MHz, DMSO-*d*$_6$, 296 K, HSQC, HMBC) δ /ppm = 172.6 (C=O), 170.9 ($C{=}O^{MI}$), 134.6 (*C*H^{MI}), 69.72, 69.65, 69.61, 69.4, 66.9, 66.2 (6×*C*H$_2$-O), 36.8 (*C*H$_2$-MI), 34.8 (*C*H$_2$-CO).

***N*-(4-((4-Amino-2-butyl-1*H*-imidazo[4,5-c]quinolin-1-yl)methyl)benzyl)-3-(2-(2-(2-(2,5-dioxo-2,5-dihydro-1*H*-pyrrol-1-yl)ethoxy)ethoxy)ethoxy)propenamide (IMDQ-PEG$_4$-maleimide, 11)**

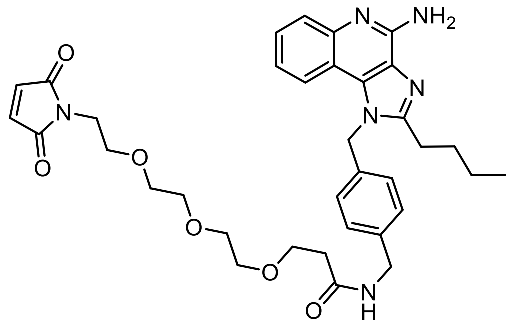

Imidazoquinoline **9** (20 mg, 32.8 µmol, 1.0 eq.) was dissolved together with the linker **10** (10.3 mg, 34.4 µmol, 1.05 eq.) in peptide grade DMF (1 mL). *N*-Hydroxysuccinimide (4.1 mg, 36.0 µmol, 1.1 eq.) was added and the mixture cooled on an ice bath before addition of dicyclohexyl carbodiimide (7.1 mg, 34.4 µmol, 1.05 eq.). The mixture was warmed to room temperature under moderate stirring and kept there for 1 h. The crude was concentrated *in vacuo* and subjected to flash chromatography on silica ($CHCl_3$/MeOH/$NH_3^{conc.}$, 20:1:0.1 → 10:1:0.1) to yield the product.

Yield 20 mg (31.1 µmol, 95%) colorless viscous oil.

MF $C_{35}H_{42}N_6O_6$ **MW** 642.7570 [642.3166]

***R*$_F$:** 0.31 (SiO_2, $CHCl_3$/MeOH, 20:1).

MS (ESI, pos.): m/z: 643.3235 $[M+H]^+$ (calc. 643.3239), 665.3056 $[M+Na]^+$ (calc. 665.3058).

^{1}H NMR (600 MHz, DMSO-*d*$_6$, 294 K, COSY, HSQC, HMBC) δ /ppm = 8.32 (t, 1H, $J_{NH,CH2}$ = 6.0 Hz, NH^{amide}), 7.77 (dd, 1H, $J_{H9,H8}$ = 8.3 Hz, $J_{H9,H7}$ = 1.4 Hz, H-9^{IQ}), 7.56 (dd, 1H, $J_{H6,H7}$ = 8.3, $J_{H6,H8}$ = 1.3 Hz, H-6^{IQ}), 7.33 (ddd, $J_{H7,H6}$ = 8.3, $J_{H7,H8}$ = 6.9 Hz, $J_{H8,H6}$ = 1.4 Hz, 1H, H-7^{IQ}), 7.18 (d, $J_{H3,H2}$ = 8.1 Hz, 2H, H-3^{Bn}, H-5^{Bn}), 7.01 (s, 2H, CH^{MI}), 6.96 (d, $J_{H2,H3}$ = $J_{H6,H5}$ = 8.0 Hz, 2H, H-2^{Bn}, H-6^{Bn}), 6.65 (s$_b$, 2H, NH_2), 5.83 (s, 2H, CH_2-N^{IQ}), 4.21 (d, 2H, $J_{CH2,NH}$ = 6.0 Hz, CH_2-Bn), 3.57 (t, 2H, $J_{CH2,CH2}$ = 6.3 Hz, CH_2^{EG}), 3.54 (t, 2H, $J_{CH2,CH2}$ = 5.8 Hz, CH_2^{EG}), 3.47 (t, 2H, $J_{CH2,CH2}$ = 5.8 Hz, CH_2^{EG}), 3.42–3.32 (m, 8H, CH_2^{EG}), 2.89 (m, 2H, $J_{\alpha,\beta}$ = 8.0 Hz, Bu$^{\alpha}$), 2.32 (t, 2H, $J_{CH2,CH2}$ = 6.3 Hz, CH_2N^{MI}), 1.69 (m, 2H, Bu$^{\beta}$), 1.37 (h, 2H, $J_{\gamma,\beta}$ = $J_{\gamma,\delta}$ = 7.4 Hz, Bu$^{\gamma}$), 0.86 (t, 3H, $J_{CH3,CH2}$ = 7.4 Hz, Bu$^{\delta}$).

^{13}C{^{1}H} NMR (151 MHz, DMSO-*d*$_6$, 294 K, HSQC, HMBC) δ /ppm = 170.9 (C=O^{imide}), 170.1 (C=O^{amide}), 153.6 (C-2^{IQ}), 151.5 (C-4^{IQ}), 144.2 (C-5a^{IQ}), 138.7 (C-4^{XDA}), 134.9

(C-1^{XDA}), 134.5 (C*H*^{MI}), 132.9 (C-9b^{IQ}), 127.5 (C-3^{XDA}, C-5^{XDA}), 126.4 (C-3a), 126.3 (C-7^{IQ}), 125.7 (C-6^{IQ}), 125.3 (C-2^{XDA}, C-6^{XDA}), 120.9 (C-8^{IQ}), 120.1 (C-9^{IQ}), 114.4 (C-9a^{IQ}), 69.6, 69.5, 69.4, 69.3, 66.8, 66.7 (6×CH_2^{EG}), 47.7 (IQ-*C*H_2-XDA), 41.4 (NH-*C*H_2XDA), 36.7 (CH_2^{EG}), 36.0 (*C*H_2-N^{MI}), 29.5 (Bu$^{\beta}$), 26.1(Bu$^{\alpha}$), 21.8 (Bu$^{\gamma}$), 13.6 (Bu$^{\delta}$).

NMR-Spectra

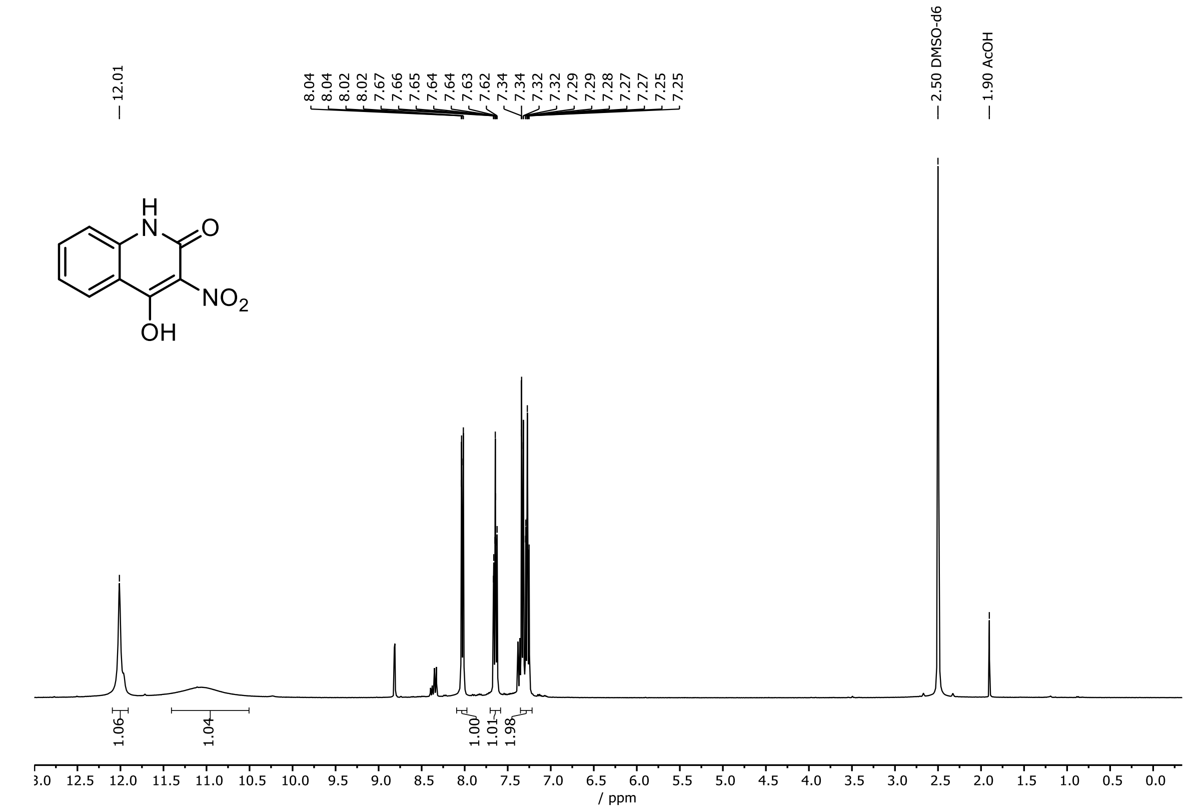

^{1}H NMR (400 MHz, DMSO-*d*$_6$): 2,4-Dihydroxy-3-nitroquinoline (**1**).

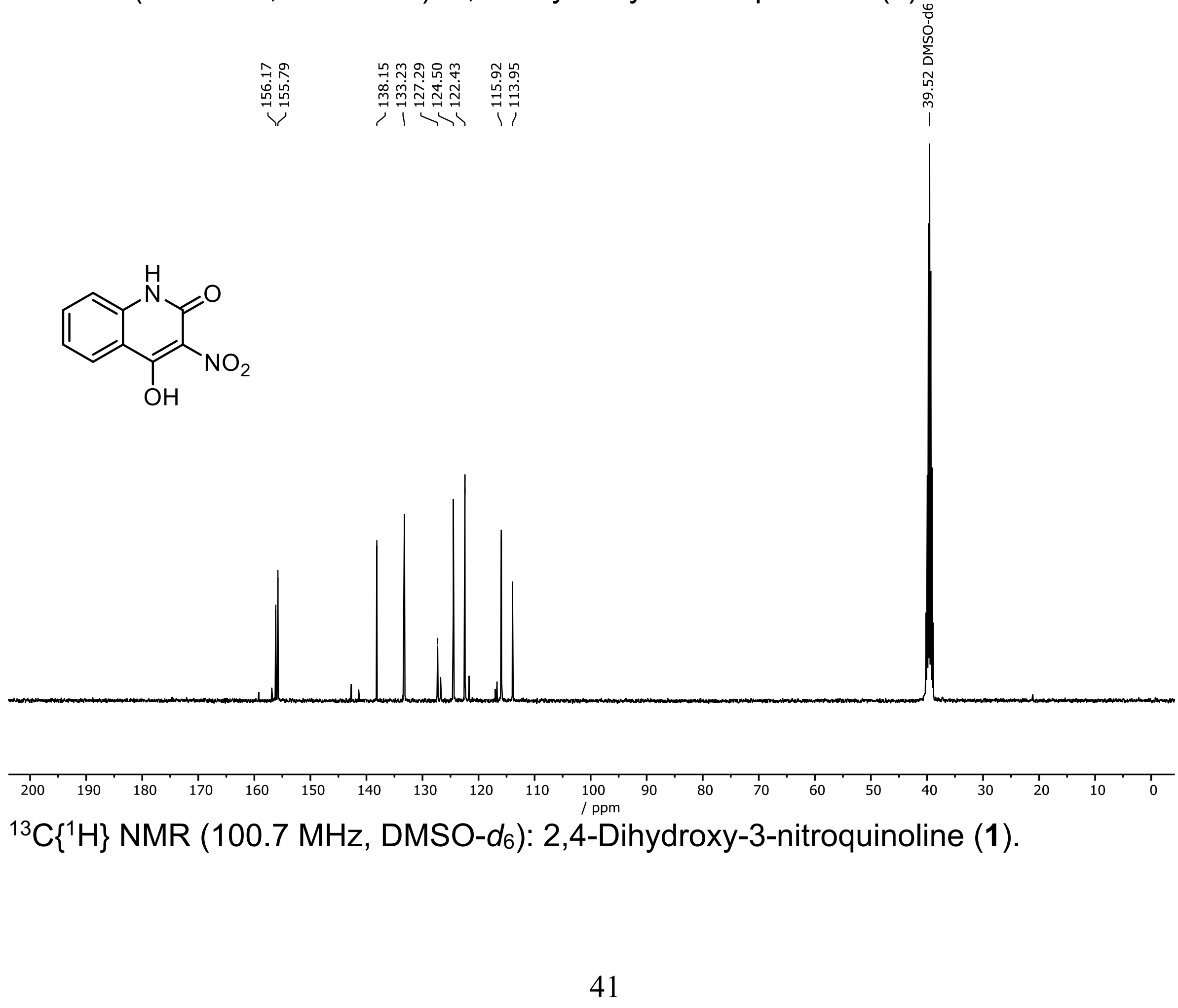

^{13}C{^{1}H} NMR (100.7 MHz, DMSO-*d*$_6$): 2,4-Dihydroxy-3-nitroquinoline (**1**).

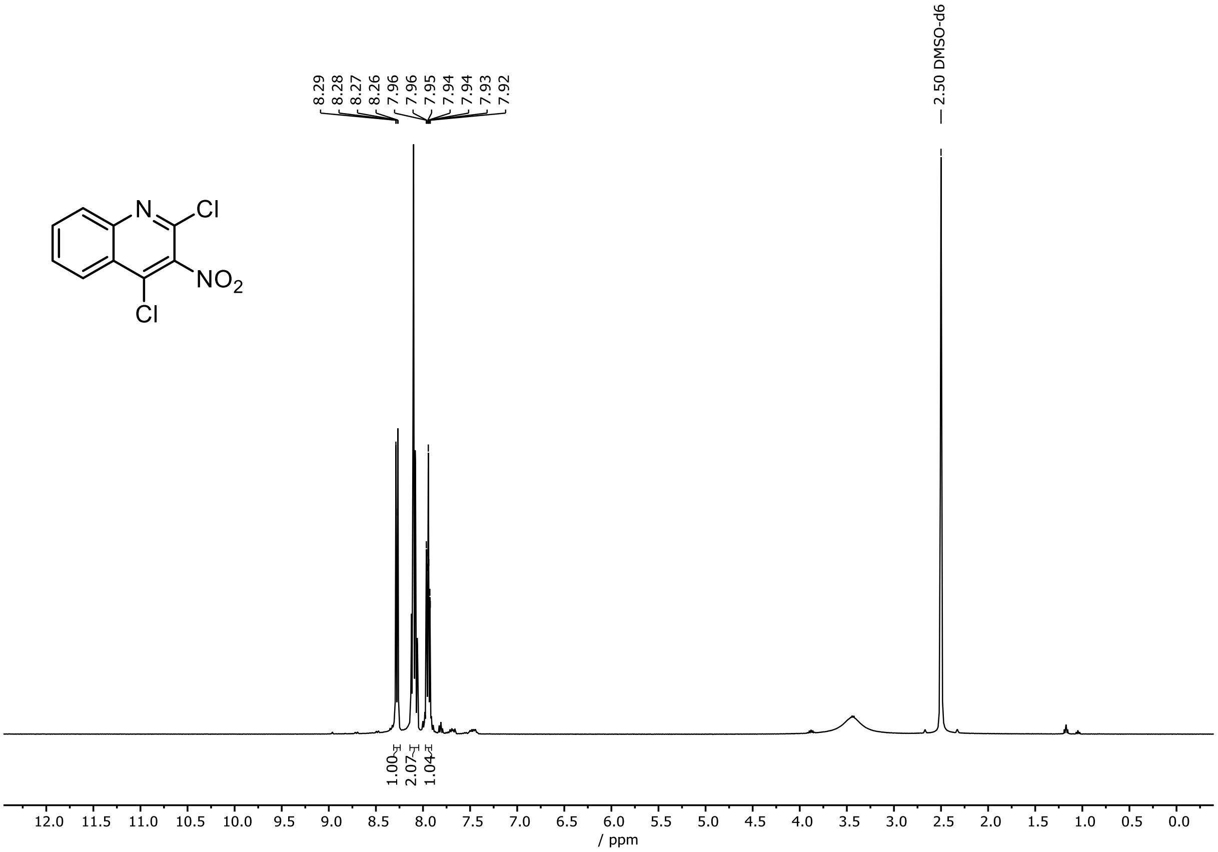

^{1}H NMR (400 MHz, DMSO-*d*$_6$): 2,4-Dichloro-3-nitroquinoline (**2**).

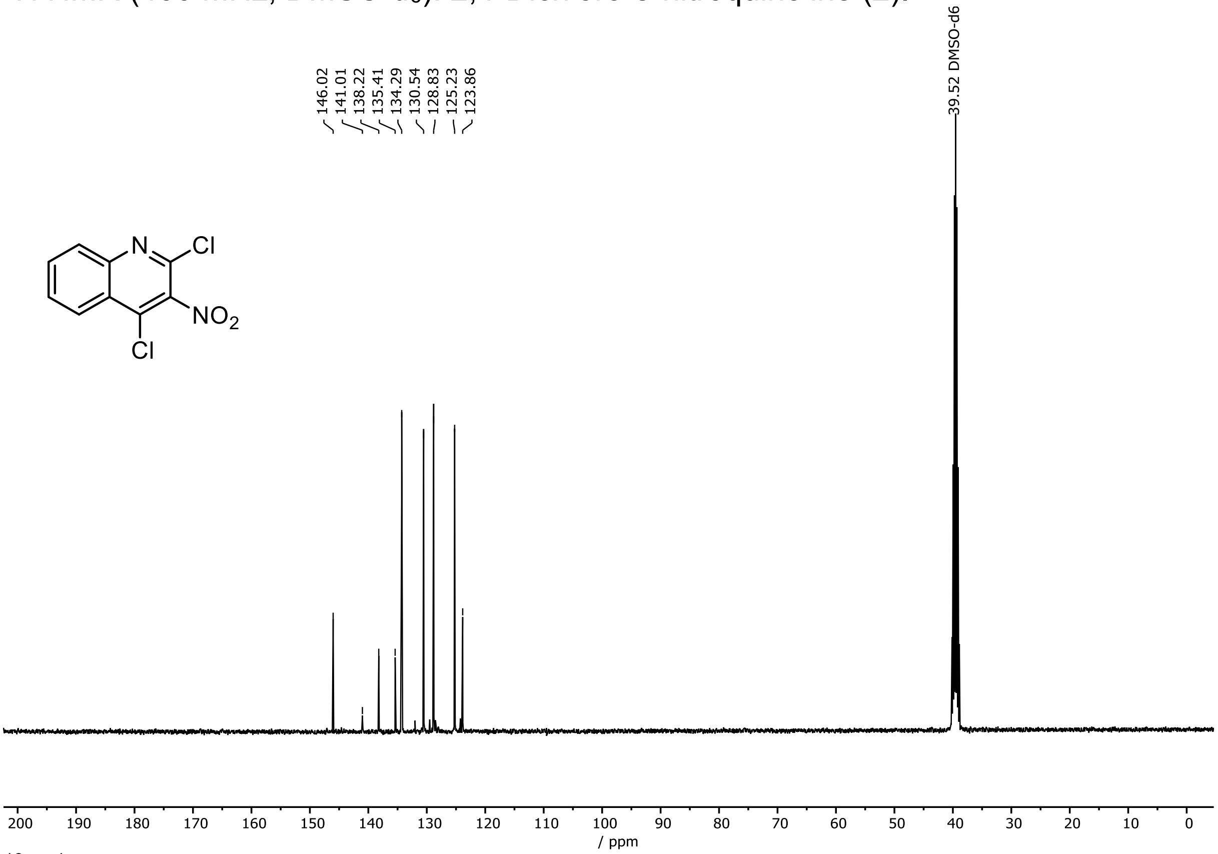

$^{13}C\{^{1}H\}$ NMR (100.7 MHz, DMSO-*d*$_6$): 2,4-Dichloro-3-nitroquinoline (**2**).

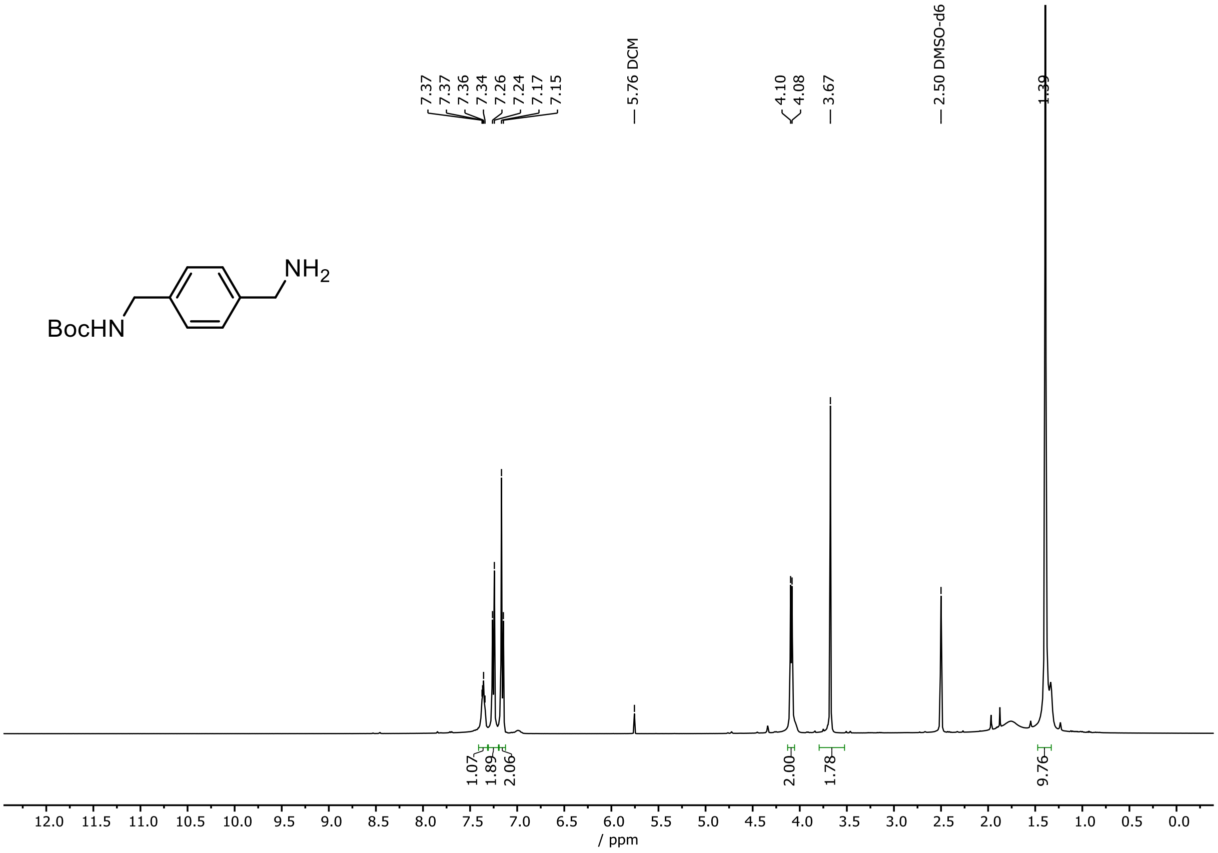

^{1}H NMR (400 MHz, DMSO-d_6): *N*-Butyloxycarbonyl-*p*-xylyldiamine (**3**).

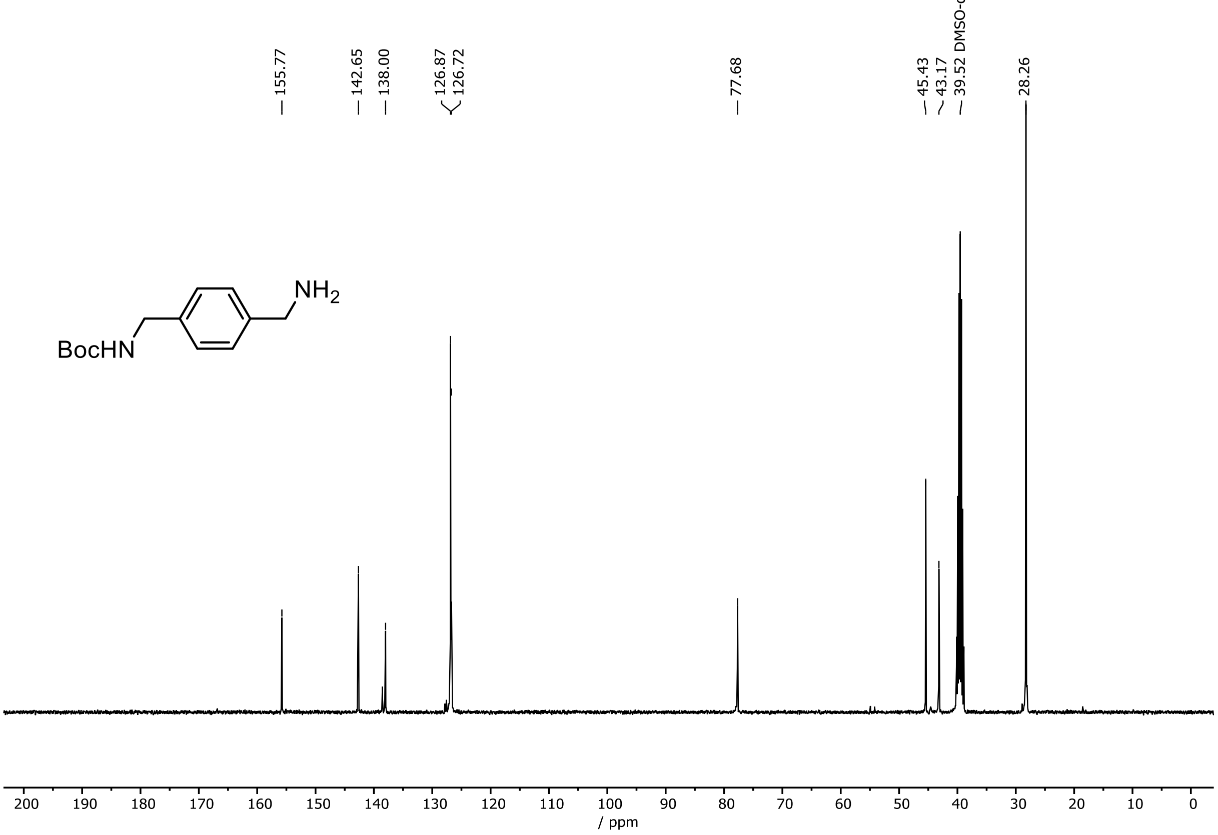

$^{13}C\{^{1}H\}$ NMR (100.7 MHz, DMSO-d_6): *N*-Butyloxycarbonyl-*p*-xylyldiamine (**3**).

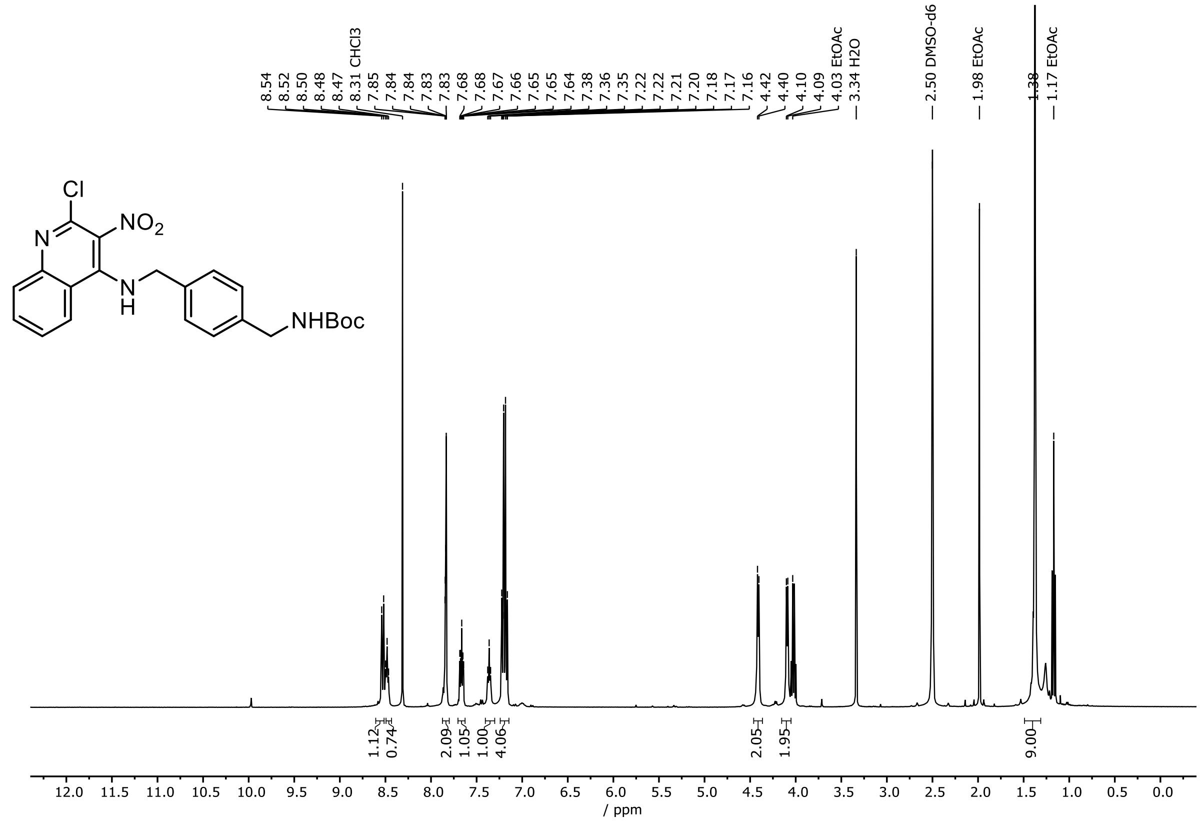

^{1}H NMR (400 MHz, DMSO-*d*$_6$): *N*-Boc-(4-(aminomethyl)benzyl)-2-chloro-3-nitroquinoline-4-amine (**4**).

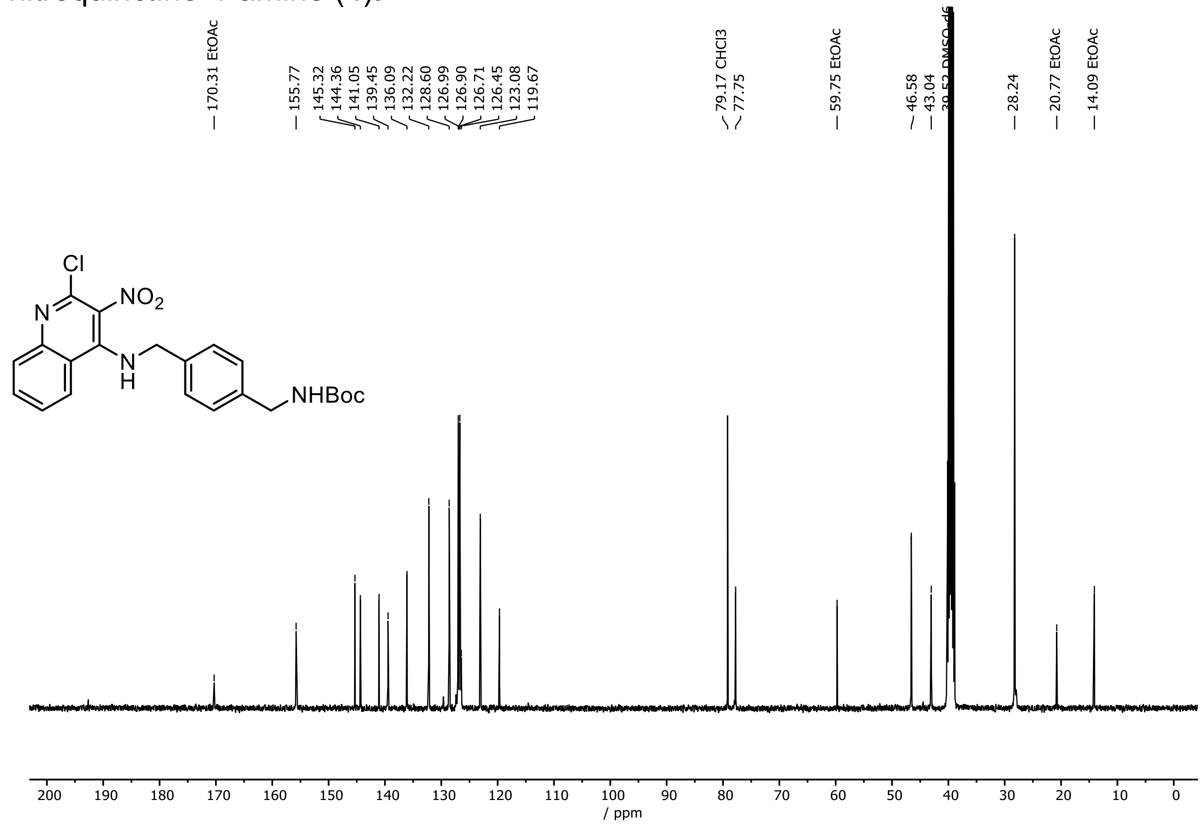

$^{13}C\{^{1}H\}$ NMR (100.7 MHz, DMSO-*d*$_6$): *N*-Boc-(4-(aminomethyl)benzyl)-2-chloro-3-nitro-quinoline-4-amine (**4**).

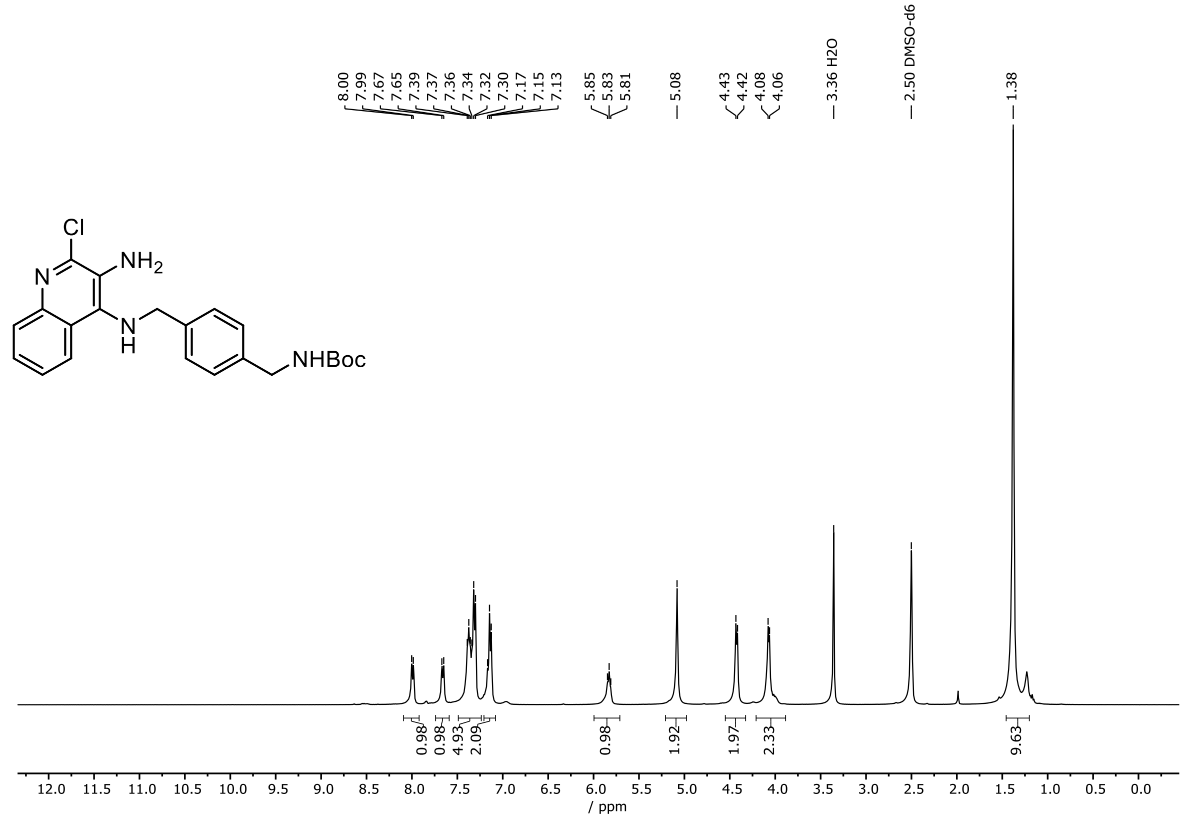

^{1}H NMR (400 MHz, DMSO-d_6): *tert*-Butyl-(4-(((3-amino-2-chloroquinolin-4-yl)amino)methyl) benzyl)carbamate (**5**).

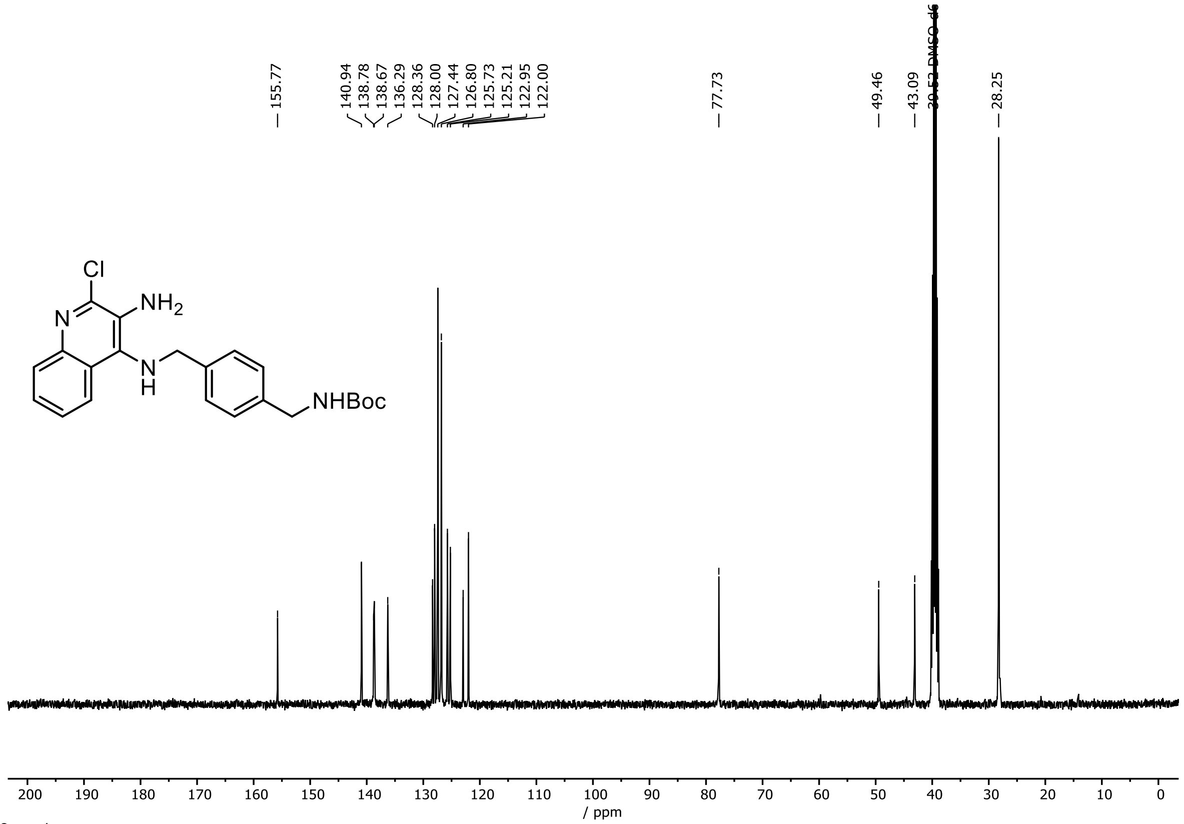

^{13}C{^{1}H} NMR (100.7 MHz, DMSO-d_6): tert-Butyl (4-(((3-amino-2-chloroquinolin-4-yl)amino) methyl) benzyl)carbamate (**5**).

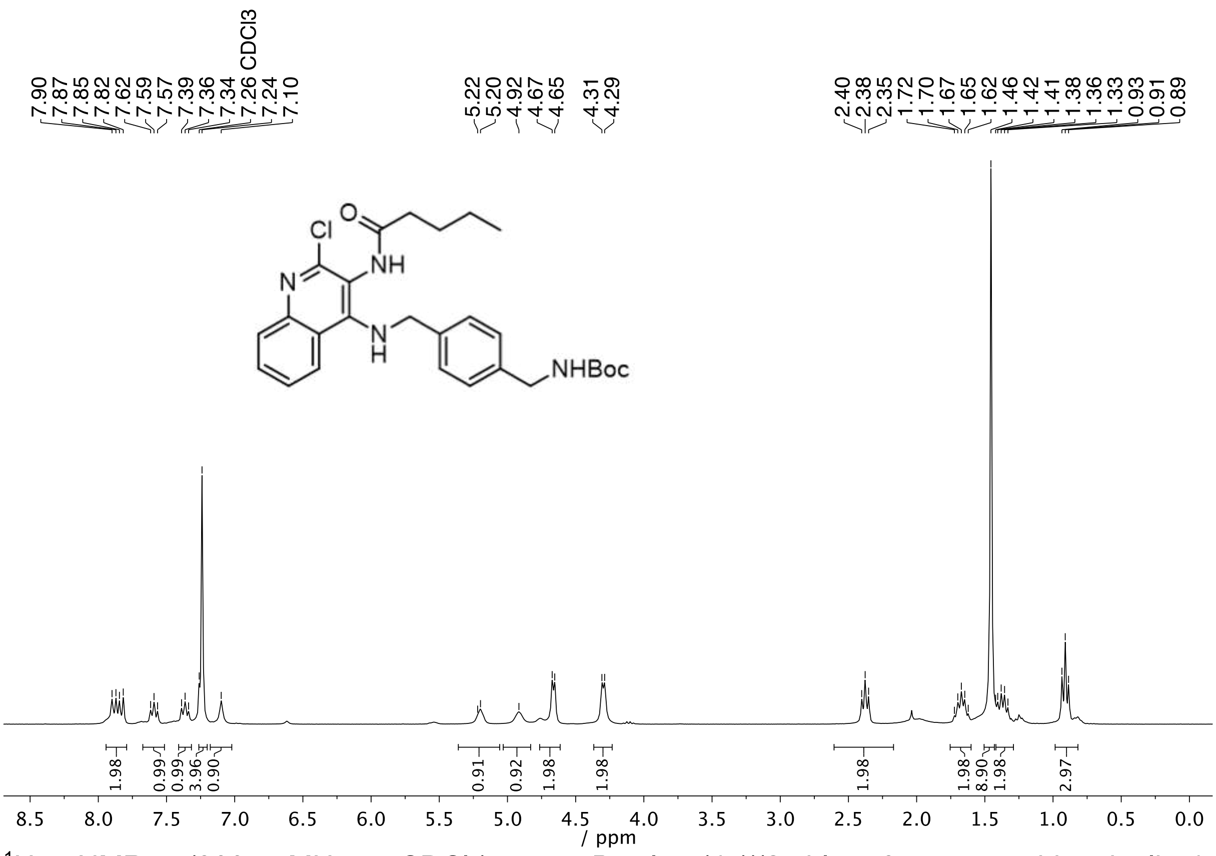

^{1}H NMR (300 MHz, $CDCl_3$): *tert*-Butyl (4-(((2-chloro-3-pentanamidoquinolin-4-yl)amino)methyl)benzyl)carbamate (**6**)

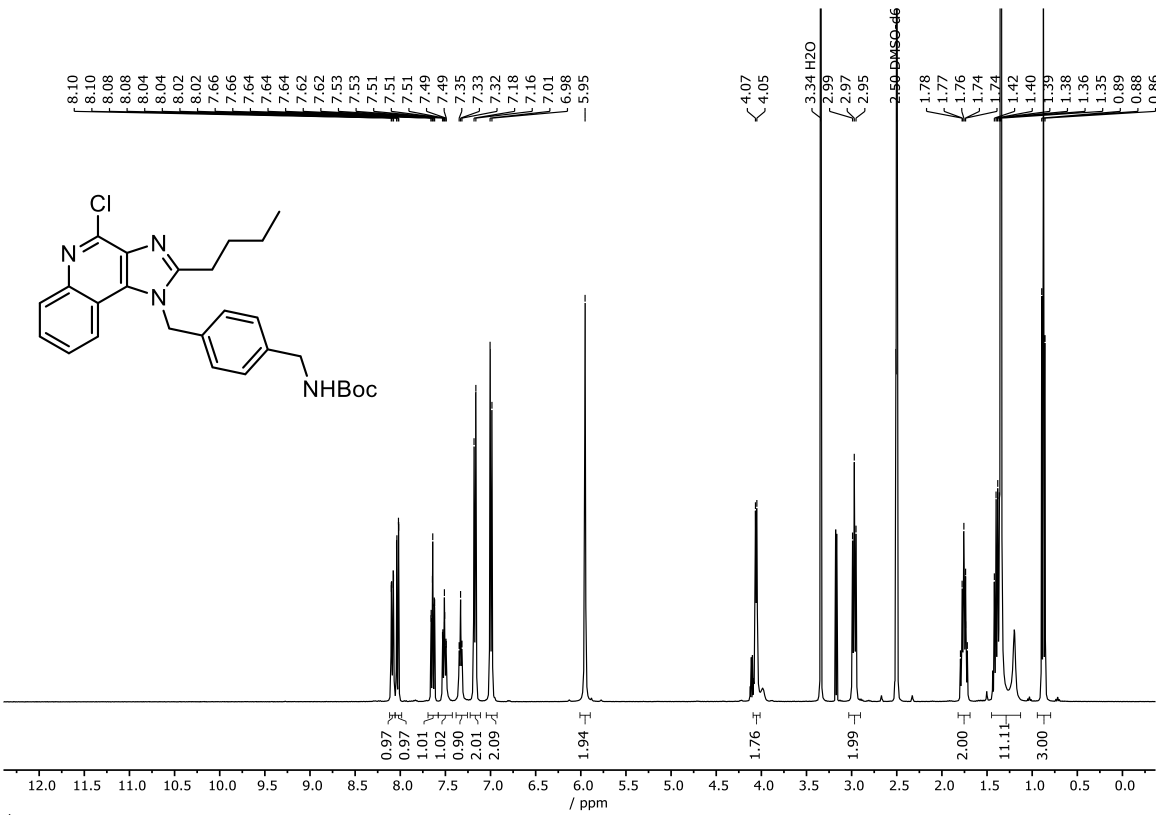

^{1}H NMR (400 MHz, DMSO-d_6): *tert*-Butyl (4-((2-butyl-4-chloro-1*H*-imidazo[4,5-c]quinolin-1-yl)methyl)benzyl)carbamate (**7**).

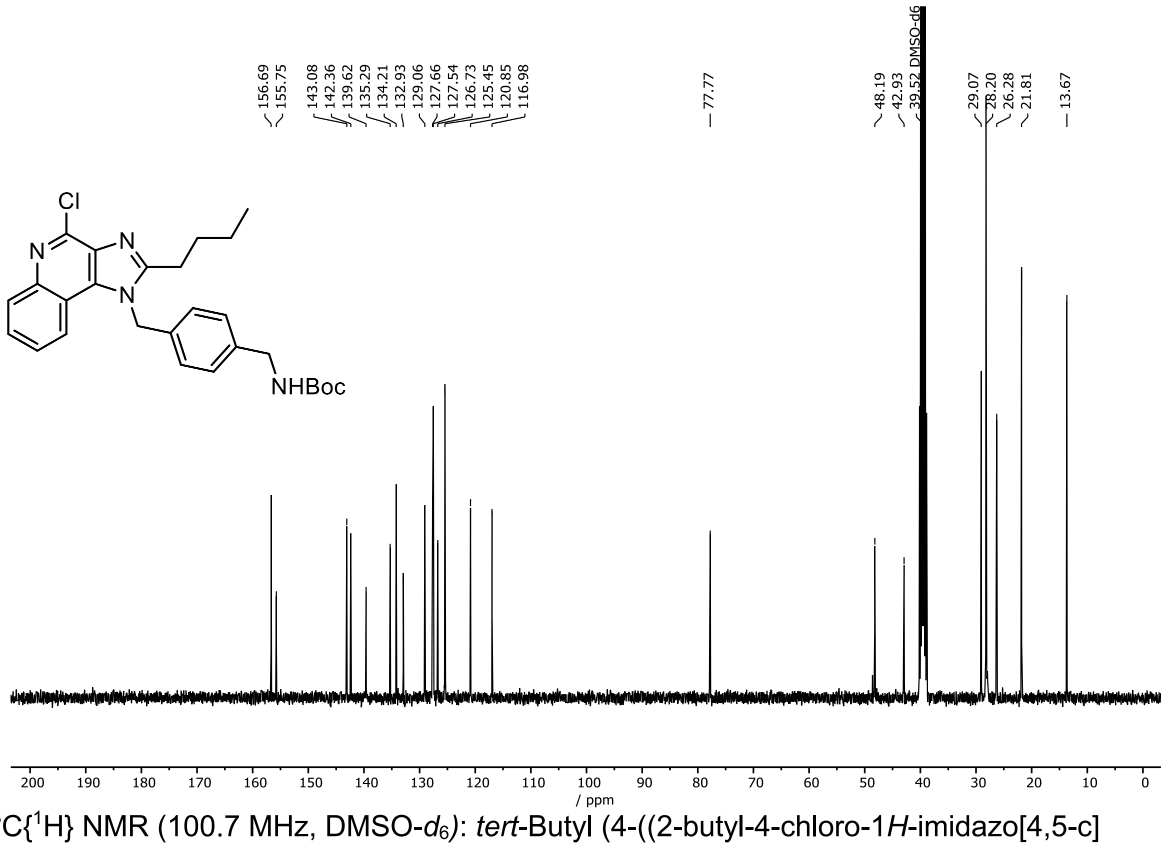

$^{13}C\{^{1}H\}$ NMR (100.7 MHz, DMSO-d_6): *tert*-Butyl (4-((2-butyl-4-chloro-1*H*-imidazo[4,5-c] quinolin-1-yl)methyl)benzyl)carbamate (**7**).

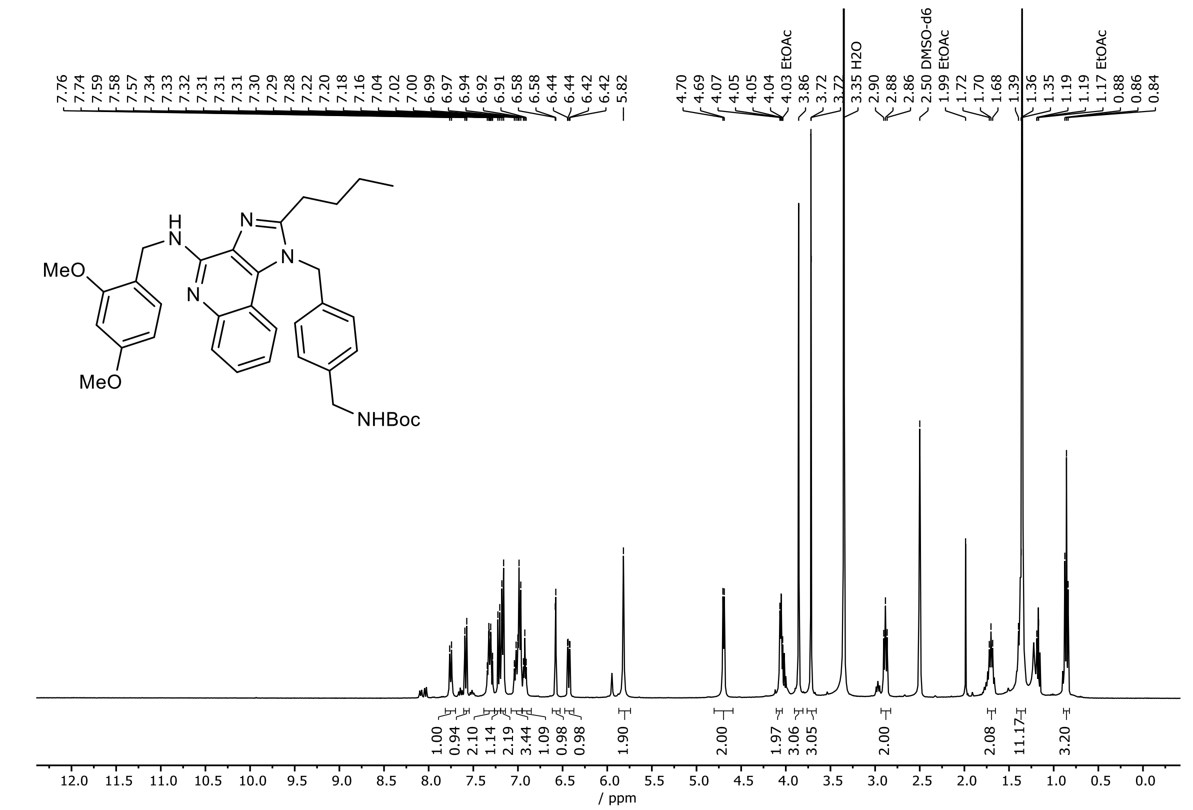

^{1}H NMR (400 MHz, DMSO-d_6): *tert*-Butyl (4-((2-butyl-4-((2,4-dimethoxybenzyl)amino)-1*H*-imidazo[4,5-c]quinolin-1-yl)methyl)benzyl)carbamate (**8**).

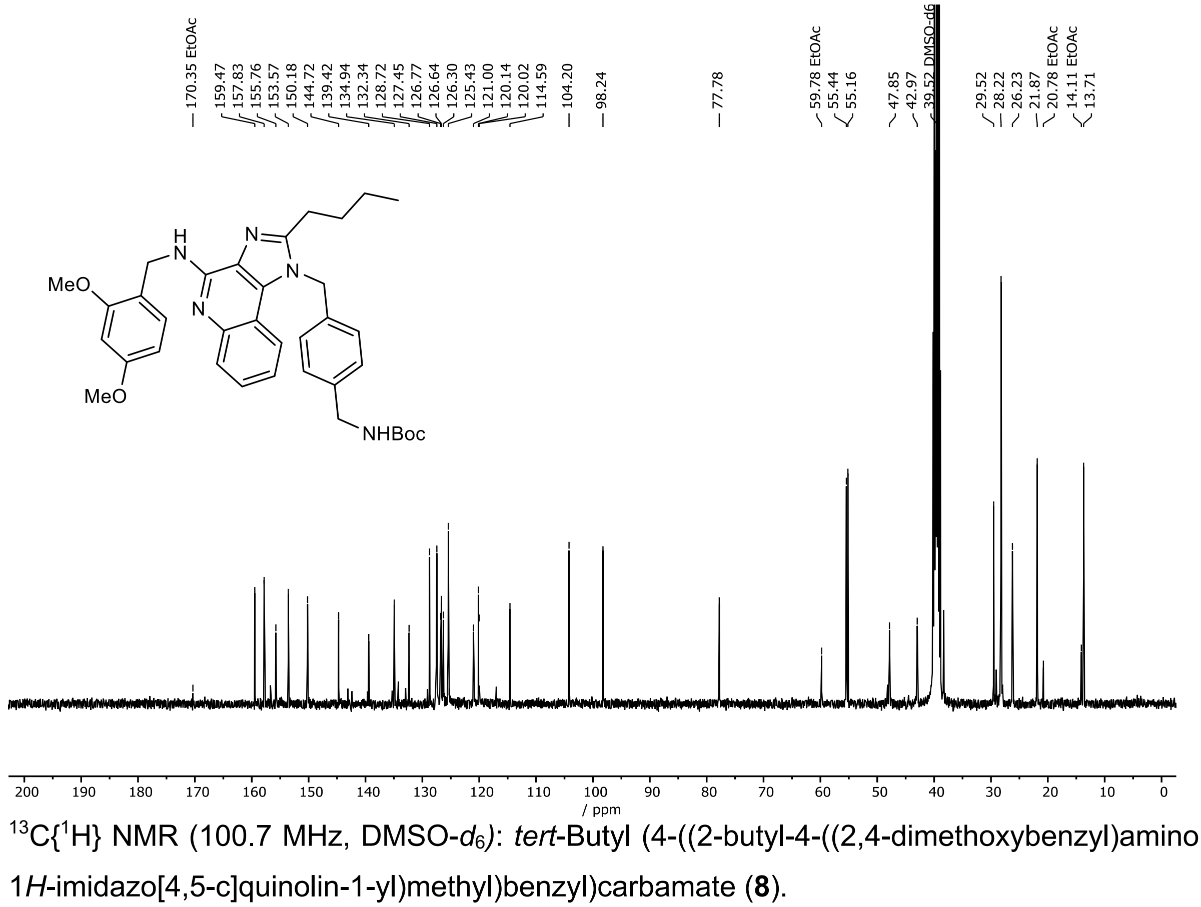

^{13}C{^{1}H} NMR (100.7 MHz, DMSO-d_6): *tert*-Butyl (4-((2-butyl-4-((2,4-dimethoxybenzyl)amino)-1*H*-imidazo[4,5-c]quinolin-1-yl)methyl)benzyl)carbamate (**8**).

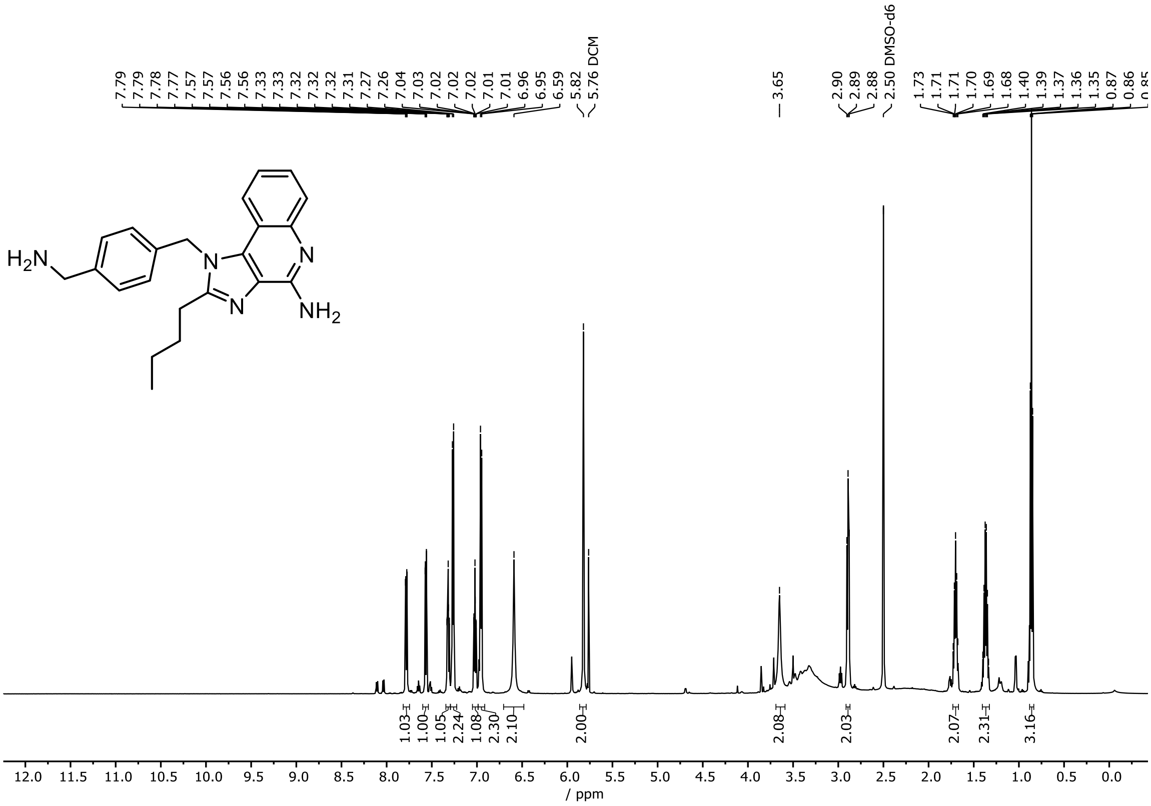

^{1}H NMR (600 MHz, DMSO-d_6): 1-(4-(Aminomethyl)benzyl)-2-butyl-1*H*-imidazo[4,5-c] quinoline-4-amine (**IMDQ, 9**).

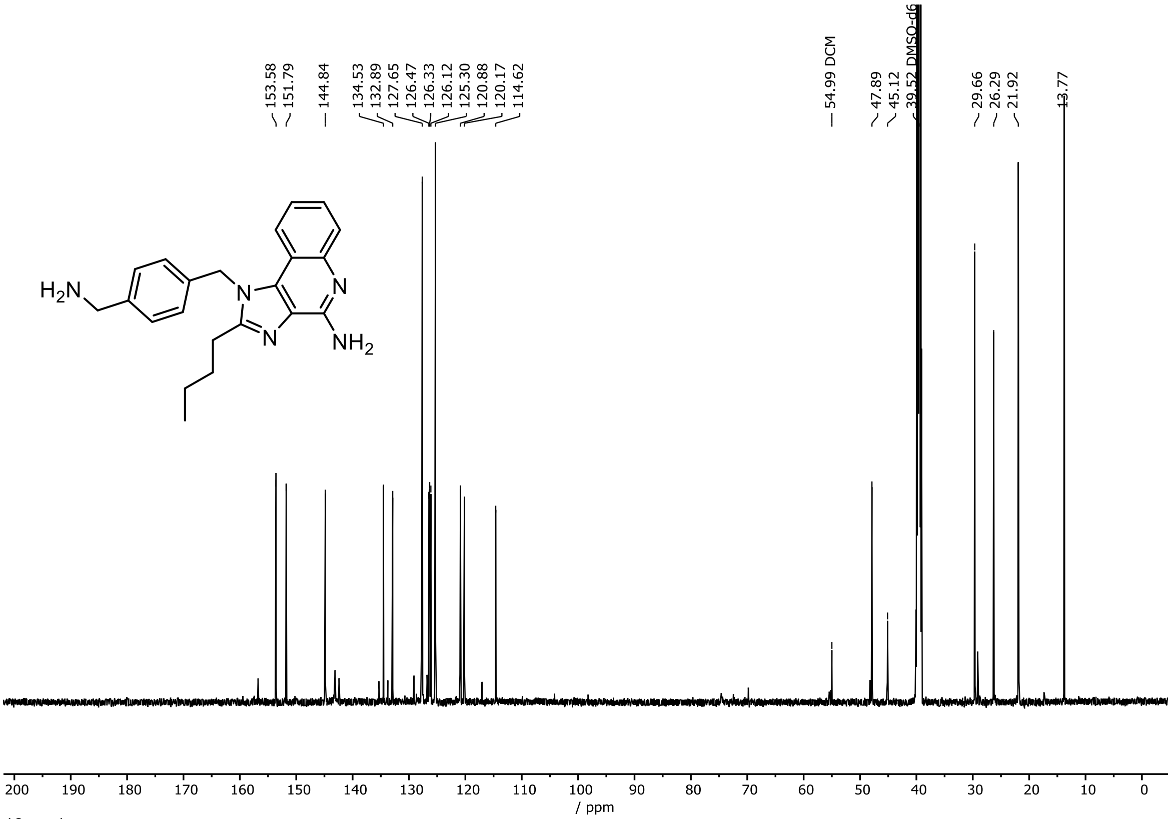

^{13}C{^{1}H} NMR (150.9 MHz, DMSO-d_6): 1-(4-(Aminomethyl)benzyl)-2-butyl-1*H*-imidazo[4,5-c] quinoline-4-amine (**IMDQ, 9**).

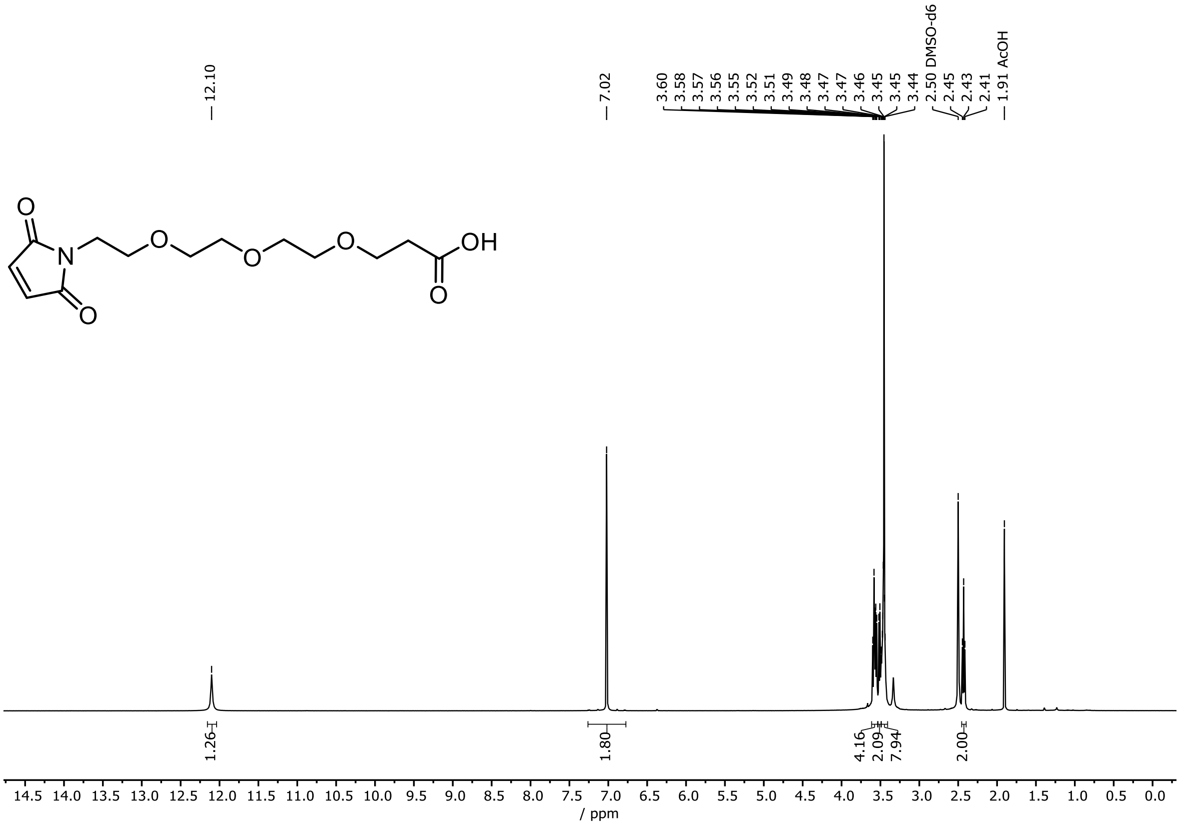

^{1}H NMR (400 MHz, DMSO-d_6): *N*-(ω-Amino-4,7,10-trioxadodecanoic acid)-maleimide (**10**).

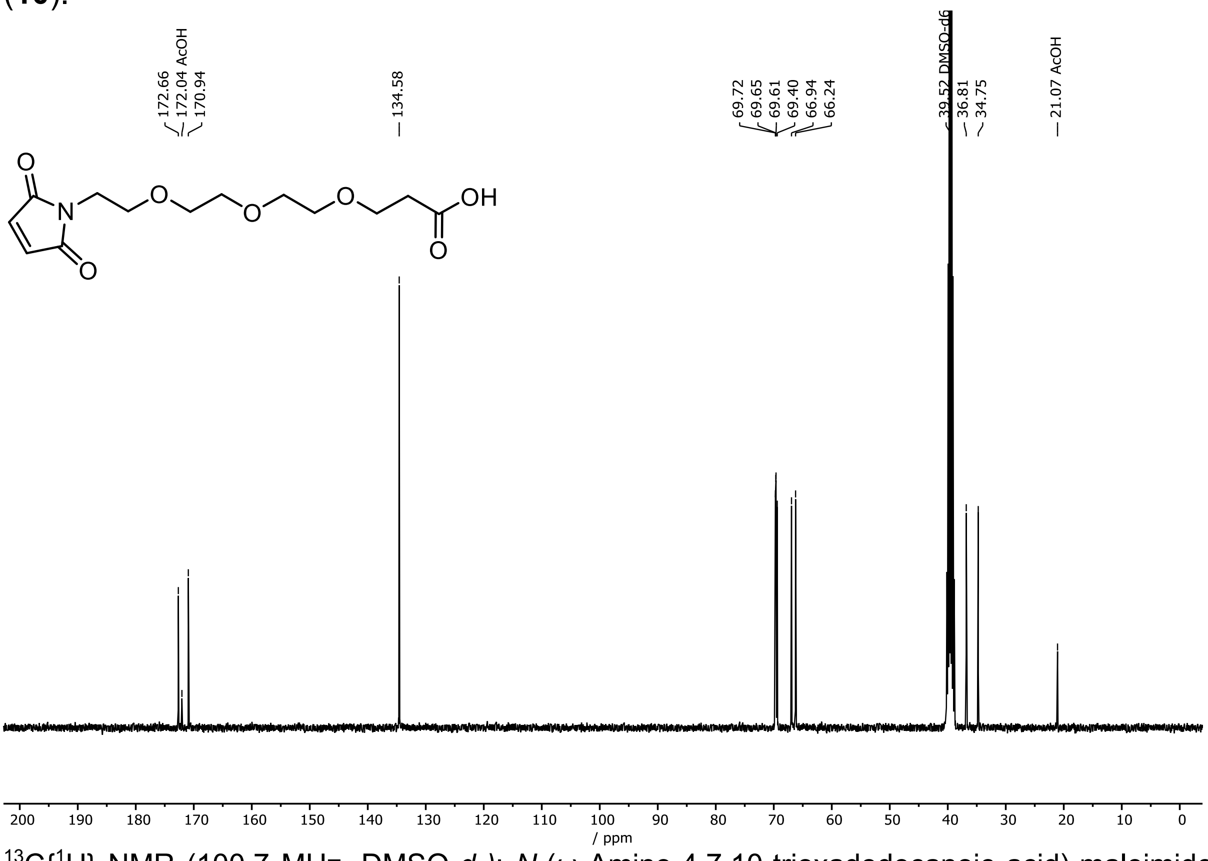

^{13}C{^{1}H} NMR (100.7 MHz, DMSO-d_6): *N*-(ω-Amino-4,7,10-trioxadodecanoic acid)-maleimide (**10**).

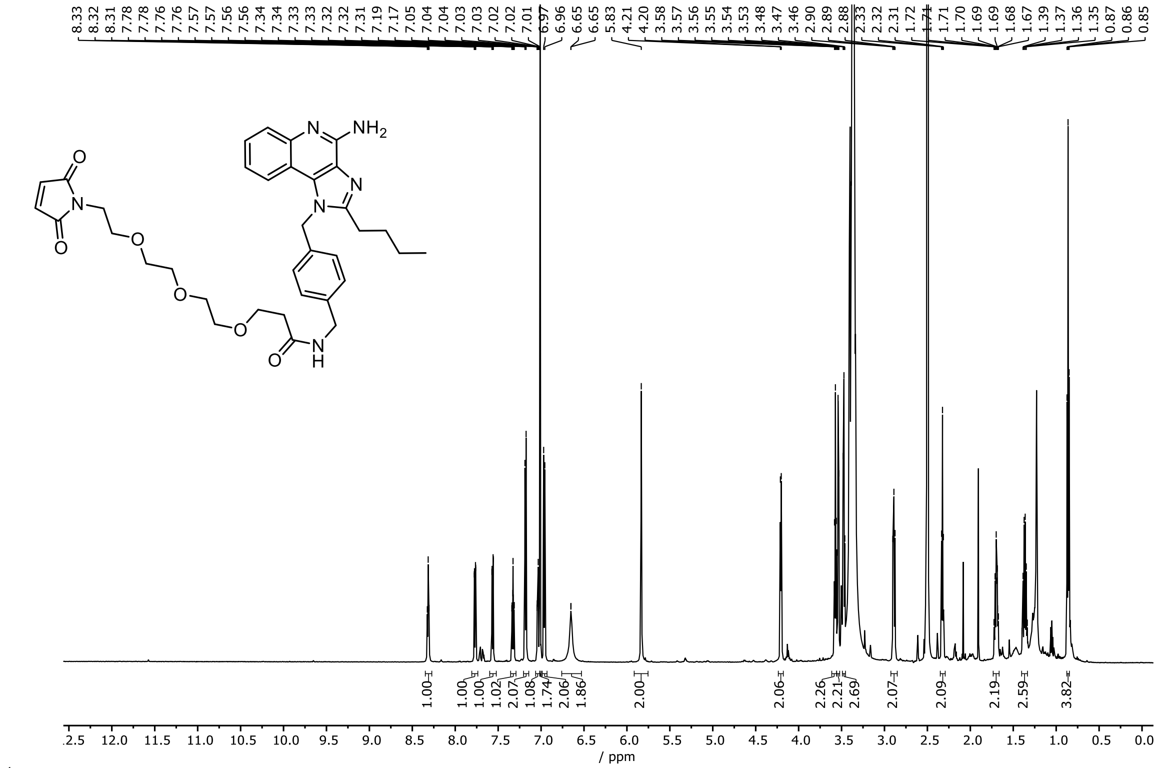

^{1}H NMR (600 MHz, DMSO-d_6): *N*-(4-((4-Amino-2-butyl-1*H*-imidazo[4,5-c]quinolin-1-yl)methyl)benzyl)-3-(2-(2-(2-(2,5-dioxo-2,5-dihydro-1*H*-pyrrol-1-yl)ethoxy)ethoxy)ethoxy) propanamide (**IMDQ-PEG$_4$-maleimide, 11**).

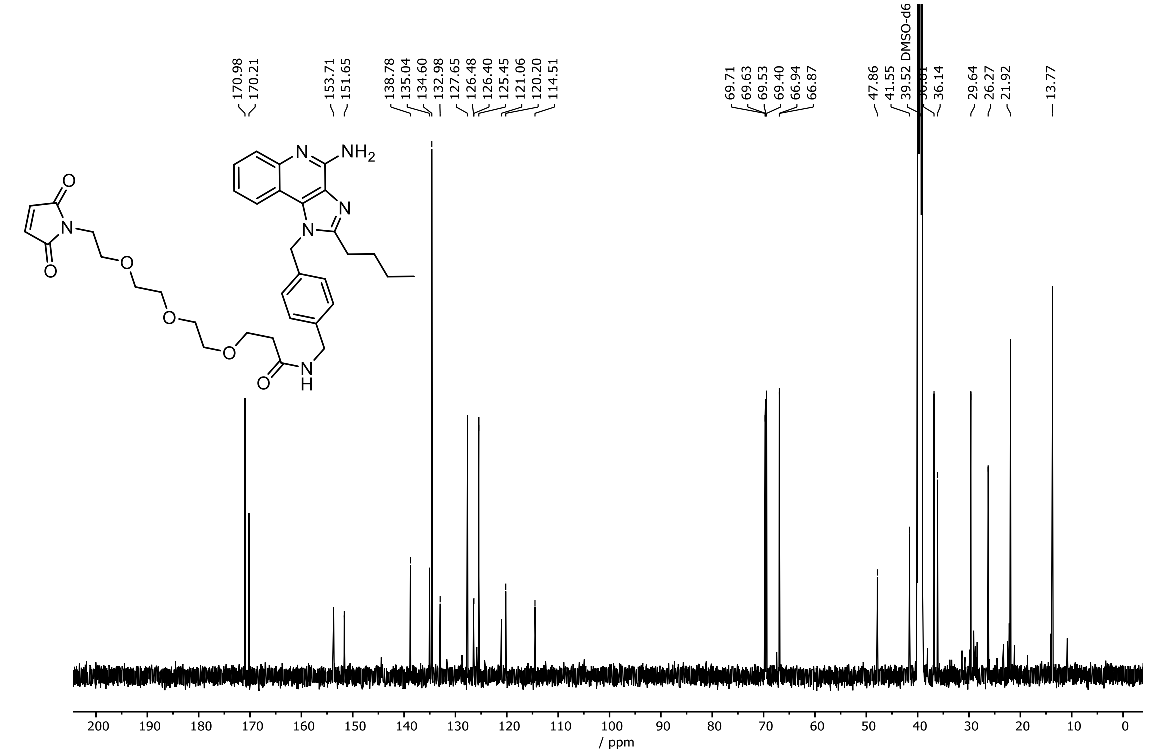

$^{13}C\{^{1}H\}$ NMR (150.9 MHz, DMSO-d_6): *N*-(4-((4-Amino-2-butyl-1*H*-imidazo[4,5-c]quinolin-1-yl)methyl)benzyl)-3-(2-(2-(2-(2,5-dioxo-2,5-dihydro-1*H*-pyrrol-1-yl)ethoxy)ethoxy)ethoxy) propanamide (**IMDQ-PEG$_4$-maleimide, 11**).

References:

1. Movahedi, K. *et al.* Nanobody-based targeting of the macrophage mannose receptor for effective in vivo imaging of tumor-associated macrophages. *Cancer Res.* **72**, 4165–4177 (2012).
2. Conrath, K. E. *et al.* β-Lactamase inhibitors derived from single-domain antibody fragments elicited in the Camelidae. *Antimicrob. Agents Chemother.* **45**, 2807–2812 (2001).
3. Remels, L. M. & De Baetselier, P. C. Characterization of 3LL-tumor variants generated by in vitro macrophage-mediated selection. *Int. J. cancer* **39**, 343–52 (1987).
4. Movahedi, K. *et al.* Different tumor microenvironments contain functionally distinct subsets of macrophages derived from Ly6C(high) monocytes. *Cancer Res.* **70**, 5728–5739 (2010).
5. Liu, Y. *et al.* Nitric Oxide-Independent CTL Suppression during Tumor Progression: Association with Arginase-Producing (M2) Myeloid Cells. *J. Immunol.* **170**, 5064–5074 (2003).
6. Gainkam, L. O. T. *et al.* Comparison of the biodistribution and tumor targeting of two 99mTc-labeled anti-EGFR nanobodies in mice, using pinhole SPECT/micro-CT. *J. Nucl. Med.* **49**, 788–795 (2008).
7. Vaneycken, I. *et al.* In vitro analysis and in vivo tumor targeting of a humanized, grafted Nanobody in mice using pinhole SPECT/micro-CT. *J. Nucl. Med.* **51**, 1099–1106 (2010).
